# Supplementary material for: Developmental synchrony of retinal waves, apoptosis, and angiogenesis in postnatal retina
Source: eLife. 2026 Sep 24;15:RP111419. doi: 10.7554/eLife.111419 (PMC13609470; doi:10.7554/eLife.111419)
Supplement: Supplementary file 2 [file elife-111419-supp1.docx]

| Gene | P Value | Avg_Log2FC | Pct.1 | Pct.2 | P Value Adj | Cluster | Function |
| --- | --- | --- | --- | --- | --- | --- | --- |
| GCLM | 8.95E-117 | 2.416840677 | 0.78 | 0.212 | 1.02E-112 | 0 | Glutamate-cysteine ligase (GCL) serves as a pivotal enzyme in the synthesis of glutathione (GSH), consisting of a catalytic subunit (GCLC) and a modifier subunit (GCLM). Although GCLM does not possess enzymatic activity, it enhances the enzyme's affinity for glutamate and ATP, which in turn increases the concentration of GSH necessary for the inhibition of GCL (McConnachie et al., 2007). GSH is essential for the detoxification of cellular free radicals and provides neuroprotective effects within the brain. A reduction in brain GSH levels is linked to a variety of neurological disorders. While genetic defects in enzymes related to GSH are uncommon, disorders in GSH metabolism are frequently observed in neurodegenerative diseases (Sanchez-Vallejo et al., 2016). GCL functions as the rate-limiting enzyme in the GSH biosynthetic pathway, and the presence of the modifier subunit GCLM enhances the enzyme's ability to synthesize GSH, thereby offering protection against oxidative stress (Yang et al., 2002). |
| FTH1 | 1.45E-125 | 2.028667975 | 1 | 0.996 | 1.65E-121 | 0 | Ferritin is present in the retina, especially within the inner segments of photoreceptors, the retinal pigment epithelium (RPE), the choroid, the inner nuclear layer, and the ganglion cell layer (Gnana-Prakasam et al., 2010). In the mouse retina, ferroportin is located in the RPE, the inner segments of photoreceptors, as well as in the inner and outer plexiform layers, and the ganglion cell layer (Hahn et al., 2004). |
| PRDX1 | 1.66E-85 | 1.770460951 | 0.985 | 0.835 | 1.89E-81 | 0 | Peroxiredoxins, primarily found in neurons within the inner nuclear layer and cone photoreceptors, are essential for the detoxification of hydrogen peroxide, alkyl hydroperoxides, and peroxynitrite in living organisms. Notably, the Prdx1-positive cells in the inner nuclear layer lack Müller cell markers, indicating their neuronal identity. Given their specific location and marker characteristics, these cells are provisionally classified as bipolar cells (Chidlow et al., 2016). |
| FTL | 1.41E-125 | 1.724009177 | 1 | 0.989 | 1.61E-121 | 0 | Ferritin, composed of heavy (FTH1) and light (FTL) chains, plays a vital role in maintaining iron balance within the body. The heavy chain, FTH1, is essential for iron binding and mitigating oxidative damage in the retina. The degeneration of Müller glia cells results in the accumulation of iron in the retinal tissue (Menon et al., 2019).  The light chain, FTL, is predominantly found in amacrine and ganglion cells (Greferath et al., 2009). Ferritin is structured from 24 subunits of both heavy and light chains, allowing it to store iron in a soluble and non-toxic manner, which is critical for iron homeostasis. Alterations in the composition of ferritin subunits can influence the processes of iron uptake and release. Mutations in the FTL gene have been associated with neurodegenerative disorders and hyperferritinemia-cataract syndrome (Greferath et al., 2015).  In the inner retina, FTL expression frequently corresponds with regions exhibiting abnormal Müller cell activity. FTL has been utilized as a marker for investigating neural circuits that respond to various stimuli, such as water regulation, learning and memory, and respiratory control. Additionally, it acts as a reporter for c-fos, a gene that is upregulated during instances of cell death, neural activity, and plasticity (Greferath et al., 2015). |
| LGALS3 | 4.90E-80 | 1.55108324 | 0.649 | 0.167 | 5.58E-76 | 0 | Lectin, galactoside-binding, soluble (Gal-3), which is encoded by the LGALS3 gene, has multifaceted roles in both inflammation and neuroinflammation. In the context of neuroinflammatory diseases like multiple sclerosis, Gal-3 is typically upregulated and can facilitate both pro-inflammatory and anti-inflammatory responses. Notably, a deficiency in Gal-3 correlates with a milder form of autoimmune encephalomyelitis (Jiang et al., 2009). In the retinal environment, Gal-3 is absent in healthy mice but shows increased expression in the retinal endothelium during experimental autoimmune uveitis (EAU), although its specific pathogenic role in EAU has yet to be established (Lipski et al., 2020). Regarding ischemic brain injury, Gal-3 seems to exhibit a dual function: it may act as a pro-inflammatory agent in the early stages while providing protective effects later during degenerative processes (Rahimian et al., 2018). Likewise, in cases of traumatic brain injury, Gal-3 demonstrates both neuroprotective and neurodegenerative properties, which are influenced by the severity and progression of the injury (Nishikawa and Suzuki, 2018). Recent research indicates that Gal-3 may have a pro-inflammatory effect in the retina, as evidenced by diminished neuroinflammation and enhanced axon myelination in Gal-3 knockout mice subjected to diabetes (Mendonça et al., 2018). Within the central nervous system (CNS), Gal-3 is essential for the activation of microglia and is associated with the pathology of Alzheimer's disease (AD) (Puigdellívol et al., 2020). It is primarily expressed by microglia and astrocytes during neuroinflammatory states (Song et al., 2014). Gal-3 can modulate microglial activation, potentially promoting the M1 pro-inflammatory phenotype through the action of IFN-γ and the JAK/STAT signaling pathway (Jeon et al., 2010). As an endogenous ligand for TLR4, Gal-3 interacts with microglial TLR4, thereby maintaining M1 pro-inflammatory responses during acute neuroinflammation (Burgillos et al., 2015). |
| HMOX1 | 6.56E-146 | 1.404022026 | 0.409 | 0.031 | 7.47E-142 | 0 | Heme oxygenase-1 (HMOX1) is an enzyme regulated by redox processes that serves a dual function in the context of neurodegenerative disorders. It is activated in response to oxidative stress, providing cellular protection by converting heme b (iron protoporphyrin IX) into carbon monoxide (CO), ferrous iron, and biliverdin IX, which helps mitigate oxidative stress (Ayer et al., 2016).  In glioma cells, an overabundance of HMOX1 activity can lead to cell death (Meyer et al., 2018). Furthermore, HMOX1 has the potential to induce endoplasmic reticulum (ER) stress, which may result in the death of photoreceptor cells instead of offering protection against oxidative damage. Conversely, HMOX1 has been shown to safeguard photoreceptor cells from light-induced retinal degeneration, and its absence in microglia can prevent neuronal toxicity caused by iron overload (Fernández-Mendívil et al., 2021). Therefore, while HMOX1 functions as an intrinsic protective mechanism against oxidative harm, its excessive activation and the induction of ER stress may pose significant threats to cell survival (Li et al., 2021) |
| IGF11 | 1.15E-78 | 1.362931862 | 0.927 | 0.452 | 1.31E-74 | 0 | Insulin-like growth factor-I (IGF-1) serves as a crucial ligand for the IGF-1 receptor (IGF-1R), which belongs to the tyrosine kinase receptor superfamily. IGF-1 plays a significant role in regulating normal developmental growth through both endocrine and autocrine/paracrine pathways (Bates et al., 1995). It acts as a powerful survival factor for various tissues, enhancing synaptic plasticity, promoting nerve growth, and initiating antiapoptotic signaling pathways within the central nervous system (CNS) (Heemskerk et al., 1999).  In the context of the retina, IGF-1 is recognized as a strong proangiogenic agent (Labandeira-Garcia et al., 2017). Additionally, it acts as a mitogenic factor for microglia, affecting neuroinflammatory responses by altering the phenotype of microglia and facilitating a change in their functional activity. The influence of IGF-1 on neuroinflammatory responses is dependent on the specific context, and a deficiency in IGF-1 can result in chronic low-grade inflammation, which may contribute to retinal degeneration associated with aging (Arroba et al., 2018). |
| LPL | 1.09E-77 | 1.229472789 | 0.691 | 0.201 | 1.24E-73 | 0 | Lipoprotein lipase (LPL) is an enzyme that facilitates the hydrolysis of triglycerides in plasma, which is essential for the release of both essential and nonessential fatty acids necessary for various cellular processes. In ocular tissues, LPL is found to be more abundantly expressed in vascularized areas, underscoring its significance in regulating the availability of fatty acids for retinal functions (Bazan & Scott, 1990). Polyunsaturated fatty acids, which are key constituents of the plasma membranes in the retina, are particularly important. A dietary lack of n-3 fatty acids during developmental stages can result in significant retinal damage, thereby illustrating the critical function of LPL in maintaining a steady supply of these fatty acids for optimal eye development (Casaroli-Marano et al., 1996). |
| BLVRB1 | 5.74E-65 | 1.220497934 | 0.977 | 0.672 | 6.54E-61 | 0 | Broad specificity oxidoreductase, commonly referred to as BLVRB (Biliverdin Reductase B), facilitates the NADPH-dependent reduction of a range of substrates, including various flavins (such as riboflavin, FAD, and FMN), biliverdins, methemoglobin, and pyrroloquinoline quinone (PQQ). This enzyme is crucial in the catabolism of heme, as it metabolizes linear tetrapyrroles and can convert complexed Fe^3+ to Fe^2+ in the presence of FMN and NADPH. Furthermore, BLVRB has been recognized as a potential biomarker for intraplaque hemorrhage and instability of plaques in carotid atherosclerosis (Matic et al., 2018). |
| SLC40A11 | 2.05E-41 | 1.038786468 | 0.544 | 0.191 | 2.33E-37 | 0 | Solute Carrier Family 40, Member 1 (SLC40A1), commonly referred to as ferroportin or IREG1, is an essential protein that plays a pivotal role in the transmembrane transport of ferrous iron. This protein is responsible for facilitating the export of iron across the plasma membrane and is integral to several critical biological processes, including the maintenance of iron ion homeostasis, the regulation of transcription mediated by RNA polymerase II, and the formation of spleen trabeculae. Ferroportin is predominantly found in the basolateral plasma membrane and synaptic vesicles, with expression observed in various tissues, including the central nervous system and extraembryonic structures. Its main role is to transport iron out of cells, which is crucial for sustaining appropriate iron levels. Photoreceptors are particularly vulnerable to damage caused by iron overload, influenced by factors such as elevated retinal oxygen tension, a high metabolic rate, and increased levels of polyunsaturated fatty acids (Baumann et al., 2019). |
| LGALS11 | 1.56E-31 | 1.036434775 | 0.687 | 0.371 | 1.78E-27 | 0 | Galectin-1, a protein encoded by the lectin, galactoside-binding, soluble (LGALS1) gene, serves multiple functions and is essential for the regulation of cell growth, migration, and proliferation. Unlike proteins that specifically bind to designated receptors, Galectin-1 interacts with a variety of receptors based on their glycosylation patterns (Salvatore et al., 1995). Its role in angiogenesis is particularly noteworthy, as it binds to the N-glycans of the VEGF receptor 2 (VEGFR2), thereby activating downstream signaling pathways in vascular endothelial cells. This function has led to the identification of Galectin-1 as a novel ligand for VEGFR2 (Kanda et al., 2015). In the context of the retina, LGALS1 expression is significantly increased in retinal vascular endothelial cells and Müller glial cells during hypoxic conditions. Recent research has underscored the significance of hypoxia-responsive elements (HREs) within the promoter region of LGALS1, which facilitate the hypoxia-induced expression of Galectin-1 in Müller glial cells (Kanda et al., 2020). Additionally, Galectin-1 plays a role in the regulation of apoptosis, cell proliferation, and differentiation. It inhibits the activity of CD45 protein phosphatase, which in turn influences the dephosphorylation of Lyn kinase, and is a potent inducer of T-cell apoptosis (Yamanoto et al., 2021). |
| TALDO1 | 4.67E-55 | 0.973820338 | 0.772 | 0.317 | 5.32E-51 | 0 |  |
| TXNRD1 | 4.64E-27 | 0.968852014 | 0.606 | 0.301 | 5.29E-23 | 0 | Thioredoxin reductase 1 (TXNRD1) plays a significant role in the progression of atherosclerosis and cellular proliferation. It enhances the expression of monocyte chemoattractant protein 1 through the activation of oxidative stress and nuclear factor (NF)-κB signaling in endothelial cells (Pu et al., 2023). TXNRD1 is abundantly found in the nervous systems of mammals and is classified within the family of pyridine nucleotide oxidoreductases (Ren et al., 2017). This protein is essential for preserving the normal thiol redox balance in the nervous system by maintaining thioredoxin (TRX1) and other substrates in their reduced forms, thus contributing significantly to the mitigation of oxidative stress (Yu et al., 2019). |
| MT1M | 2.65E-124 | 0.954622867 | 0.975 | 0.765 | 3.02E-120 | 0 | Metallothioneins (MTs) play a vital role in safeguarding cells from a range of damages, including those resulting from heavy metal toxicity, endoplasmic reticulum stress, and oxidative stress (Liu et al., 2022). They are especially significant in cell populations that respond to stress, such as adult retinal pigment epithelial (RPE) cells, corneal endothelial cells (CEC), Schwann cells, melanocytes, pericytes, and smooth muscle cells (SMCs) (Liu et al., 2022). In contrast, fetal cells, which benefit from protection against oxidative stress provided by the maternal environment, show elevated levels of MT2A expression (Kasahara et al., 2005) (Voigt et al., 2020). Comparative analyses indicate that during the transition from fetal to adult choroidal tissue, there is a decrease in the expression of angiogenesis-related genes in the adult choriocapillaris, alongside an increase in genes linked to extracellular matrix formation in infant pericytes (Collin et al., 2023).  MTs are recognized for their neuroprotective functions, their role in maintaining cellular zinc balance, and their ability to combat oxidative stress and inflammation. Their expression is particularly high in the RPE, where they assist in regulating immune homeostasis (Alvarez et al., 2012). Studies involving MT-deficient mice underscore the essential immunoregulatory functions of MT isoforms across various conditions, including cancer (Pedersen et al., 2009), infectious diseases (Maret and Krezel, 2007), and disorders of the central nervous system (Suemori et al., 2006). |
| PRDX6 | 2.49E-39 | 0.938778673 | 0.347 | 0.087 | 2.84E-35 | 0 | Peroxiredoxins (Prdxs) represent a family of peroxidases that effectively eliminate hydrogen peroxide, alkyl hydroperoxides, and peroxynitrite within living cells. In mammalian tissues, six isoforms of Prdx (Prdx1-6) are expressed, each exhibiting distinct structural characteristics, catalytic mechanisms, and subcellular localization (Hanschmann et al. 2013). Notably, Prdx6 is linked to astrocytes and Müller cells in the retina. Although Müller cells extend throughout the entire thickness of the retina and envelop every neuron, they constitute only approximately 4% of the total cell population in the neuroretina, which accounts for the relatively low levels of Prdx6 mRNA (Jeon et al. 1998).  Prdxs1, -2, and -6 are predominantly found in the cytosol and are essential for the cellular mechanisms that remove peroxides. They are crucial in the cellular defense against oxidative stress. Research indicates that some Prdxs may also act as redox sensors. In conditions of oxidative stress, Prdxs can undergo hyperoxidation due to the interaction with a second peroxide substrate. This reaction leads to the accumulation of oxidized Prdx products and an increase in local hydrogen peroxide levels (Rhee and Woo 2011). While the exact physiological significance of this process remains unclear, it may play a role in preserving redox balance during oxidative stress, triggering downstream redox signaling pathways, and assisting in protein chaperoning (Karplus 2014). Hyperoxidized Prdxs are regarded as potential endogenous markers for oxidative stress (Poynton and Hampton 2014; Chidlow et al., 2016). |
| MIF | 1.46E-122 | 0.935416292 | 0.989 | 0.855 | 1.67E-118 | 0 | Migration inhibitory factor (MIF) serves a pivotal role in the processes of antigen presentation and immune regulation. Its overexpression is known to worsen ocular inflammation, a phenomenon linked to the activation of the Notch signaling pathway, which is essential for both innate and adaptive immune responses. MIF functions as a proinflammatory cytokine, exerting a considerable effect on macrophage activity and T cell activation (Stoffelns et al., 2010). Moreover, MIF enhances the expression of Toll-like receptor 4 (TLR-4) (Nakazawa et al., 2007).  In addition to its role as a hormone produced by the anterior pituitary and a high-activity enzyme, MIF is widely acknowledged for its contribution to inflammatory and autoimmune disorders (Nishihira, 2012; Hoi et al., 2006). It is also involved in the proliferation and survival of neural stem/progenitor cells (NSPCs) (Ohta et al., 2012). Research conducted by Matsuda et al. has underscored MIF's significance in the regulation of ocular immunity and inflammation (Matsuda et al., 1997). Furthermore, MIF's anti-inflammatory properties can influence the actions of glucocorticoids. It is synthesized by various cell types within the brain, including microglia, astrocytes, and neurons (Nasiri et al., 2020). |
| SPARC | 9.64E-208 | 0.898672696 | 0.994 | 0.807 |  | 0 | SPARC (Secreted Protein Acidic and Rich in Cysteine) is prominently expressed in tissues that are undergoing substantial remodeling and is crucial for maintaining endothelial homeostasis by modulating the shape and barrier function of endothelial cells (Lu et al., 2017). Its involvement in angiogenesis is associated with the signaling pathways of VEGF and metalloproteinases (MMPs), where it inhibits the tyrosine phosphorylation of FLT1 and affects both VEGF expression and the activities of MMP7 and MMP9 (Zhang et al., 2012). The production of SPARC in response to hypoxic conditions may play a role in retinal neovascularization (RNV) by promoting central retinal vaso-obliteration (Sobeih et al., 2014). Additionally, SPARC is found in neurogenic regions of the central nervous system (CNS), where it is expressed by radial glia, astrocytes, Schwann cells, and microglia. The expression of SPARC in microglia indicates its potential role in sustaining microglial homeostasis (Vincent et al., 2008). SPARC can be cleaved by proteolytic enzymes, resulting in peptides with specific functions, and it may also influence the expression and activation of these proteases (Brekken and Sage, 2000). It has been proposed that resting microglia may retain SPARC within the cell, releasing it upon activation to aid in cell migration (Rempel et al., 2001). Furthermore, SPARC is involved in regulating the immune response across various tissues, as evidenced by the increased accumulation of macrophages and microglia at injury sites in SPARC-deficient cells (Sangaletti et al., 2011). The expression of SPARC by microglia may help stabilize their non-proliferative state by reducing their responses to growth factors such as VEGF and IGF-1 (O'Donnell et al., 2002). Upon activation, microglia quickly secrete SPARC, which facilitates localized expansion (Llyod-Burton et al., 2013). |
| ESD | 1.04E-17 | 0.848238703 | 0.892 | 0.767 | 1.19E-13 | 0 | Esterase activity is crucial for the early development of Müller cells and ganglion cells within the retina. In the initial differentiation phase, Müller cells, which arise at the vitreal border, demonstrate elevated β-esterase activity. As these cells migrate outward, there is a gradual decline in this enzymatic activity (Bhattacharjee and Sanyal, 1975a). Ganglion cells initially exhibit β-esterase activity, which is soon followed by the emergence of acetylcholinesterase (AChE) activity. As these cells increase in size, their enzyme activity escalates rapidly, with larger ganglion cells displaying significantly higher levels of these enzymes. This early developmental phase also witnesses the emergence of giant and displaced ganglion cells, which bear resemblance to those found in the retinas of various species (Polyak, 1941; Shkolink-Yarros, 1971; Anctil and Ali, 1974).  The development of horizontal cells is similarly characterized by the activity of β-esterase and AChE. As the cell bodies expand and position themselves along the inner side of the developing outer plexiform layer, the activity of these enzymes also rises. An increase in β-esterase activity in Müller cells is associated with heightened carbonic anhydrase activity in both the outer and inner plexiform layers, indicating the growth of lateral processes (Bhattacharjee, 1976). The outer plexiform layer, where synaptic connections form earlier than in the inner plexiform layer (Olney, 1968; Weidman and Kuwabara, 1968), underscores the significance of these developmental changes in enzyme activity within the retina (Bhattacharjee and Sanyal, 1975b). |
| CAPG | 7.21E-36 | 0.788986211 | 0.571 | 0.216 | 8.22E-32 | 0 | Macrophage capping protein (CapG), also referred to as Actin-regulatory protein CAP-G, belongs to the gelsolin/villin family of proteins that regulate actin dynamics. This protein is vital for the modulation of actin behavior within cells. CapG binds reversibly to the barbed ends of F-actin filaments in a manner that is regulated by calcium (Ca2+) and phosphoinositides, thus influencing actin-based motility in non-muscle cells. Notably, in contrast to certain other actin-regulatory proteins, CapG does not cleave existing actin filaments. This capping activity is critical for the regulation of cell motility and structural integrity, especially in endothelial cells, where it facilitates cellular movement (Pellieux et al., 2003; Bharadwaj et al., 2013). |
| VIM | 9.90E-35 | 0.757023691 | 0.544 | 0.204 | 1.13E-30 | 0 | Vimentin (Vim) and glial fibrillary acidic protein (GFAP) are crucial components of the intermediate filament (IF) system in astroglial cells, including Müller glial cells in the retina. These proteins play a significant role in maintaining glial homeostasis and supporting cell structure and function. During activation or reactive gliosis, the expression of Vim and GFAP in Müller cells is notably increased. The presence of these IF proteins is essential for basic glial functions and retinal physiology, and their absence can adversely affect cell survival and retinal health, even in the absence of injury. These findings underscore the importance of intermediate filaments in both maintaining normal glial functions and in the process of reactive gliosis. (Wunderlich et al., 2015). |
| CD9 | 1.61E-126 | 0.728767459 | 1 | 0.841 | 1.83E-122 | 0 | CD9, a member of the tetraspanin family, exhibits low expression levels in the retina at embryonic day 14 (E14), with a notable increase that peaks around postnatal day 12 (P12). It is predominantly found in Müller glia and plays various roles in tissue development. While CD9 is not critical for normal developmental processes, it is essential for the protection of rod photoreceptors (Iwagawa et al., 2020). CD9 is known to work in conjunction with β1 integrins to facilitate the migration of endothelial cells (ECs). This is supported by several findings: low doses of anti-CD9 and anti-β1 monoclonal antibodies (mAbs) exhibit additive effects on lesion repair, both antibodies hinder transmembrane migration towards fibronectin (FN) (Wright et al., 1994), and CD9 and β1 integrins are found to colocalize on the surfaces of ECs (Klein-Soyyer et al., 2000). Importantly, the anti-CD9 mAb (ALMA.1) inhibits EC migration towards both FN and vitronectin (VN), whereas the anti-β1 mAb only impacts migration towards FN. This suggests that CD9 may interact with β1 integrin receptors specific to FN, potentially modulating integrin affinity for their ligands or engaging an unidentified stimulatory pathway, thereby underscoring CD9's critical role in EC migration and possibly in angiogenesis (Klein-Soyyer et al., 2000). In the context of axonal injury, CD9 is highly expressed in extracellular vesicles (EVs) located in the perineuronal space. These EVs, which originate from synapses, are transferred to microglia and show increased expression of phospho-mixed lineage kinase domain-like protein along with EV markers such as CD9, CD63, and flotillin (Salvany et al., 2021). CD9-positive particles are particularly associated with the surfaces of microglial processes near axotomized motor neuron (MN) cell bodies, indicating a mechanism of endocytic trapping of EVs (Salvany et al., 2021). CD9 is widely found in multiple cell types, such as platelets, pre-B cells, mature oligodendrocytes, Schwann cells, astrocytes, and microglia. It is situated along the outer membrane of compact myelin, with its expression emerging at the conclusion of the myelination process. This expression tends to increase with age and continues to be present in the adult brain. Nevertheless, the effects of alterations in CD9 expression under pathological conditions within the nervous system have not been extensively investigated (Salvany et al., 2021). |
| ARHGAP5 | 2.50E-85 | 0.728386053 | 0.866 | 0.691 | 2.85E-81 | 0 | Mice lacking p190B demonstrate abnormalities in the development of the nervous system, including issues with axon formation, neuronal differentiation, and neurogenesis (Matheson et al., 2006). Additionally, these mice show a decrease in adipogenesis (Sordella et al., 2003) and exhibit changes in mammary ductal morphogenesis (Chakravarty et al., 2003). The protein p190RhoGAP-B, which is produced by the ARHGAP5 gene, facilitates alterations in the cytoskeleton by enhancing GTP hydrolysis (Héraud et al., 2019). Furthermore, the expression of the ARHGAP5 gene is increased in human dermal microvascular endothelial cells (HDMECs) when stimulated with TNF and during the process of retinal development (Biname et al., 2016) |
| LIPA | 1.22E-41 | 0.726561753 | 0.452 | 0.132 | 1.39E-37 | 0 | Lysosomal Acid Lipase (LIPA) is an enzyme responsible for the hydrolysis of cholesteryl esters and triglycerides, resulting in the release of free fatty acids and cholesterol within cells. This lysosomal lipolysis is vital as it links lipid metabolism to various cellular activities through the regulatory effects of the lipolytic byproducts. In the retina, the hydrolysis of cholesteryl esters is a critical process that involves three primary enzymes: Nech1, located in the endoplasmic reticulum and microsomes; LIPA, found in lysosomes; and Lipe, present in the cytosol. Together, these enzymes play a significant role in lipid metabolism within retinal cells and the retinal pigment epithelium (RPE), underscoring the importance of lipases for retinal function and overall health. For additional information, refer to the study published in Communications Biology (Yuksel et al., 2023). |
| ZFHX3 | 2.16E-93 | 0.715400585 | 0.897 | 0.719 | 2.46E-89 | 0 | Zinc finger homeobox 3 (ZFHX3) is a transcription factor that is expressed at specific stages of nervous system development. It is crucial for the development and maintenance of the circadian pacemaker function in the suprachiasmatic nucleus (SCN) (Parsons et al., 2015) and is present in early developmental phases of amacrine cells, with its expression diminishing in adulthood (Mayer et al., 2018). ZFHX3 may influence the pupillary response in low-light environments and is involved in non-image-forming visual pathways. It is likely to affect the sensitivity of intrinsically photosensitive retinal ganglion cells (ipRGCs) to light and the mechanisms of photic entrainment. In mutant mice, observed phenotypes may result from impaired GABAergic signaling in amacrine cells or from direct effects on ipRGC functionality (Hughes et al., 2021). |
| RSF1 | 1.54E-30 | 0.715046286 | 0.95 | 0.819 | 1.75E-26 | 0 | Remodeling and Spacing Factor 1 (RSF1) is an integral part of the chromatin remodeling complex, primarily responsible for aiding in the assembly and repositioning of nucleosomes. Its role is vital during mitosis, as it localizes to mitotic kinetochores and engages directly with Polo-like kinase 1 (PLK1) (Lee et al., 2015). The interaction between RSF1 and PLK1 is facilitated by CDK1-mediated phosphorylation at the C-terminal region of RSF1, which establishes a binding site for PLK1. Additionally, PLK1 further phosphorylates RSF1, enhancing the stability of this interaction. RSF1 is also crucial for effective DNA damage repair, operating within a complex that includes SNF2h and undergoing post-translational modifications by ATM kinase to support the repair mechanisms (Min et al., 2018). |
| EMP3 | 1.44E-26 | 0.709727312 | 0.95 | 0.711 | 1.64E-22 | 0 | Epithelial Membrane Protein 3 (EMP3) exhibits high expression levels in samples enriched with retinal pigment epithelium (RPE) (Rickman et al., 2006) and is defined by its four transmembrane domains along with two N-linked glycosylation sites (Ben-Porath et al., 1998). EMP3 is integral to various cellular functions, such as cell proliferation, interactions between cells, and the regulation of differentiation (Lei et al., 2013). Additionally, it is involved in the activation of the caspase-dependent apoptosis pathway (Wang et al., 2017).  Beyond its roles in standard cellular activities, EMP3 has been associated with cancer biology. It undergoes hypermethylation that leads to transcriptional silencing in glioma and neuroblastoma, particularly within the 19q13 chromosome region, where it was initially identified as a tumor suppressor gene. Furthermore, EMP3 may affect the activation of transforming growth factor-beta (TGF-β) (Taylor et al., 1996; Zhang et al., 2022). |
| SQSTM1 | 2.47E-15 | 0.678837092 | 0.595 | 0.333 | 2.81E-11 | 0 | Sequestosome-1/p62 (SQSTM1) plays a crucial role in regulating various cellular functions, including inflammation, apoptosis, and autophagy. It acts as a connector between the processes of autophagy and proteasomal degradation, thereby integrating lysosomal and proteasomal pathways of catabolism (Kolosova et al., 2018). Within the retinal pigment epithelium (RPE), SQSTM1 facilitates autophagy and boosts the Nrf2-mediated antioxidant response, thereby providing protection against acute oxidative stress (Fan et al., 2018).  Recent developments have led to the creation of a DNA plasmid that encodes p62-SQSTM1 (p62DNA), which is being explored as a therapeutic agent for conditions linked to chronic inflammation. This strategy capitalizes on p62's function in regulating oxidative stress and inflammatory responses (Wang et al., 2016). |
| P2RY12 | 2.80E-62 | 0.671927441 | 0.602 | 0.387 | 3.19E-58 | 0 | The purinergic receptor P2RY12 plays a crucial role in the response of microglia to central nervous system (CNS) injuries by detecting ATP and ADP released from damaged cells (Haynes et al., 2006). Additionally, purinergic signaling may influence the colonization of the CNS by microglia, although a specific receptor associated with this process has yet to be identified (Casano et al., 2016). It is believed that P2RY12 assists microglia in recognizing cells that are undergoing normal developmental apoptosis, thereby facilitating their removal. This receptor allows microglia to sense nucleotides, which may create a chemoattractant gradient that aids in the localization of apoptotic cells within the developing retina (Blume et al., 2020). |
| SALL1 | 6.45E-59 | 0.651959433 | 0.679 | 0.477 | 7.35E-55 | 0 | The Sall1 gene encodes a zinc finger transcription factor that was first recognized for its high expression levels in multipotent renal progenitor cells (Nishinakamura et al., 2001). Furthermore, it is specifically expressed in microglia in a healthy central nervous system (CNS) (Lavin et al., 2014). In the developing retina, Sall1 plays a vital role in the regulation of microglial morphology. Moreover, Sall1 is also expressed in astrocytes located in the retina (Koso et al., 2016). |
| TANC2 | 9.18E-49 | 0.650313709 | 0.55 | 0.362 | 1.05E-44 | 0 | Tetratricopeptide repeat, ankyrin repeat, and coiled-coil domain-containing protein 2 (Tanc2) functions as a scaffolding protein that plays a crucial role in the morphogenesis of dendritic spines. It acts as a stable postsynaptic structure, facilitating the recruitment of KIF1A-driven dense core vesicles to these spines. Tanc2 is expressed in various regions, including the hindbrain, neural rod, olfactory placode, pectoral fin, and retina, and is present at both synaptic and non-synaptic locations within neurons. At synaptic sites, Tanc2 engages with PSD-95, enhancing the synaptic capture of vesicles transported by motor proteins (Kim et al., 2021). |
| PPM1G | 2.63E-20 | 0.647894279 | 0.664 | 0.379 | 2.99E-16 | 0 | Mg²⁺/Mn²⁺-dependent protein phosphatase 1 gamma (PPM1G) is a multifunctional enzyme that plays significant roles in a variety of cellular functions. It is vital for the formation of the spliceosome, which is essential for mRNA splicing (Murray et al., 1999). PPM1G also aids in the localization of the survival motor neuron (SMN) complex to Cajal bodies (Petri et al., 2007). Moreover, this enzyme enhances gene-specific transcription (Gudipaty et al., 2015) and modulates protein synthesis by inhibiting cap-dependent translation mediated by 4E-BP1 (Liu et al.). It is crucial for the cell cycle, particularly in managing the transition from the G1 phase to the S phase (Sun et al., 2016). PPM1G is also involved in the dynamic exchange of histones H2A-H2B, which is essential for chromatin remodeling (Kimura et al., 2006). Additionally, it plays a role in the cellular response to DNA damage (Beli et al., 2012) and supports both cell survival and neural development (Foster et al., 2013). |
| CYB5A | 3.81E-22 | 0.646926781 | 0.579 | 0.278 | 4.34E-18 | 0 | Cytochrome b5 is a hemoprotein associated with membranes, anticipated to facilitate both enzyme and heme binding activities. It plays a significant role in the metabolism of fatty acids and acts as an electron carrier for multiple membrane-associated oxygenases. Found within the endoplasmic reticulum, cytochrome b5 is a member of the cytochrome b5 family and is essential for promoting electron transfer in various enzymatic processes. (Makhina et al., 2009). |
| CALR | 9.23E-67 | 0.642691646 | 0.891 | 0.687 | 1.05E-62 | 0 | Calreticulin (CALR) is a calcium-binding chaperone that plays a vital role in protein folding, oligomeric assembly, and quality control within the endoplasmic reticulum (ER), operating through the calreticulin/calnexin cycle. This lectin transiently associates with nearly all monoglucosylated glycoproteins produced in the ER (Pesaresi et al., 2018). Typically, calreticulin resides in the ER; however, it relocates to the cell surface when the ER experiences stress (Lee et al., 2021). At the cell surface, calreticulin functions as a danger-associated molecular pattern (DAMP) and acts as a recognition signal for myeloid cells. This function is particularly important in the context of immune-mediated programmed cell removal (PrCR), where calreticulin on the surface of rod cells aids in the identification and elimination of stressed cells (Lee et al., 2021; Kryslo et al., 2018). The presence of calreticulin on stressed or apoptotic cells enhances their phagocytosis by macrophages and microglia, serving as an "eat-me" signal (Gardai et al., 2005; Martins et al., 2010). Moreover, calreticulin, along with galectin-3, plays a role in innate immune responses. These proteins function as opsonins released by activated microglia to improve bacterial clearance from the brain, indicating a broader involvement of calreticulin in both phagocytic and immune regulatory mechanisms (Cockrom et al., 2019). |
| CTSS | 8.20E-108 | 0.641872747 | 0.974 | 0.852 | 9.35E-104 | 0 | Cathepsin S (CTSS) is a gene that encodes a lysosomal protease and is situated on chromosome 1q21 in humans. It is primarily expressed in various immune cells, such as B cells, dendritic cells (DCs), and macrophages (Lecaille et al., 2002). Lecaille, F., Kaleta, J. and Brömme, D., 2002. Human and parasitic papain-like cysteine proteases: their role in physiology and pathology and recent developments in inhibitor design. Chemical reviews, 102(12), pp.4459-4488.Research has indicated that CTSS is linked to the progression of several diseases, including autoimmune disorders, inflammatory conditions, neurological diseases, and various cancers (Smyth et al., 2022). With respect to expression patterns, CTSS is predominantly found in immune cells including professional antigen-presenting cells (APCs) (Hsing and Rudensky, 2005) , B-cells (Kirschke et al., 1989;), dendritic cells (Kramer et al., 2017) and macrophages (Turk et al., 2012). |
| GCLC | 9.08E-21 | 0.641070024 | 0.247 | 0.076 | 1.03E-16 | 0 | Glutamate-cysteine ligase, catalytic subunit (GCLC), is crucial for facilitating the activity of glutamate-cysteine ligase and is indispensable for the synthesis of glutathione. This enzyme is involved in multiple biological functions, such as inhibiting apoptotic signaling pathways and modulating cellular protein metabolism. GCLC is situated in the cytosol and is a component of the glutamate-cysteine ligase complex. It is found in various tissues, including the digestive system, central nervous system, integumentary system, and sensory organs. Significantly, GCLC exhibits high expression levels in the developing eye and contributes to epithelial cell proliferation (Zhong et al., 2013). |
| BBC3 | 1.41E-33 | 0.631359679 | 0.399 | 0.244 | 1.60E-29 | 0 | BCL2 binding component 3 (BBC3), also referred to as PUMA, serves as a direct activator of BAX, which is essential for the process of developmental cell death in retinal ganglion cells (RGCs), photoreceptors, and certain retinal interneurons (Hahn et al., 2003; Ren et al., 2010). BBC3 is present in the developing retina and is crucial for the occurrence of neuronal death during development, especially following events such as cytokine withdrawal or the inhibition of neuronal activity (Léveillé et al., 2010). A lack of Bbc3 leads to a notable increase in the population of various retinal cell types, including RGCs, underscoring the importance of BBC3 in facilitating BAX-dependent developmental cell death. The regulation of BBC3 is a key determinant in the decision-making process of whether a cell undergoes death in the developing retina and brain. Furthermore, synaptic activity has been shown to inhibit Bbc3 expression in a manner that does not depend on Trp53, indicating that electrical activity may contribute to the survival of RGCs during developmental stages (Harder and Libby et al., 2011). |
| GAPDH | 4.93E-100 | 0.629829024 | 1 | 0.97 | 5.62E-96 | 0 | Glyceraldehyde-3-phosphate dehydrogenase (GAPDH) expression is influenced by the maturation of tissues (Rocha-Martins et al., 2012). Its movement to the nucleus is a significant event in the process of glucose-induced apoptosis in retinal Müller cells (Kanwar and Kowluru, 2009). Furthermore, conditions of low oxygen and the presence of reactive oxygen species lead to the stabilization of hypoxia-inducible factor 1-alpha (HIF-1α), which in turn enhances the expression of GAPDH and other metabolic genes (Solberg et al., 2010).  A stress-responsive signaling pathway known as the N-GAPDH cascade is specifically activated in microglia, facilitating cognitive inflexibility through interactions between microglia and neurons. Microglia, which serve as the brain's resident macrophages, have been identified as sources of extracellular GAPDH. Studies have shown that P2X7 receptors (P2X7R) mediate the ATP-induced release of GAPDH from microglial cells that have been primed with lipopolysaccharide (LPS). This released GAPDH may play a role in the regulation of neuroinflammation and neuritogenesis (Takenouchi et al., 2015).  Exogenous GAPDH has been demonstrated to influence LPS-induced activation of p38 MAPK in MG6 cells, indicating that extracellular GAPDH is involved in modulating microglial activation. This research underscores the potential involvement of GAPDH in neuroinflammatory processes linked to the innate immune response (Takenouchi et al., 2015). Additionally, GAPDH may impact stress-related signaling pathways via cell-surface receptors in microglial cells. While cell adhesion molecules such as L1 and E-cadherin are recognized as potential GAPDH receptors in other cell types, it is still uncertain whether these proteins are present in microglial cells (Makhina et al., 2009). |
| TGFBR1 | 6.82E-51 | 0.617147771 | 0.764 | 0.593 | 7.78E-47 | 0 | Transforming growth factor beta (TGF-beta) plays a crucial role in regulating various cellular processes, including proliferation, migration, differentiation, and apoptosis, by converting extracellular signals into alterations in gene expression. Within the retina, microglia are one of the few cell types that exhibit high levels of TGFBR1 expression. TGF-beta functions as an anti-inflammatory cytokine by activating TGF-beta receptors I (TGFBR1) and II (TGFBR2), which in turn influence the expression of specific target genes (Wrana et al., 1994). The introduction of exogenous TGF-beta has been shown to suppress the production of inflammatory cytokines such as TNF and IL6 by microglia (Taylor et al., 2017). TGFBR1 is involved in the regulation of apoptosis through the ubiquitination of TRAF6 and MAP3K7, and it also influences the epithelial-to-mesenchymal transition via a SMAD-independent mechanism that includes the phosphorylation of PARD6A (Wang et al., 2020). Genes that are specific to microglia include P2ry12, Fcrls, Tmem119, Olfml3, Hexb, and Tgfbr1, with PU.1 recognized as a transcription factor unique to microglia. Additionally, microRNAs such as miR-125b-5p, miR-342-3p, and miR-99a are implicated in the functional regulation of microglia (Butovsky et al., 2014). |
| MT2A | 1.98E-55 | 0.607716845 | 0.414 | 0.169 | 2.25E-51 | 0 | Metallothioneins (MTs) are proteins characterized by a high concentration of cysteine residues, which enable them to bind various heavy metals. Their expression is regulated by heavy metals and glucocorticoids, and they play a vital role in maintaining cellular zinc ion balance, as well as mitigating excitotoxicity, oxidative stress, and inflammation. MTs are significant for the proliferation and survival of cells, particularly in Müller glia (MGs) located in the peripheral retina (Chiha et al., 2018). Elevated levels of metallothioneins and glutathione peroxidase 3 (GPX3) in peripheral MGs may be associated with the aging gradient observed from the fovea to the peripheral retina in humans (Yi et al., 2021).  Vascular endothelial growth factor (VEGF) is a recognized contributor to abnormal retinal angiogenesis in conditions such as age-related macular degeneration (AMD) and diabetic retinopathy. Aging has been linked to an increased expression of VEGF-A in horizontal cells (HCs), MGs, and astrocytes (Yi et al., 2021). This upregulation is associated with degeneration of the retinal pigment epithelium (RPE) and an increased vulnerability to oxidative stress (Wang et al., 2020).  In the cornea, lens, and retina, including RPE tissues, MTs are primarily localized within the cytoplasm of epithelial and endothelial cells of the cornea (Álvarez-Barrios et al., 2021). There exists a complex interaction between microglial activation and the expression of MTs in astrocytes. Within the central nervous system, MT1 and MT2 are predominantly expressed by astrocytes, although their expression has also been detected in neurons, endothelial cells, and microglia (Smit et al., 2022). |
| HSPA5 | 9.46E-59 | 0.601711289 | 0.99 | 0.911 | 1.08E-54 | 0 | HSPA5, commonly referred to as GRP78 or BiP, is a well-recognized chaperone located in the endoplasmic reticulum (ER) that is essential for numerous cellular functions. It plays a pivotal role in the translocation of newly formed polypeptides through the ER membrane, aids in the proper folding and assembly of proteins, directs misfolded proteins towards ER-associated degradation, maintains calcium balance, and acts as a sensor for ER stress. Importantly, extracellular HSPA5 has been identified as a specific marker for cone photoreceptors in mouse retinas (Chintalapudi et al., 2016).  The induction of HSPA5 under stress conditions is a crucial pro-survival element of the unfolded protein response (UPR). This pathway is often activated in neurodegenerative diseases such as Parkinson's disease, Alzheimer's disease, and progressive retinal degeneration, which are marked by UPR activation and changes in HSPA5 expression (Yang et al., 2007) (Hoozemans et al., 2012). However, the precise role of extracellular HSPA5 in the context of photoreceptor degeneration remains to be fully elucidated. |
| PSMA7 | 7.29E-27 | 0.594308927 | 0.954 | 0.821 | 8.31E-23 | 0 | Proteasome subunit alpha 7 (PSMA7) is anticipated to facilitate the binding of identical proteins and plays a role in the catabolism of proteins within the proteasome. It is situated at the postsynapse and constitutes a component of the proteasome core complex. PSMA7 acts as a negative regulator of MAVS-mediated innate immune responses, likely functioning to diminish the onset of an antiviral state during viral infections. This underscores the biological importance of the interaction between PSMA7 and MAVS as a crucial mechanism of cellular regulation (Jia et al., 2009). |
| FAM162A | 4.25E-45 | 0.588507683 | 0.594 | 0.351 | 4.84E-41 | 0 | hFAM162A, also referred to as C3orf28, may play a significant role in the hypoxia-induced death of neuronal cells, potentially by facilitating the release of apoptosis-inducing factor mitochondrion 1 (AIFM1) from the mitochondria into the cytoplasm, followed by its movement to the nucleus. The precise mechanism by which hFAM162A contributes to apoptosis may differ among various cell types and tissues (Cho et al., 2007).  In particular, hFAM162A is thought to influence apoptosis through several pathways, including the release of cytochrome c, activation of caspases, and the induction of mitochondrial permeability transition. In neuronal cells, hFAM162A may enhance the release of AIFM1 from mitochondria into the cytoplasm, which subsequently leads to its translocation to the nucleus. Nonetheless, the involvement of caspases in this process remains a topic of debate in the existing literature.  Additionally, hFAM162A is known to interact with heat shock protein 90 alpha family class B member 1 (HSP90AB), which is essential for the mitochondrial localization of hFAM162A and its pro-apoptotic functions (Cho et al., 2007). |
| SHTN1 | 1.01E-32 | 0.588482655 | 0.355 | 0.1 | 1.15E-28 | 0 | SHTN-1, or Shootin-1, is recognized as a significant contributor to cell migration and neuronal polarization, which are essential processes in the development of the nervous system. It acts as a factor that influences neuronal polarization, facilitating the differentiation of axons by encouraging asymmetric growth of neurites. The regulation of SHTN-1 levels is critical for ensuring appropriate neuronal differentiation and patterning (Emerson et al., 2020). Furthermore, SHTN-1 plays a role in the early stages of axon formation in neurons and is regulated by the RNA-binding protein Ptpb2 (Liang et al., 2021). |
| WNK1 | 8.28E-59 | 0.58248998 | 0.854 | 0.73 | 9.44E-55 | 0 | WNK1 is a serine/threonine protein kinase that is activated through phosphorylation and regulated by at least three distinct promoter regions, facilitating its tissue-specific expression. The expression of WNK1 is further enhanced by alternative splicing and polyadenylation. This kinase is crucial for neural development, influencing processes such as cell proliferation, migration, and differentiation. WNK1 is part of a family of four multifunctional kinases, notable for its unique catalytic lysine position. It phosphorylates key substrates, including oxidative stress-responsive 1 (OSR1) and SPAK (STK39), which are vital for the maintenance of cellular ion balance (Jaykumar et al., 2022).  In the context of embryonic development, WNK1 plays a significant role in sprouting angiogenesis and regulates the expression of endothelial metalloproteinases MMP-2 and MMP-9. It enhances the transcription and secretion of angiogenic factors, indicating its involvement in orchestrating autocrine proangiogenic responses. Additionally, WNK1 is implicated in the regulation of blood pressure and ion transport across cell membranes. Mechanistically, it acts as a chloride sensor, where the binding of chloride ions inhibits its autophosphorylation (Jaykumar et al., 2022).  WNK1 also interacts with components of the TGF-β/SMAD signaling pathway, including AXL, RhoA, ALK1, SMAD2/3, VE-cadherin, and occludin, thereby regulating critical processes in angiogenesis, such as the turnover of cell junctions, in a context-dependent manner. This interaction underscores WNK1's role in modulating TGF-β signaling in response to cellular states, including osmotic homeostasis and mechanical tension (Jaykumar et al., 2022). |
| PDLIM4 | 4.01E-20 | 0.572450903 | 0.564 | 0.273 | 4.57E-16 | 0 | PDZ and LIM domain protein 4 (PDLIM4), commonly referred to as RIL, is essential for the functioning of the central nervous system (CNS) as it facilitates the transport of GluR-A-containing AMPA receptors to dendritic spines through a mechanism that relies on alpha-actinin and actin. This mechanism enhances the transport and/or recycling of these receptors to the postsynaptic membrane (Rios et al., 2020). In nonmuscle cells, PDLIM proteins play a role in stabilizing actin stress fibers. In particular, PDLIM4 interacts with RIL, which increases the binding affinity of α-actinin-1 for filamentous actin (F-actin), resulting in significant reorganization of the actin cytoskeleton (Guryanova et al., 2011). |
| PLCG2 | 3.74E-29 | 0.57032465 | 0.823 | 0.707 | 4.26E-25 | 0 | Phospholipase C gamma 2 (PLCG2) engages with several critical proteins within the microglial phagocytosis pathway, such as HCK, LYN, PIK3R1, and SYK. These proteins play significant roles in various biological processes, including oxidative stress, angiogenesis, and platelet activation (Eaksmunski et al., 2019). PLCG2 is predominantly expressed in microglia and neurons, functioning as a downstream effector of CSF1R, where it undergoes activation via phosphorylation (Obha et al., 2015). This phosphorylation event initiates a swift release of Ca2+ in monocytes, facilitating their differentiation. Furthermore, PLCG2 activates the cAMP-response element-binding protein through a Ca2+ signaling pathway that is mediated by protein kinase A (PKA) (Iwamoto et al., 2005). It plays a role in reducing reactive oxygen species (ROS) levels and is associated with oxidative stress (Berger et al., 2019). Additionally, PLCG2 modulates mTOR activity through the diacylglycerol/protein kinase C signaling pathway, thereby affecting B cell proliferation and apoptosis (Yu et al., 2017). |
| PSMD7 | 3.69E-17 | 0.567102949 | 0.726 | 0.455 | 4.20E-13 | 0 |  |
| MEF2A | 2.13E-59 | 0.548697799 | 0.886 | 0.751 | 2.43E-55 | 0 | MEF2A, originally recognized as a transcription factor specific to muscle tissue, is now understood to be expressed throughout various tissues and to play a role in different tissue phenotypes. In retinal ganglion cells (RGCs), the expression of MEF2A has been associated with the post-natal degeneration of photoreceptor cells and subsequent vision impairment. The role of MEF2A in the survival of RGCs remains debated; some research indicates it may have a neuroprotective effect, while other studies suggest that its overexpression could lead to increased RGC mortality. This implies that MEF2A might facilitate cell death by inhibiting pro-survival genes or by activating pro-apoptotic genes (Xia et al., 2020). |
| IVNS1ABP | 5.49E-27 | 0.538133937 | 0.509 | 0.393 | 6.26E-23 | 0 | NS1BP plays a crucial role in various cellular functions, such as RNA splicing, the negative regulation of protein ubiquitination, and the cellular response to viral infections. It enhances the stability of actin filaments through its Kelch repeat domains, thereby safeguarding cells from apoptosis induced by actin destabilization. Additionally, NS1BP influences the aryl hydrocarbon receptor (AHR) pathway by promoting the availability of AHR for transcriptional activation (Dunham et al., 2006). Furthermore, it serves as a negative regulator of the BCR (KLHL20) E3 ubiquitin ligase complex, inhibiting the ubiquitin-mediated degradation of the tumor suppressors PML and DAPK1 (Chen et al., 2015).). |
| ATF3 | 2.19E-41 | 0.535229103 | 0.932 | 0.8 | 2.50E-37 | 0 | ATF3 is a transcription factor that responds to stress and is typically not found in most healthy neurons; however, its expression is triggered following axonal injury (Hunt et al., 2004). Within the peripheral nervous system, ATF3 has been linked to neuroprotective and regenerative functions. It reduces hypoxia-induced neuronal apoptosis by inhibiting the pro-apoptotic factor carboxyl-terminal modulator protein (CTMP) through its interaction with the ATF/CREB binding site in the CTMP promoter (Huang et al., 2015). Additionally, ATF3 decreases the expression of acetylcholinesterase (AChE), an apoptotic promoter, during stress, particularly in the inner nuclear layer (INL) of the retina, where it serves a protective function (Kole et al., 2020). The expression of ATF3 is selectively regulated and can differ across various cells and tissues, influencing differentiation and regeneration processes. In the retina, ATF3 is notably upregulated in the INL, indicating a specific role in safeguarding this layer by downregulating AChE expression (Heinrich et al., 2018). |
| ADAMTS1 | 2.22E-12 | 0.534690167 | 0.34 | 0.164 | 2.53E-08 | 0 | ADAMTS1, which belongs to the family of disintegrins and metalloproteinases, serves as a significant inhibitor of angiogenesis. It effectively hinders endothelial cell proliferation in vitro, diminishes fibroblast growth factor-2 (FGF-2)-induced vascularization in the corneal pocket assay, and obstructs VEGF-induced angiogenesis in the chorioallantoic membrane assay (Xu et al., 2006).  ADAMTS1 interacts directly with VEGF, inhibiting VEGFR2 phosphorylation and consequently reducing endothelial cell proliferation. Studies utilizing the murine oxygen-induced retinopathy model have demonstrated that VEGF stimulates the expression of ADAMTS1 in endothelial cells via the PKC signaling pathway. Acting as a negative feedback regulator of endothelial cells and angiogenesis, ADAMTS1 represents a promising molecular target for therapeutic intervention in vasculopathic disorders (Xu et al., 2006). |
| MANF | 1.16E-38 | 0.533325382 | 0.666 | 0.423 | 1.32E-34 | 0 | Mesencephalic Astrocyte-derived Neurotrophic Factor (MANF) is a highly conserved immune modulator that is essential for the repair of retinal tissue. As a neurotrophic factor, MANF not only provides direct neuroprotective benefits but also facilitates the alternative activation of innate immune cells, which is crucial for its role in tissue repair (Neves et al., 2016). In the retina, MANF is prominently expressed in retinal ganglion cells (RGCs) and in the inner nuclear layer during the initial two weeks of postnatal development; however, its expression diminishes with age and is absent in the outer nuclear layer (Neves et al., 2016).  Studies have shown that MANF is upregulated by Platelet-Derived Growth Factor (PDGF)-like signaling in injured retinas, where it contributes to neuroprotection, tissue repair, and the effectiveness of photoreceptor replacement therapies. Its potential as a systemic regulator of immune homeostasis indicates that it may address challenges related to age-associated regenerative therapies. Furthermore, MANF is present in microglia/macrophages and other cells within the central nervous system, suggesting that its positive effects may be linked to its influence on microglial polarization and macrophage differentiation (Yang et al., 2020). |
| DOCK10 | 1.95E-30 | 0.532149682 | 0.394 | 0.257 | 2.22E-26 | 0 | DOCK10 is a protein primarily found in leukocytes, although it is also present in certain non-hematopoietic cells. This protein has the capability to activate small GTPases such as Rac and Cdc42, which subsequently promotes the formation of filopodia and membrane ruffles. DOCK10 plays a significant role in the functional regulation of B and T cells, particularly in the processes of B cell differentiation and maturation (Chen et al., 2021). Its expression in the brain, spleen, and thymus indicates a possible involvement in neuroinflammatory responses. Additionally, DOCK10 is upregulated in B cells when stimulated by interleukin-4 and is implicated in the activity of microglia during inflammatory conditions (Namekata et al., 2020). |
| HERC2 | 2.57E-34 | 0.531148714 | 0.596 | 0.468 | 2.93E-30 | 0 | HERC2 (HECT and RLD domain-containing E3 ubiquitin protein ligase 2) participates in various cellular functions, although its complete role remains unclear. Recent studies suggest that HERC2 is responsible for the degradation of crucial DNA damage response proteins, including XPA and BRCA1, via the ubiquitin-proteasome pathway. Additionally, it is involved in regulating the cell cycle, facilitating spindle formation during mitosis, and contributing to mitochondrial energy metabolism. HERC2 also interacts with proteins that regulate the centrosome and the cell cycle, such as PCM1 and CEP170, and modifies these proteins through ubiquitination for targeted signaling activities (Elpidorou et al., 2021). |
| JMJD1C | 5.10E-36 | 0.528015814 | 0.774 | 0.653 | 5.82E-32 | 0 | JMJD1C is a gene that exhibits a range of functions in different cellular environments. Its high expression in rod photoreceptors underscores its importance in visual processes (Bonelli et al., 2021). Moreover, JMJD1C plays a role in the modification of histone marks, which are essential for the regulation of epigenetic mechanisms (Luz-Madrigal et al., 2020). The gene is integral to the DNA damage response pathway, where it demethylates the mediator of DNA damage checkpoint 1 (MDC1) protein, thereby facilitating cellular repair mechanisms (Luz-Madrigal et al., 2020). In terms of neuronal function, JMJD1C is vital for the proper functioning of dendrites (Sáez et al., 2016).  In addition, JMJD1C is significant in megakaryopoiesis and the functioning of platelets. It is linked to platelet counts and aggregation, attributed to its interaction with Ran, a protein that plays a critical role in the regulation of the cytoskeletal network and platelet aggregation (Wang et al., 2022). Furthermore, JMJD1C is found in neuronal populations that express androgen receptors, with its expression levels fluctuating based on hormonal conditions in a region-specific manner (Cloos et al., 2008). |
| JUNB | 3.19E-72 | 0.521098935 | 0.987 | 0.86 | 3.63E-68 | 0 | VEGFA induces JunB expression through a PKC-dependent mechanism, which is crucial for mediating HRMVEC migration, sprouting, and tube formation. Additionally, hypoxia triggers JunB expression in the retina; knocking down JunB specifically in endothelial cells impairs hypoxia-induced endothelial cell proliferation, tip cell formation, and neovascularization (Kumar et al., 2020). In retinal ganglion cells (RGCs), the transcription factors JUN and DDIT3 are upregulated in response to axonal damage. Both of these factors are recognized for their roles in promoting neuronal death. Their activation serves to integrate upstream signaling mechanisms that are initiated by glaucomatous axonal injury, resulting in the expression of pro-apoptotic genes and ultimately leading to RGC apoptosis. JUN specifically regulates pro-death targets, including BIM, ATF3, and HRK (Syc-Mazurek et al., 2017). This knowledge regarding the roles of JUN and DDIT3 in RGC degeneration enhances our understanding of the molecular signaling pathways that contribute to neurodegenerative processes. |
| GPR84 | 2.41E-32 | 0.517618104 | 0.416 | 0.226 | 2.75E-28 | 0 | The increase of GPR84-positive microglial cells in the retina correlates with the death of retinal ganglion cells (RGCs), indicating their potential role in RGC loss following optic nerve injury. These microglial cells tend to adopt a more rounded morphology, which may amplify their inflammatory response. GPR84, belonging to the seven transmembrane receptor superfamily, is significantly upregulated in brain microglia during endotoxemic conditions (Bouchard et al., 2007). It functions as a pro-inflammatory receptor, with medium chain fatty acids serving as its endogenous ligands (Suzuki et al., 2013). Additionally, GPR84 promotes phagocytosis (Recio et al., 2018) and interacts with the adaptor protein DOK3 to facilitate inflammatory processes (Gao et al., 2021). Through single-cell RNA sequencing, distinct clusters of GPR84-positive retinal microglia have been identified, with these cells being classified into various states, including resident, rapid-response, and ameboid, although their specific characteristics remain inadequately characterized (Karlstetter et al., 2015). |
| BTG1 | 5.87E-50 | 0.512709986 | 0.908 | 0.818 | 6.69E-46 | 0 | The B-cell translocation 1 gene (Btg1) is integral to the development of endothelial cells, as it is vital for both the preservation and formation of progenitor cells and new neurons (Farioli-Vecchioli et al., 2012). Btg1 acts as an inhibitory gene in the cell cycle, and its function can be enhanced through the inhibition of phosphatidylinositol 3-kinase (PI3K) (Yi et al., 2021). The PI3K/Akt signaling pathway plays a significant role in the migration, proliferation, and formation of blood vessels in endothelial cells, while PI3K also influences the activity of vascular endothelial growth factor (VEGF). VEGF serves as a key regulator of ocular angiogenesis in diabetic retinopathy (DR), affecting processes such as tube formation, as well as the migration and proliferation of vascular endothelial cells (Zhang et al., 2019) |
| CTTNBP2NL | 2.11E-31 | 0.506785429 | 0.644 | 0.536 | 2.40E-27 | 0 | Cortactin-binding protein 2 N-terminal-like protein (CTTNBP2NL) interacts with cortactin and is associated with stress fibers. Additionally, in HEK293 cells, CTTNBP2NL associates with the striatin-protein phosphatase 2A (PP2A) protein complex (Goudreault et al., 2009) |
| SKIL | 2.79E-39 | 0.505745086 | 0.765 | 0.62 | 3.18E-35 | 0 | The human SKI-like (SKIL) gene is responsible for encoding the SMAD transcriptional corepressor known as SNON, which serves to inhibit TGF-β signaling (Tecalco-Cruz et al., 2012). SNON functions as a negative regulator of SMAD transcription factors and is involved in modulating various effects of TGF-β (Tecalco-Cruz et al., 2012). Variations in SNON expression are associated with several physiological processes, such as embryonic development, liver regeneration, and muscle differentiation. Additionally, changes in SNON levels have been linked to diseases like fibrosis and cancer, which are characterized by disrupted TGF-β signaling (Tecalco-Cruz et al., 2012). As a proto-oncogene, SKIL, also referred to as SnoN, plays a role in the TGF-β signaling pathway and demonstrates pro-oncogenic properties by suppressing the TGF-β/Smad pathway (Sun et al., 1999). |
| HSP90B1 | 3.05E-59 | 0.504561228 | 0.994 | 0.932 | 3.48E-55 | 0 | The HSP90B1 gene is responsible for encoding heat shock protein 108 (HSP108), a protein that is induced by stress and plays a crucial role in the development of the nervous system, especially within the retina. HSP108 participates in a variety of stress responses, which encompass developmental processes, cell cycle regulation, ischemic conditions, inflammation, and responses to infections (Nasaré et al., 2015).  HSP108 operates as a component of the endoplasmic reticulum chaperone complex, facilitating protein folding, mediating ubiquitin-dependent endoplasmic reticulum-associated degradation (ERAD), and managing calcium storage. It collaborates with endoplasmic reticulum proteins to support their proper folding and subsequent degradation (Audouard and Christians et al., 2011).  In the context of neuroinflammation, the role of HSP108 is multifaceted. Although microglial responses to heat shock generally provide neuroprotection, excessive activation of microglia can result in the overproduction of pro-inflammatory factors, leading to neurotoxicity (Swaroop et al., 2016). A study examining the pro-inflammatory response of microglia and the impact of the HSP90 inhibitor ganetespib found that levels of HSP90α and HSP90β mRNA and protein increased following heat shock. The findings indicated that ganetespib significantly diminished pro-inflammatory responses by inhibiting HSP90α, highlighting its potential therapeutic implications for controlling dysregulated inflammatory responses (He et al., 2019). |
| PF4V1 | 0 | 5.839711471 | 0.949 | 0.015 | 0 | 1 | Platelet Factor 4 (PF4), also referred to as CXC chemokine platelet factor-4 (PF-4/CXCL4), is a protein encoded by the PF4-Like gene, which is crucial for antiangiogenesis and tumor suppression. As a primary component of platelet granules, PF4 is released during platelet activation. It was the first chemokine discovered to inhibit neovascularization (El-Asar et al., 2015). PF4 has been shown to obstruct VEGF-induced signaling and migration in human retinal microvascular endothelial cells (HRMEC) (El-Asar et al., 2015). However, despite its angiostatic capabilities, the levels of PF4 may not always be adequate to mitigate the neovascular growth and advancement of proliferative diabetic retinopathy (PDR) (El-Asar et al., 2015).  PF4's anti-angiogenic properties are realized through its direct binding to and antagonism of pro-angiogenic factors such as VEGF, thereby preventing VEGF from engaging with its receptors. Furthermore, PF4 competes with VEGF for binding sites on proteoglycan receptors, such as heparin or sulfate heparin, or it can displace VEGF from these sites, thus inhibiting its function (Cai et al., 2020). Studies also suggest that PF4 and its splicing variants possess anti-tumor properties by hindering inflammatory angiogenesis and enhancing immune responses for tumor detection and suppression (Cai et al., 2020). |
| MRC1 | 0 | 4.096104028 | 0.895 | 0.012 | 0 | 1 | Mannose receptor, C type 1 (Mrc1) is essential for the regulation of replisome progression, functioning independently of DNA damage. Cells lacking Mrc1 (mrc1?) exhibit a reduced rate of progression through the S phase, whereas cells with specific mutations (mrc117AQ) are able to maintain normal S phase progression. In contrast, cells with C-terminal truncations of Mrc1, despite showing nearly normal kinetics of checkpoint activation, also demonstrate a slow progression through the S phase (Osborn and Elledge, 2003).  Mrc1, in conjunction with the associated proteins Csm3 and Tof1 (which are similar to Tipin/Timeless in human cells), has been shown to enhance replication fork rates in vitro (Yeeles et al., 2017). It interacts with replication forks during the S phase and engages with various components of the replisome (Baretić et al., 2020; Lou et al., 2008). Nevertheless, the precise mechanism by which Mrc1 regulates fork progression is not yet fully understood. Recent research suggests that the phosphorylation of Mrc1 by Rad53 inhibits its ability to promote replication, indicating that Mrc1 serves a dual role as both a mediator and a target within the checkpoint pathway (McClure and Diffley, 2021). |
| DAB2 | 0 | 3.595019446 | 0.884 | 0.096 | 0 | 1 | Disabled-2 (Dab2) is integral to the cellular trafficking of membrane receptors and the processes of signaling transduction. It plays a pivotal role in regulating Transforming Growth Factor-β (TGF-β) signaling by influencing the internalization of the TGF-β receptor (Finkielstein and Capelluto, 2016). Research conducted by Nakayama et al. demonstrated that Dab2, in conjunction with PAR-3, is vital for the activation of VEGF-VEGFR-2 signaling through the regulation of VEGFR-2 internalization in retinal microvascular endothelial cells. This finding indicates that Dab2 is significantly involved in angiogenesis (Cheong et al., 2006). Furthermore, both Dab2 and PAR-3 have been identified as essential factors in the modulation of VEGFR-2 internalization within retinal vascular endothelial cells (Jing et al., 2020). |
| F13A1 | 0 | 2.418820774 | 0.669 | 0.001 | 0 | 1 | Factor XIII (FXIII) plays a pivotal role in the blood coagulation process and has attracted significant interest due to its involvement in angiogenesis and tissue repair. FXIII facilitates wound healing by cross-linking newly generated fibrin fibers and enhancing platelet adhesion to the injured sub-endothelium, thereby contributing to a stable tissue structure. Furthermore, FXIII aids in angiogenesis through its interactions with vascular endothelial growth factor receptor 2 (VEGFR2) and integrin αVβ3, both of which are essential for the formation of granulation tissue and effective healing (Gemmati et al., 2016). Nonetheless, these mechanisms can also result in excessive neovascularization in conditions such as inflammatory bowel disease and retinal degenerative disorders. FXIII functions as a protransglutaminase in the final phase of blood coagulation, where it establishes cross-links between the γ-glutamyl and ε-lysine residues of fibrin chains (Wang et al., 2011). |
| LYVE1 | 0 | 2.359587161 | 0.581 | 0.001 | 0 | 1 | Lymphatic-vessel endothelial protein LYVE-1 serves as a receptor for the glycosaminoglycan polymer known as hyaluronan (HA), which is defined by the structure [GlcNAcβ1-4GlcUAα1-3]n. This protein is predominantly found at the unique overlapping junctions of initial lymphatics, while it is largely absent from the tight junctions of larger collector vessels. LYVE-1 shares a close relationship with CD44, a key receptor on leukocytes that binds HA and facilitates the adhesion of leukocytes and their movement across inflamed blood vessels. The role of LYVE-1 in leukocyte trafficking is significant, as it enhances the attachment of dendritic cells (DCs) to the endothelium of lymphatic vessels through interactions with HA present on DCs. The specific expression of this receptor in lymphatic tissues, particularly at the tips of interdigitating endothelial junctions in initial lymphatic vessels, aids in the transmigration of DCs, likely through mechanisms involving both direct adhesion and the disruption of local endothelial junctions (Johnson et al., 2017). |
| CITED2 | 4.86E-114 | 2.219583043 | 0.932 | 0.579 | 5.54E-110 | 1 | Cited2 plays a significant role during the development of the eye and is essential for proper eye formation. Its absence leads to abnormalities in eye development, resulting in conditions such as lens-corneal fusion and excessive hyaloid vascularization. Cited2 is known to interact with HIF-1 signaling, which is critical for the tissue's response to low oxygen levels (Bhattacharya et al., 1999). In hypoxic conditions, the HIF-1α subunit accumulates and, in conjunction with HIF-1β, initiates the transcription of target genes like VEGF by binding to hypoxia-response elements, aided by p300 (Freedman et al., 2003). As a gene that is induced by hypoxia, Cited2 influences HIF-1 signaling by binding to p300 with greater affinity than HIF-1α, thus diminishing hypoxia-driven transcription. Furthermore, Cited2 operates either upstream of or in conjunction with Pax6, a crucial transcription factor involved in lens morphogenesis (Pugh and Ratcliffe, 2003). The genetic interplay between Cited2 and HIF-1 is essential for the development of both the lens and the hyaloid vasculature (Chen et al., 2008). |
| SNX2 | 2.72E-127 | 2.15109782 | 0.904 | 0.634 | 3.09E-123 | 1 | Sorting nexins, particularly the retromer-associated SNX-BAR proteins, consist of heterodimeric pairs formed by SNX1 or SNX2 in combination with SNX5 or SNX6 (Horazdovsky et al., 1997). These proteins are essential for the regulation of tubular endosomal sorting profiles. They facilitate the development of specific tubular structures and tubulovesicular transport carriers (van Weering et al., 2012). SNX-BAR proteins are instrumental in the spatial and biochemical organization of these entities, thereby affecting the efficacy of endosomal sorting and transport processes (Simonetti et al., 2017). |
| BMP2 | 0 | 2.087209756 | 0.456 | 0.001 | 0 | 1 | Bone morphogenetic proteins (BMPs) are a group of cytokines classified within the transforming growth factor-β superfamily, first recognized for their ability to promote the formation of bone and cartilage (Nasrabadi et al., 2018). In addition to their original function, BMPs are now acknowledged for their diverse roles in the regulation of angiogenesis, tissue homeostasis, and tumor development, with BMP signaling activation noted in various pathological conditions (Kwon et al., 2014; Morrell et al., 2016). Specifically, BMP2 plays a crucial role in the development of multiple organs, such as the lungs, heart, and central nervous system (Lliu et al., 2009). Nonetheless, BMP2 is also implicated in pathological processes associated with vascular inflammation and angiogenesis (Helbing et al., 2011). The BMP endothelial cell precursor-derived regulator (BMPER), recognized as a negative regulator of BMP signaling, has been demonstrated to alleviate vascular inflammation and preserve normal retinal vascular homeostasis by inhibiting BMP signaling (Al-Shabrawi et al., 2021). |
| BLVRB | 2.43E-155 | 2.076993774 | 0.969 | 0.665 | 2.77E-151 | 1 | Biliverdin reductase B (BLVRB), commonly referred to as flavin reductase (FLR), is an NADPH-dependent oxidoreductase characterized by its extensive substrate specificity. It has been identified as a key regulator of cellular redox homeostasis (Cunningham et al., 2000). BLVRB functions as a general flavin reductase, capable of reducing a diverse array of substrates, which implies its significant involvement in redox regulation (Shalloe et al., 1996). This role is particularly important in erythropoiesis, as evidenced by the increased levels of BLVRB mRNA, indicating its high presence in mature red blood cells (RBCs) where it contributes to the management of cellular redox states (We et al., 2016). The expression of BLVRB is predominantly found in cells with elevated heme synthesis, such as developing erythroid cells and both fetal and adult liver tissues. It demonstrates NADPH-dependent reductase activity towards non-alpha isomers of biliverdin, flavins, and redox cycling agents. The knockdown of BLVRB in induced MEL cells leads to heightened intracellular oxidation and increased cytotoxicity during oxidative stress and heme synthesis inhibition, underscoring its essential role in preserving cell viability under such conditions (Lok et al., 2019). Therefore, while BLVRB/FLR is primarily recognized for its function in biliverdin reduction, it is also crucial for cytoprotection in developing erythroid cells, especially in the context of oxidative stress and fluctuating heme levels (Paukovich et al., 2018). |
| MAF | 2.57E-162 | 2.069607414 | 0.992 | 0.82 | 2.92E-158 | 1 | A basic-region leucine zipper (bZIP) transcription factor, MAF, particularly the small MAF subfamily (sMAFs), forms heterodimers with Nrf2 to bind Maf-recognition elements (MAREs) in response to oxidative stress. In M¸ller cells, a key cellular response to strong light (a stress condition) involves an increase in Nrf2 and sMAF levels compared to dim light (an unstressed condition) (Chen et al., 2022). Nrf2 plays a crucial role in stress defense for M¸ller cells, which support retinal ganglion cells (RGCs). In a co-culture model, Nrf2 knockout results in reduced RGC numbers and increased RGC death, impacting visual acuity (Navneet et al., 2019). A novel Nrf2 activator, RS9, has been shown to protect M¸ller cells from light-induced stress (Inoue et al., 2017). This highlights the importance of Nrf2 in retinal stress response and suggests that targeting Nrf2 could be a promising therapeutic approach for degenerative retinal diseases like AMD and DR.  Additionally, a new macrophage/microglia activation factor (MAF) was identified, which is expressed by microglial cells upon activation. This MAF, a membrane protein with seven putative transmembrane domains, is associated with late endosomes/lysosomes and its expression increases following lesions. MAF is involved in the dynamics of late endosomal/lysosomal membranes in microglial cells and may contribute to secondary neuronal degeneration after brain lesions. Future studies using gene silencing techniques are needed to further explore MAF's role in microglial-neuronal interactions (Zhang et al., 2023). |
| CTSC | 1.03E-146 | 1.982856252 | 0.745 | 0.21 | 1.18E-142 | 1 | Cathepsin C, also referred to as dipeptidyl peptidase I (DPPI), is a significant component of the peptidase C1 family and functions as a lysosomal cysteine proteinase. It plays a crucial role in the activation of numerous serine proteinases within immune and inflammatory cells. DPPI acts as a pivotal coordinator for the activation of serine proteases that are vital for an organism's defense mechanisms. This protease is particularly involved in the activation of pro-inflammatory serine proteases in various cell types, including cytotoxic T-lymphocytes, natural killer cells, neutrophils, and mast cells (Turk et al., 2001).  As a tetrameric cysteinyl protease, DPPI is essential for the activation of different serine proteases that contribute to inflammatory responses. It is constitutively released from neutrophils, and its activity can be influenced by the surrounding extracellular environment. Gaining insight into the factors that regulate DPPI expression and the impact of DPPI inhibitors may have therapeutic relevance, especially in the modulation of elastase activity in mature blood neutrophils (Zhou et al., 2009). |
| KLF4 | 5.48E-49 | 1.936388866 | 0.569 | 0.268 | 6.25E-45 | 1 | Krüppel-like factor 4 (Klf4) serves as a vital transcriptional regulator that plays a significant role in the processes of stem cell differentiation and reprogramming (Wei et al., 2013). In rodents, Klf4 is expressed in the developing retinal ganglion cells (RGCs) (Moore et al., 2009) and exhibits a dual functionality. On one hand, Klf4 promotes the self-renewal and pluripotency of stem cells; on the other hand, it acts to inhibit axon growth and regeneration in RGCs following injury (Jiang et al., 2008). This dual role aligns with Klf4's established function in suppressing neurite growth (Rocha-Martins et al., 2019). |
| LRP6 | 7.40E-139 | 1.808549865 | 0.697 | 0.19 | 8.44E-135 | 1 | Low-density lipoprotein receptor-like protein 6 (LRP6) is essential for the stabilization of cytosolic catenin, as demonstrated by He et al. (2004). In the retina, most cell types express both LRP5 and LRP6 (Chen et al., 2007), leading to an incomplete understanding of their distinct functions in Wnt signaling mediation. It is noted that the majority of Wnt ligands activate Wnt signaling primarily through LRP6, indicating its significant role in this pathway (Singh et al., 2021). As a co-receptor in Wnt/β-catenin signaling, LRP6 is crucial for regulating various processes such as cell proliferation, specification, migration, and the maintenance of stem cell homeostasis (MacDonald et al., 2012). Furthermore, LRP6 is vital for the activation of Wnt/β-catenin signaling, which influences numerous cellular activities, including cell growth and tissue stability. Additionally, LRP6 is involved in embryonic development through various biological mechanisms across multiple species, including Xenopus laevis, chickens, mice, and humans (Alrefaei and Abu-Elmagd, 2022). |
| FOSB | 2.53E-86 | 1.800661177 | 0.768 | 0.358 | 2.89E-82 | 1 | The d Fos gene family consists of "early response" genes that are activated in various tissues and cell types in response to external stimuli, leading to alterations in cell growth and differentiation pathways (Kim et al., 2010). These genes produce leucine zipper proteins that pair with JUN family proteins to create the transcription factor complex known as AP-1. This complex plays a crucial role in regulating gene expression in reaction to various stimuli, including cytokines, growth factors, stress, and infections (Hess et al., 2004). Notably, the FOSB gene encodes leucine zipper proteins that interact with JUN proteins to form AP-1, thereby impacting processes such as cell proliferation, differentiation, and transformation (Tang et al., 2016). |
| MARCKSL1 | 1.22E-107 | 1.79731925 | 0.853 | 0.419 | 1.39E-103 | 1 | Myristoylated Alanine-Rich C-kinase Substrate (MARCKS) and its homolog MARCKS-like protein 1 (MARCKSL1) serve diverse functions, including contributions to embryonic development, adult brain plasticity, and the inflammatory response. Research indicates that MARCKSL1 facilitates the formation of lamellipodia, promotes axon outgrowth, and enhances cell motility in both neurons and cancer cells (Björkblom et al., 2012; Laux et al., 2000; Yu et al., 2015). This protein is upregulated in the central nervous system (CNS) and peripheral nervous system (PNS) across all vertebrate species, and it is also expressed in various mesodermal and endodermal tissues (Ott et al., 2011; Shi et al., 1997; Zolessi et al., 2001; Ruiz-Perera et al., 2013). Additionally, MARCKSL1 plays a role in the adhesion and migration of neural crest cells (NCCs) and is involved in regulating migration during the development of numerous other tissues. In the immune system, MARCKSL1 is crucial for the migration of inflammatory cells and the secretion of cytokines, and it is associated with macrophage transmigration through mechanisms that include phosphorylation, actin binding, and cytosolic translocation (Green et al., 2012). |
| SNX6 | 3.81E-157 | 1.794663339 | 0.994 | 0.91 | 4.34E-153 | 1 | Sorting nexin 6 (SNX6) belongs to the sorting nexin (SNX) family and plays a significant role in retrograde vesicular transport mediated by retromer and driven by dynein-dynactin, facilitating the movement of vesicles from endosomes to the trans-Golgi network (TGN). This process is essential for the sorting and trafficking of endosomal proteins (Niu et al., 2017). Additionally, SNX6 has been associated with the regulation of various proteins, such as the transforming growth factor β (TGF-β) receptors (Parks et al., 2001), the oncogene Pim-1 (Ishibashi et al., 2001), and the translationally controlled tumor protein TCTP (Yoon et al., 2006). |
| MTSS1 | 1.16E-160 | 1.77317199 | 0.654 | 0.13 | 1.33E-156 | 1 | Metastasis Suppressor 1 (MTSS1), also referred to as MIM, belongs to the inverse-Bin/Amphiphysin/Rvs (I-BAR) domain superfamily and is essential for neuronal development and functionality. During the developmental phase, MTSS1 is temporarily expressed in granule cells, marking the transition from their proliferation to their integration into synapses. Additionally, it is found in various embryonic tissues, particularly within the developing central nervous system (Glassmann et al., 2007).  From a functional perspective, MTSS1 is instrumental in initiating dendritic protrusions in neurons by linking I-BAR-mediated plasma membrane deformation with ARP2/3-driven actin polymerization. It serves a dual role as both an activator of ARP2/3 and an inhibitor of formins, thereby affecting the actin nucleation pathways that are vital for the morphogenesis of neuronal dendrites (Galbraith et al., 2018). |
| EHD1 | 2.59E-226 | 1.767671328 | 0.581 | 0.056 | 2.95E-222 | 1 | Eps15 homology domain-containing (EHD) proteins represent a newly recognized family of regulatory proteins involved in endocytic recycling, playing significant roles in various cellular functions. Notably, EHD1 is found in endocytic recycling compartments and is vital for the recycling of transferrin and the retrograde transport of proteins to the trans-Golgi network (Grant and Caplan, 2008; George et al., 2007). EHD1 facilitates the recycling of multiple cell surface receptors, including the transferrin receptor, MHC-I and MHC-II molecules, the GLUT4 glucose transporter, and β1-integrin (Lin et al., 2001; Walseng et al., 2008; Scheiblin et al., 2014; Guilherme et al., 2004).  Additionally, EHD1 serves as a crucial regulator in the development of the lens in mice, influencing embryonic viability, growth, ocular development, and fertility. In the later stages of postnatal eye development, EHD1 expression is detected in the ganglion cell layer as well as in the outer and inner nuclear layers of the neural retina (Rapaport et al., 2006). In the lens epithelium, EHD1 targets important cell surface receptors such as IGF1-R and β1-integrin, along with potential targets like FGF and BMP receptors, which are essential for the survival of lens epithelial and fiber cells (Jovic et al., 2007). |
| TXNIP | 4.54E-111 | 1.749865585 | 0.66 | 0.201 | 5.18E-107 | 1 | Thioredoxin-interacting protein (TXNIP) is a protein associated with pro-oxidative stress, inflammation, and apoptosis, which is significantly upregulated in response to diabetes and elevated glucose levels in various tissues, including pancreatic beta cells and retinal cells (Singh et al., 2013). TXNIP exerts its effects by binding to thioredoxin (Trx), thereby inhibiting its thiol-reducing and oxidant-scavenging functions, which results in increased oxidative stress and apoptosis within the cell (Yoshihara et al., 2014). Although TXNIP is predominantly found in the cytosol and nucleus, it relocates to the mitochondria under conditions of cellular stress, where it initiates cell death signaling by releasing apoptosis-signal kinase 1 from its association with Trx2 (Saxena et al., 2010). Research has demonstrated that the upregulation of TXNIP in the retina, triggered by diabetes or high glucose levels, contributes to oxidative stress, inflammation, and subsequent apoptosis (Perrone et al., 2009; Devi et al., 2012; Santos et al., 2011). |
| PRDX11 | 6.54E-35 | 1.55089813 | 0.896 | 0.841 | 7.45E-31 | 2 | Peroxiredoxins, predominantly found in neurons of the inner nuclear layer and cone photoreceptors, are essential for the detoxification of hydrogen peroxide, alkyl hydroperoxides, and peroxynitrite in living cells. Notably, cells in the inner nuclear layer that express Prdx1 were consistently negative for markers associated with Müller cells, indicating that they are indeed neurons. This observation, combined with their specific location, likely designates them as bipolar cells (Chidlow et al., 2016). |
| H2AFZ3 | 3.03E-31 | 2.12033964 | 1 | 0.971 | 3.46E-27 | 2 | The gene involved in chromatin organization is connected to the facilitation of higher-order chromatin structures (Hao et al., 2012). |
| STMN12 | 7.64E-29 | 1.998399843 | 1 | 0.52 | 8.70E-25 | 2 | Stathmin 1 (STMN1) is a cytoplasmic phosphoprotein that plays a crucial role in the regulation of microtubule dynamics within cells and is significant in facilitating processes such as cell differentiation, proliferation, and migration. STMN1 affects a range of cellular activities, including DNA methylation, m6A modifications, the infiltration of immune cells, and the modulation of immunological molecules and immune checkpoints. Enrichment analyses utilizing Gene Ontology (GO) and Kyoto Encyclopedia of Genes and Genomes (KEGG) pathways have indicated that STMN1, along with its associated hub genes, participates in the regulation of the mitotic cell cycle, microtubule organization, gap junctions, the FoxO signaling pathway, and apoptosis (Zhang et al., 2022). |
| TUBB4B1 | 1.54E-24 | 1.891118067 | 0.941 | 0.527 | 1.76E-20 | 2 | Tubulin 4B is an integral part of microtubules, which are formed from polymers of α- and β-tubulin heterodimers. These microtubules are vital for numerous cellular functions, such as cell division, the transport of proteins within cells, the formation of cilia, and the operational activities of cilia. Additionally, they are critical for the construction of centrioles and axonemes in cells with multiple cilia (Luscan et al., 2017). |
| CENPF2 | 2.94E-111 | 1.791651404 | 0.686 | 0.036 | 3.35E-107 | 2 | CENPF is a gene that regulates microtubules (MT) and is vital for the development of centrioles, which are necessary for the formation of centrosomes and cilia. Alterations in centrosomal and MT-regulating genes, such as CENPF, have been connected to various cancers and disorders related to neuronal migration. CENPF has a role in interacting with cytoplasmic dynein-1 and in recruiting the spindle checkpoint regulatory complex to the spindle poles during the late stages of anaphase. Furthermore, mutations in CENPF have been associated with human ciliopathy and microcephaly phenotypes (Waters et al., 2015). |
| TUBB32 | 4.20E-40 | 1.714940653 | 0.725 | 0.128 | 4.79E-36 | 2 | III-Tubulin, also known as class III β-Tubulin, is a neural-specific variant within the tubulin family that contributes to the formation of microtubules in the cytoskeleton. This protein serves as a distinguishing marker for neurons, allowing for the differentiation between neurons and glial cells, which lack β-III-Tubulin expression. Notably, retinal ganglion cells (RGCs) exhibit high levels of β-III-Tubulin due to their neuronal lineage. This protein is specifically present in neurons, particularly in early-born retinal neurons, including RGCs, thereby positioning it as a potential marker for these cells. It has been employed as a marker for RGCs in various experimental models of optic nerve injury, such as optic nerve crush or transection (Jiang et al., 2015).. |
| HIST1H2BN1 | 2.09E-22 | 1.710662774 | 0.686 | 0.19 | 2.38E-18 | 2 |  |
| CCNB2 | 3.47E-129 | 1.685308995 | 0.667 | 0.029 | 3.96E-125 | 2 | Cyclin B2 belongs to the B-type cyclins, which also encompass Cyclin B1. Both Cyclin B1 and B2 interact with p34cdc2 and play a vital role in the regulation of the cell cycle. Cyclin B1 is mainly found in association with microtubules, whereas Cyclin B2 is linked to the Golgi apparatus. Additionally, Cyclin B2 has been shown to bind with transforming growth factor beta RII, indicating that the Cyclin B2/cdc2 complex may be significant in modulating cell cycle progression in response to transforming growth factor beta signaling. Cyclin B2 is critical for the regulation of the cell cycle during the G2/M (mitosis) transition (Daldello et al., 2019). |
| HMGB22 | 1.37E-21 | 1.657550199 | 0.98 | 0.67 | 1.57E-17 | 2 | HMGB2 is primarily found in the outer nuclear layer (ONL) and the inner and outer segments of photoreceptors (IS/OS) within the retina. It plays a significant role in photoreceptor pyroptosis by promoting pro-inflammatory responses and oxidative stress during light-induced retinal degeneration (LIRD), primarily through the down-regulation of the Nrf2/HO-1 pathway and the activation of NF-κB/NLRP3 signaling pathways. The silencing of HMGB2 can mitigate these effects. This research underscores the harmful impact of HMGB2 on photoreceptor pyroptosis and suggests its potential as a therapeutic target for retinal degenerative diseases (Zhang et al., 2022).  Beyond its involvement in retinal disorders, HMGB2 also participates in the organization and remodeling of chromatin structure. It is expressed in both immortalized human and mouse cell lines, which may help in addressing limitations related to replication. Notably, HMGB2 (as opposed to HMGB1) has been demonstrated to inhibit malignant cell proliferation in vivo by modulating the gene expression pathways associated with hypertrophic cell growth (Starkova et al., 2023). |
| CKS21 | 2.12E-18 | 1.603296794 | 0.843 | 0.407 | 2.42E-14 | 2 | Cyclin-dependent kinase subunit 2 (CKS2) plays a vital role in the cell cycle by interacting with the catalytic subunit of cyclin-dependent kinases (Daniszewski et al., 2022). Its function is essential for the process of cell division, and the inhibition of CKS2 can lead to a halt in the cell cycle during the G2 phase. Additionally, CKS2 is linked to neuronal differentiation and the regulation of the cell cycle (Feng et al., 2022). |
| CDCA81 | 2.56E-67 | 1.569841773 | 0.588 | 0.047 | 2.91E-63 | 2 | Cell Division Cycle Associated 8 (CDCA8) serves as a vital regulator of the mitotic process. It is instrumental in the localization of the centromere protein complex (CPC) to the centromere, rectifying errors in kinetochore attachment, and ensuring the stability of bipolar spindles. Notably, the transcriptional activity of CDCA8 is significantly elevated in embryos and embryonic stem cells (Shuai et al., 2021). |
| LGALS12 | 5.11E-16 | 1.336815998 | 0.538 | 0.382 | 5.83E-12 | 2 | Galectin-1, encoded by the LGALS1 gene, regulates cell growth, migration, and proliferation by binding to various receptors depending on their glycosylation profile. It plays a critical role in angiogenesis through lectin-dependent binding to N-glycans of VEGF receptor 2 (VEGFR2), thereby activating downstream signaling in vascular endothelial cells as a VEGFR2 ligand (Yamamoto et al., 2021). Galectin-1 is significantly upregulated in retinal vascular endothelial and Müller glial cells under hypoxic conditions. This upregulation is driven by hypoxia-responsive elements (HREs) in the LGALS1 promoter region. Galectin-1 also regulates apoptosis, cell proliferation, and differentiation, and inhibits CD45 protein phosphatase activity, preventing dephosphorylation of Lyn kinase, which makes it a strong inducer of T-cell apoptosis (Yamamoto et al., 2021). |
| FTH11 | 9.93E-22 | 1.293479507 | 1 | 0.996 | 1.13E-17 | 2 | This gene encodes the heavy subunit of ferritin, a key intracellular iron storage protein in both prokaryotes and eukaryotes. Ferritin consists of 24 subunits of heavy and light chains, with the composition affecting iron uptake and release in various tissues. Its main function is to store iron in a soluble, non-toxic form. Ferritin plays a critical role in maintaining iron homeostasis, making iron available for cellular processes while protecting lipids, DNA, and proteins from iron's toxic effects. Defects in ferritin are linked to neurodegenerative diseases. In the retina, ferritin is primarily expressed in the inner segments of photoreceptors, the RPE, choroid, inner nuclear layer, and ganglion cell layer (Gnana‐Prakasam et al., 2010). In mouse retina, ferroportin is found in the RPE, photoreceptor inner segments, inner and outer plexiform layers, and the ganglion cell layer (Gnana‐Prakasam et al., 2010). |
| LGALS31 | 3.44E-10 | 1.255724585 | 0.321 | 0.19 | 3.92E-06 | 2 | Lectin, galactoside-binding, soluble (Gal-3), which is encoded by the LGALS3 gene, has multifaceted roles in both inflammation and neuroinflammation. In the context of neuroinflammatory diseases like multiple sclerosis, Gal-3 is typically upregulated and can facilitate both pro-inflammatory and anti-inflammatory responses. Notably, a deficiency in Gal-3 correlates with a milder form of autoimmune encephalomyelitis (Jiang et al., 2009). In the retinal environment, Gal-3 is absent in healthy mice but shows increased expression in the retinal endothelium during experimental autoimmune uveitis (EAU), although its specific pathogenic role in EAU has yet to be established (Lipski et al., 2020). Regarding ischemic brain injury, Gal-3 seems to exhibit a dual function: it may act as a pro-inflammatory agent in the early stages while providing protective effects later during degenerative processes (Rahimian et al., 2018). Likewise, in cases of traumatic brain injury, Gal-3 demonstrates both neuroprotective and neurodegenerative properties, which are influenced by the severity and progression of the injury (Nishikawa and Suzuki, 2018). Recent research indicates that Gal-3 may have a pro-inflammatory effect in the retina, as evidenced by diminished neuroinflammation and enhanced axon myelination in Gal-3 knockout mice subjected to diabetes (Mendonça et al., 2018). Within the central nervous system (CNS), Gal-3 is essential for the activation of microglia and is associated with the pathology of Alzheimer's disease (AD) (Puigdellívol et al., 2020). It is primarily expressed by microglia and astrocytes during neuroinflammatory states (Song et al., 2014). Gal-3 can modulate microglial activation, potentially promoting the M1 pro-inflammatory phenotype through the action of IFN-γ and the JAK/STAT signaling pathway (Jeon et al., 2010). As an endogenous ligand for TLR4, Gal-3 interacts with microglial TLR4, thereby maintaining M1 pro-inflammatory responses during acute neuroinflammation (Burgillos et al., 2015). |
| FTL1 | 6.85E-29 | 1.249311921 | 1 | 0.989 | 7.80E-25 | 2 | Ferritin, composed of heavy (FTH1) and light (FTL) chains, plays a vital role in maintaining iron balance within the body. The heavy chain, FTH1, is essential for iron binding and mitigating oxidative damage in the retina. The degeneration of Müller glia cells results in the accumulation of iron in the retinal tissue (Menon et al., 2019).  The light chain, FTL, is predominantly found in amacrine and ganglion cells (Greferath et al., 2009). Ferritin is structured from 24 subunits of both heavy and light chains, allowing it to store iron in a soluble and non-toxic manner, which is critical for iron homeostasis. Alterations in the composition of ferritin subunits can influence the processes of iron uptake and release. Mutations in the FTL gene have been associated with neurodegenerative disorders and hyperferritinemia-cataract syndrome (Greferath et al., 2015).  In the inner retina, FTL expression frequently corresponds with regions exhibiting abnormal Müller cell activity. FTL has been utilized as a marker for investigating neural circuits that respond to various stimuli, such as water regulation, learning and memory, and respiratory control. Additionally, it acts as a reporter for c-fos, a gene that is upregulated during instances of cell death, neural activity, and plasticity (Greferath et al., 2015). |
| GCLM1 | 0.008553 | 1.22034494 | 0.277 | 0.247 | 1 | 2 | Glutamate-cysteine ligase (GCL) serves as a pivotal enzyme in the synthesis of glutathione (GSH), consisting of a catalytic subunit (GCLC) and a modifier subunit (GCLM). Although GCLM does not possess enzymatic activity, it enhances the enzyme's affinity for glutamate and ATP, which in turn increases the concentration of GSH necessary for the inhibition of GCL (McConnachie et al., 2007). GSH is essential for the detoxification of cellular free radicals and provides neuroprotective effects within the brain. A reduction in brain GSH levels is linked to a variety of neurological disorders. While genetic defects in enzymes related to GSH are uncommon, disorders in GSH metabolism are frequently observed in neurodegenerative diseases (Sanchez-Vallejo et al., 2016). GCL functions as the rate-limiting enzyme in the GSH biosynthetic pathway, and the presence of the modifier subunit GCLM enhances the enzyme's ability to synthesize GSH, thereby offering protection against oxidative stress (Yang et al., 2002). |
| ESD1 | 2.04E-08 | 0.930232753 | 0.723 | 0.779 | 0.00023269 | 2 | Esterase activity is crucial for the early development of Müller cells and ganglion cells within the retina. In the initial differentiation phase, Müller cells, which arise at the vitreal border, demonstrate elevated β-esterase activity. As these cells migrate outward, there is a gradual decline in this enzymatic activity (Bhattacharjee and Sanyal, 1975a). Ganglion cells initially exhibit β-esterase activity, which is soon followed by the emergence of acetylcholinesterase (AChE) activity. As these cells increase in size, their enzyme activity escalates rapidly, with larger ganglion cells displaying significantly higher levels of these enzymes. This early developmental phase also witnesses the emergence of giant and displaced ganglion cells, which bear resemblance to those found in the retinas of various species (Polyak, 1941; Shkolink-Yarros, 1971; Anctil and Ali, 1974).  The development of horizontal cells is similarly characterized by the activity of β-esterase and AChE. As the cell bodies expand and position themselves along the inner side of the developing outer plexiform layer, the activity of these enzymes also rises. An increase in β-esterase activity in Müller cells is associated with heightened carbonic anhydrase activity in both the outer and inner plexiform layers, indicating the growth of lateral processes (Bhattacharjee, 1976). The outer plexiform layer, where synaptic connections form earlier than in the inner plexiform layer (Olney, 1968; Weidman and Kuwabara, 1968), underscores the significance of these developmental changes in enzyme activity within the retina (Bhattacharjee and Sanyal, 1975b). |
| BLVRB2 | 4.01E-08 | 0.776342544 | 0.695 | 0.692 | 0.00045649 | 2 | Biliverdin reductase B (BLVRB), commonly referred to as flavin reductase (FLR), is an NADPH-dependent oxidoreductase characterized by its extensive substrate specificity. It has been identified as a key regulator of cellular redox homeostasis (Cunningham et al., 2000). BLVRB functions as a general flavin reductase, capable of reducing a diverse array of substrates, which implies its significant involvement in redox regulation (Shalloe et al., 1996). This role is particularly important in erythropoiesis, as evidenced by the increased levels of BLVRB mRNA, indicating its high presence in mature red blood cells (RBCs) where it contributes to the management of cellular redox states (We et al., 2016). The expression of BLVRB is predominantly found in cells with elevated heme synthesis, such as developing erythroid cells and both fetal and adult liver tissues. It demonstrates NADPH-dependent reductase activity towards non-alpha isomers of biliverdin, flavins, and redox cycling agents. The knockdown of BLVRB in induced MEL cells leads to heightened intracellular oxidation and increased cytotoxicity during oxidative stress and heme synthesis inhibition, underscoring its essential role in preserving cell viability under such conditions (Lok et al., 2019). Therefore, while BLVRB/FLR is primarily recognized for its function in biliverdin reduction, it is also crucial for cytoprotection in developing erythroid cells, especially in the context of oxidative stress and fluctuating heme levels (Paukovich et al., 2018). |
| PSMA71 | 1.90E-15 | 0.770203612 | 0.787 | 0.833 | 2.16E-11 | 2 | Proteasome subunit alpha 7 (PSMA7) is anticipated to facilitate the binding of identical proteins and plays a role in the catabolism of proteins within the proteasome. It is situated at the postsynapse and constitutes a component of the proteasome core complex. PSMA7 acts as a negative regulator of MAVS-mediated innate immune responses, likely functioning to diminish the onset of an antiviral state during viral infections. This underscores the biological importance of the interaction between PSMA7 and MAVS as a crucial mechanism of cellular regulation (Jia et al., 2009). |
| ATOX1 | 4.64E-23 | 0.748587726 | 0.835 | 0.844 | 5.29E-19 | 2 | Copper transport protein Antioxidant-1 (Atox1) is primarily known for its role as a copper (Cu) chaperone; however, it has recently been recognized as a Cu-dependent transcription factor that plays a significant role in inflammatory neovascularization. Atox1 is vital for promoting angiogenesis by modulating the activity of the copper-dependent enzyme lysyl oxidase (LOX), which is responsible for altering the extracellular matrix (ECM). Furthermore, Atox1 acts as a transcription factor that increases the production of reactive oxygen species (ROS) and enhances inflammatory responses by upregulating the expression of the NADPH oxidase organizer p47phox. These dual functions underscore the importance of Atox1 in both angiogenic and inflammatory processes (Chen et al., 2024). |
| IGF12 | 1.95E-06 | 0.743868882 | 0.526 | 0.48 | 0.02221724 | 2 | Insulin-like growth factor-I (IGF-1) serves as a crucial ligand for the IGF-1 receptor (IGF-1R), which belongs to the tyrosine kinase receptor superfamily. IGF-1 plays a significant role in regulating normal developmental growth through both endocrine and autocrine/paracrine pathways (Bates et al., 1995). It acts as a powerful survival factor for various tissues, enhancing synaptic plasticity, promoting nerve growth, and initiating antiapoptotic signaling pathways within the central nervous system (CNS) (Heemskerk et al., 1999).  In the context of the retina, IGF-1 is recognized as a strong proangiogenic agent (Labandeira-Garcia et al., 2017). Additionally, it acts as a mitogenic factor for microglia, affecting neuroinflammatory responses by altering the phenotype of microglia and facilitating a change in their functional activity. The influence of IGF-1 on neuroinflammatory responses is dependent on the specific context, and a deficiency in IGF-1 can result in chronic low-grade inflammation, which may contribute to retinal degeneration associated with aging (Arroba et al., 2018). |
| H2AFZ1 | 6.52E-13 | 0.726329779 | 0.952 | 0.973 | 7.43E-09 | 2 | The protein involved in chromatin organization facilitates the formation of higher-order chromatin structures, which in turn affects gene regulation and chromatin dynamics. This structural organization is essential for various biological processes, including transcription, DNA replication, and repair (Hao et al., 2012). |
| ATP5MC1 | 7.01E-19 | 0.711330558 | 0.839 | 0.838 | 7.99E-15 | 2 | This gene is responsible for encoding a subunit of mitochondrial ATP synthase, an enzyme that facilitates the synthesis of ATP by harnessing the electrochemical gradient of protons across the inner mitochondrial membrane during the process of oxidative phosphorylation. ATP synthase is composed of two interconnected multi-subunit complexes: the soluble catalytic core (F1) and the membrane-embedded component (Fo), which creates the proton channel. Together, these complexes collaborate to promote the generation of ATP, which serves as the main energy currency of the cell (Morciano et al., 2021). |
| PSMB6 | 2.16E-16 | 0.707078028 | 0.867 | 0.903 | 2.46E-12 | 2 | Proteasome 20S subunit beta 6 is a component of the constitutive proteasome involved in the proteolytic degradation of most intracellular proteins. The proteasome complex is crucial for protein homeostasis, working by associating with regulatory particles. The 20S core complex pairs with two 19S regulatory particles to form the 26S proteasome, which facilitates ATP-dependent degradation of ubiquitinated proteins. This process is essential for removing misfolded or damaged proteins and those no longer needed by the cell (Choi et al., 2018).  In the retina, mTORC1 induces the expression of the immunoproteasome component Psmb9 via signal transducer and activator of transcription 1 (Stat1), indicating the importance of Stat1 in activating Psmb9 as part of the cellular response to interferon (Choi et al., 2018). Proteasome subunits, including PSMA6, PSMA7, PSMB6, PSMB7, PSMB9, and PSMD13, are integral to the multicatalytic proteinase structure responsible for degrading intracellular proteins. The proteasome pathway is particularly significant for removing oxidatively damaged proteins, such as those in the lens (Diehn et al., 2005). |
| TMEM246 | 2.76E-17 | 0.697341262 | 0.819 | 0.811 | 3.15E-13 | 2 |  |
| COX7C | 1.93E-28 | 0.691687562 | 0.888 | 0.893 | 2.20E-24 | 2 | Cytochrome c oxidase subunit 7C (Cox7c) mRNA, which encodes a mitochondrial protein integral to complex IV of the respiratory chain, has been investigated for its cotransport with mitochondria. This mRNA is transcribed from the nuclear genome but exhibits a significant association with mitochondria in both neuronal cell lines and primary motor neurons (Cohen et al., 2022). Techniques such as MS2-tagging and live imaging have confirmed this cotransport phenomenon. Prior research has indicated that mRNAs coding for components of the oxidative phosphorylation pathway are frequently enriched in proximity to mitochondria (Williams et al., 2014). Additionally, Cox7c mRNA is particularly abundant in rodent axons (Rotem et al., 2017). |
| NDUFB7 | 5.17E-10 | 0.689717724 | 0.663 | 0.705 | 5.89E-06 | 2 | NADH Dehydrogenase (Ubiquinone) 1 Beta Subcomplex, 9 (ND1β9) is a component of the mitochondrial oxidative phosphorylation complex I. Situated within the inner mitochondrial membrane, ND1β9 is vital for the dehydrogenation process of nicotinamide adenine dinucleotide (NADH). It enables the transfer of electrons from NADH to coenzyme Q (ubiquinone), a critical step in the electron transport chain that is necessary for ATP synthesis via oxidative phosphorylation (Harada et al., 2011). |
| NME2 | 1.44E-34 | 0.68747991 | 0.96 | 0.956 | 1.64E-30 | 2 | NME1, or nucleoside diphosphate kinase 1 (NDPK1), plays a significant role in various cellular functions, such as normal cell growth, differentiation, development, and signal transduction. It is involved in the endocytosis of G protein-coupled receptors and the regulation of gene expression (Meehta et al., 2009; Rochdi et al., 2004). NME1 is essential for neural development, particularly in neural patterning and determining cell fate (Owlani et al., 2012). Its NDPK activity is important for the functioning of dynamin and the contractility of endothelial cells (Boissan et al., 2014). Furthermore, NME1/2 interacts with dynamin to facilitate effective endocytosis (Schlattner et al., 2021). |
| CYCS | 3.95E-05 | 0.677352102 | 0.373 | 0.331 | 0.4501223 | 2 | Cytochrome c is a protein located in the mitochondria that is released into the cytosol upon receiving apoptotic signals. This release triggers the activation of APAF1, which subsequently leads to the formation of the apoptosome and the activation of caspase-9. The release of cytochrome c occurs in two distinct phases: first, it is mobilized from the intermembrane space, followed by its translocation through the outer mitochondrial membrane. The precise mechanism of this release is still a topic of discussion, particularly regarding the roles of the outer versus inner mitochondrial membranes; however, the prevailing evidence suggests that the outer membrane is primarily involved in this process. Furthermore, this release is tightly controlled, mainly by proteins from the BCL2 family (Ow et al., 2012). |
| RPS26 | 2.16E-10 | 0.676731196 | 0.659 | 0.675 | 2.46E-06 | 2 | Ribosomal Protein S26 (RPS26) plays a crucial role in the negative regulation of RNA splicing (Cai et al., 2012). It encodes proteins that constitute the small subunits of ribosomes, which are vital for the process of protein synthesis. RPS26 is consistently present across all subpopulations of retinal pigment epithelium (RPE) and is widely distributed in retinal organoids derived from induced pluripotent stem cells (iPSC) (Cai et al., 2012). |
| COX8A | 2.98E-13 | 0.663953905 | 0.695 | 0.7 | 3.39E-09 | 2 | Cytochrome c oxidase, also known as complex IV, serves as the terminal enzyme in the mitochondrial electron transport chain, playing a vital role in oxidative phosphorylation. This enzyme is part of a complex assembly of multisubunit structures that collaborate to facilitate the transfer of electrons from NADH and succinate to molecular oxygen, thereby establishing an electrochemical gradient across the inner mitochondrial membrane. This gradient is essential for driving ATP synthesis through ATP synthase. The primary function of cytochrome c oxidase is to catalyze the reduction of oxygen to water. Electrons derived from reduced cytochrome c, located in the intermembrane space, are conveyed through the dinuclear copper A center (Cu(A)) found in subunit 2 and heme A in subunit 1. These electrons ultimately arrive at the active site, which is a binuclear center (BNC) consisting of heme A3 and copper B (Cu(B)) in subunit 1. The BNC facilitates the reduction of molecular oxygen to two water molecules by utilizing four electrons from cytochrome c along with four protons sourced from the mitochondrial matrix (Michel et al., 1998). |
| MIF1 | 2.14E-13 | 0.648972694 | 0.916 | 0.889 | 2.44E-09 | 2 | Migration inhibitory factor (MIF) serves a pivotal role in the processes of antigen presentation and immune regulation. Its overexpression is known to worsen ocular inflammation, a phenomenon linked to the activation of the Notch signaling pathway, which is essential for both innate and adaptive immune responses. MIF functions as a proinflammatory cytokine, exerting a considerable effect on macrophage activity and T cell activation (Stoffelns et al., 2010). Moreover, MIF enhances the expression of Toll-like receptor 4 (TLR-4) (Nakazawa et al., 2007).  In addition to its role as a hormone produced by the anterior pituitary and a high-activity enzyme, MIF is widely acknowledged for its contribution to inflammatory and autoimmune disorders (Nishihira, 2012; Hoi et al., 2006). It is also involved in the proliferation and survival of neural stem/progenitor cells (NSPCs) (Ohta et al., 2012). Research conducted by Matsuda et al. has underscored MIF's significance in the regulation of ocular immunity and inflammation (Matsuda et al., 1997). Furthermore, MIF's anti-inflammatory properties can influence the actions of glucocorticoids. It is synthesized by various cell types within the brain, including microglia, astrocytes, and neurons (Nasiri et al., 2020). |
| SEC61G | 2.04E-16 | 0.625295106 | 0.863 | 0.898 | 2.32E-12 | 2 | The SEC61 complex serves as a fundamental element of the protein translocation machinery located within the endoplasmic reticulum (ER) membrane. It is essential for processes such as protein folding, modification, and translocation, and it participates in the unfolded protein response (Liu et al., 2019). Within this complex, SEC61G acts as a membrane transporter in the ER and has the potential to interact with various proteins (Zhang et al., 2022). |
| SEC61B | 2.41E-16 | 0.620819286 | 0.835 | 0.871 | 2.75E-12 | 2 | In situations of excessive stress, the Unfolded Protein Response (UPR) initiates multiple pathways aimed at restoring balance within the endoplasmic reticulum (ER). This response promotes the expression of genes targeted by the UPR, which encode various proteins, including molecular chaperones, folding catalysts, components of the translocation machinery (such as the Sec61 complex), molecules involved in ER-associated degradation (ERAD), and antioxidants. The activation of these pathways improves the capacity for protein folding and degradation while simultaneously reducing protein synthesis, thereby mitigating stress and reestablishing homeostasis in the ER (Ziaka and van der Spuy, 2022). |
| MYO10 | 0.0015 | 0.616855849 | 0.369 | 0.356 | 1 | 2 | Myosin X (Myo10) is a multifunctional motor protein that serves various roles within the cell. It promotes adhesion between filopodia and either other cells or the extracellular matrix (ECM) by interacting with cadherins and β-integrins. Myo10 is involved in several essential biological processes, such as spindle assembly, the formation of endothelial tubes, the transfer of melanosomes (which is associated with filopodia development), axon guidance, cell movement, and phagocytosis. Furthermore, Myo10 contributes to neuritogenesis and the guidance of axons. It may also play a part in Wnt signaling that induces cell death, particularly in the interactions between retinal microglia (a type of macrophage) and hyaloid vessels. Studies indicate that Myo10 is vital for the development of elongated, slender filopodia from vascular endothelial cells, which engage with resident macrophages. Nonetheless, the precise function of Myo10-mediated filopodia formation in the communication between endothelial cells and macrophages, as well as in programmed capillary regression, has yet to be fully understood (Bachg et al., 2019). |
| GAPDH1 | 1.94E-24 | 0.616460569 | 0.992 | 0.977 | 2.21E-20 | 2 | Glyceraldehyde-3-phosphate dehydrogenase (GAPDH) expression is influenced by the maturation of tissues (Rocha-Martins et al., 2012). Its movement to the nucleus is a significant event in the process of glucose-induced apoptosis in retinal Müller cells (Kanwar and Kowluru, 2009). Furthermore, conditions of low oxygen and the presence of reactive oxygen species lead to the stabilization of hypoxia-inducible factor 1-alpha (HIF-1α), which in turn enhances the expression of GAPDH and other metabolic genes (Solberg et al., 2010).  A stress-responsive signaling pathway known as the N-GAPDH cascade is specifically activated in microglia, facilitating cognitive inflexibility through interactions between microglia and neurons. Microglia, which serve as the brain's resident macrophages, have been identified as sources of extracellular GAPDH. Studies have shown that P2X7 receptors (P2X7R) mediate the ATP-induced release of GAPDH from microglial cells that have been primed with lipopolysaccharide (LPS). This released GAPDH may play a role in the regulation of neuroinflammation and neuritogenesis (Takenouchi et al., 2015).  Exogenous GAPDH has been demonstrated to influence LPS-induced activation of p38 MAPK in MG6 cells, indicating that extracellular GAPDH is involved in modulating microglial activation. This research underscores the potential involvement of GAPDH in neuroinflammatory processes linked to the innate immune response (Takenouchi et al., 2015). Additionally, GAPDH may impact stress-related signaling pathways via cell-surface receptors in microglial cells. While cell adhesion molecules such as L1 and E-cadherin are recognized as potential GAPDH receptors in other cell types, it is still uncertain whether these proteins are present in microglial cells (Makhina et al., 2009). |
| RANBP11 | 0.000188 | 0.604732232 | 0.518 | 0.538 | 1 | 2 | The nuclear export of LKB1 is governed by Ran and its effector, RanBP1, both of which play vital roles in numerous cellular functions. In particular, RanBP1 contributes to the polarization of epithelial cells and rat cortical neurons (Mencarelli et al., 2018). It is indispensable for the formation of axons in cultured cortical neurons and facilitates neuronal migration during the development of the cortex in vivo (Mencarelli et al., 2018). Additionally, RanBP1 is essential for the survival of neural crest (NC) cells (Barriga et al., 2022). Ran GTPase, a key regulatory G protein switch, oversees nucleocytoplasmic transport. The active form of Ran GTPase, known as Ran-GTP, exhibits a strong affinity for binding with Ran-binding protein 1 (RanBP1) and Ran-binding protein 2 (Patil et al., 2013). |
| RPL38 | 1.97E-24 | 0.604409694 | 0.916 | 0.96 | 2.24E-20 | 2 | Ribosomal Protein L38 (RPL38) is essential for the assembly of the 80S ribosomal complex on particular mRNAs, serving as a regulatory element that enables transcript-specific control of translation. This role of RPL38 is especially important for the translation of certain Hox genes, which are critical for tissue patterning during developmental processes. Any disruption in RPL38's function can result in defects in tissue patterning, as demonstrated in Ts/+ models. Furthermore, RPL38 exhibits high expression levels in the neural retina, underscoring its significance in both retinal function and development (Kondrashov et al., 2011). |
| REXO2 | 0.00068 | 0.602262743 | 0.438 | 0.444 | 1 | 2 | REXO2 plays a vital role in the turnover of RNA and the preservation of promoter specificity within mammalian mitochondria. This protein is involved in the final phase of mitochondrial RNA (mtRNA) degradation, where it converts RNA into mononucleotides that can be reused for subsequent rounds of transcription. Additionally, REXO2 is significant in embryonic development; research indicates that its conditional deletion results in altered patterns of mitochondrial transcription and highlights a reliance on dinucleotides for the initiation of mitochondrial transcription that is independent of promoters. Nicholls et al. have identified human oligoribonuclease REXO2 as a specialized dinucleotidase, underscoring its critical role in these biological processes (Nicholls et al., 2019). |
| RPL36AL | 4.26E-31 | 0.598663869 | 0.988 | 0.979 | 4.86E-27 | 2 | The gene encoding Ribosomal Protein L36a-like (RPL36AL) is involved in translation and is a component of the 60S ribosomal subunit. This protein is predicted to function upstream of or within the response to retinoic acid, which is important for various cellular processes including differentiation and development. RPL36AL is located in several cellular components, including the cytosol, endoplasmic reticulum, and nucleus, reflecting its diverse roles in cellular function and gene regulation (Milenkovic et al., 2025). |
| NDUFB9 | 1.13E-08 | 0.597420458 | 0.683 | 0.715 | 0.00012833 | 2 | NADH Dehydrogenase (Ubiquinone) 1 Beta Subcomplex, 9 (NDUFB9) serves as a subunit of mitochondrial Complex I, commonly referred to as NADH oxidoreductase. This enzyme complex is situated within the inner mitochondrial membrane and is vital for the process of oxidative phosphorylation, which is necessary for ATP generation. The primary role of NDUFB9 is to facilitate the dehydrogenation of nicotinamide adenine dinucleotide (NADH) and to transfer electrons to coenzyme Q (ubiquinone). This electron transfer represents a fundamental phase in the mitochondrial respiratory chain, aiding in the establishment of a proton gradient that is essential for ATP synthesis (Li et al. 2015). |
| SERF2 | 1.26E-45 | 0.582863316 | 1 | 0.997 | 1.44E-41 | 2 | The small EDRK-rich factor 2 (SERF2) is a highly conserved protein that is involved in the regulation of amyloid fiber formation and the facilitation of protein misfolding in vitro. Its role in age-related proteotoxicity has not been thoroughly investigated, primarily due to the absence of in vivo models (Cleverley et al., 2021). SERF2 displays a sex-specific expression pattern, being found in certain male tissues while absent in female tissues. It is present in the nervous systems of both mice and humans, as well as in endothelial cells. Studies suggest that SERF2 alters the aggregation of amyloidogenic proteins within the intricate biological milieu of the mammalian brain, potentially providing a developmental advantage. Furthermore, SERF2 may affect cell proliferation either directly or by safeguarding cells from stressors that create an adverse environment for cell division. A reduction in SERF2 levels could impede overall growth due to its critical function in sustaining a conducive cellular environment (Stroo et al., 2023). |
| MTRNR2L6 | 0.000449 | 0.57801215 | 0.719 | 0.839 | 1 | 2 | MT-RNR2-like 6, classified as a pseudogene, is believed to exhibit receptor antagonist properties and play a role in the negative regulation of the apoptosis execution phase. It is expected to be situated within the cytoplasm and the extracellular space. Recent studies emphasize the protective function of humanin (MTRNR2), a peptide derived from mitochondria, in safeguarding the retinal pigment epithelium (RPE) against oxidative stress. Both humanin and its 13 nuclear-encoded isoforms (MTRNR2L) are essential for maintaining RPE homeostasis and functionality, which are critical factors in the development of age-related macular degeneration (AMD). Research indicates that humanin expression is markedly elevated in the RPE/choroid compared to the retina in normal donor eyes. Furthermore, significant alterations in the expression of humanin nuclear isoform genes within the RPE have been observed, influenced by oxidative stress and hypoxia as AMD progresses (Zhang et al., 2023). |
| TMSB4X | 8.55E-63 | 0.572111953 | 1 | 0.999 | 9.75E-59 | 2 | Thymosin 4 (T4), which is encoded by the TMSB4X gene, is known to be involved in the cytoplasmic sequestration of NF-κB; however, its specific function in retinal pigment epithelial (RPE) cells remains inadequately explored. The presence of TMSB4X, in conjunction with DCT—an established marker for RPE development—indicates a potential role in both immature RPE populations and the later stages of RPE maturation (Lidgerwood et al., 2021). This gene produces a protein that sequesters actin, which is vital for processes such as actin polymerization, cell proliferation, migration, differentiation, and the formation of dendrites, often in collaboration with CRYBB2. Thymosin proteins play a significant role in a variety of cellular functions, including cell migration, vesicle transport, and the development of cellular morphology. Furthermore, proteins associated with the actin cytoskeleton are critical for the formation of neurons and the morphogenesis of dendrites, with TMSB4X exhibiting considerable activity in regulating actin dynamics at cell ruffles (Sun et al., 2018). |
| TBCA | 0.002289 | 0.569883275 | 0.438 | 0.465 | 1 | 2 | The gene in question encodes a protein that is one of four co-factors (A, D, E, and C) involved in the pathway for folding beta-tubulin. Cofactors A and D are believed to capture and stabilize beta-tubulin intermediates in a quasi-native conformation. This protein functions downstream of the cytosolic chaperonin CCT in the tubulin folding pathway (Tian et al., 1996). Molecular chaperones are essential for the formation of tubulin heterodimers, which cannot form without their assistance ( Yaffe et al., 1992). |
| HINT1 | 6.51E-16 | 0.569140182 | 0.859 | 0.917 | 7.42E-12 | 2 | Histidine triad nucleotide-binding protein 1 (HINT1) is an enzyme that is broadly expressed and is known to hydrolyze lysyl-AMP in vitro. It acts as a tumor suppressor and plays a role in apoptotic pathways. Its expression is particularly high in the mouse sciatic nerve, which correlates with its involvement in disorders of the peripheral nervous system. Mutations that result in the loss of function of HINT1 are a major contributor to hereditary peripheral neuropathy accompanied by neuromyotonia, specifically referred to as autosomal recessive axonal neuropathy with neuromyotonia (ARAN-NM) (Zimoń et al., 2012). |
| ELOB | 2.91E-18 | 0.565992299 | 0.888 | 0.909 | 3.31E-14 | 2 | Elongin-B serves as a subunit within the transcription factor B (SIII) complex, which also comprises elongins A/A2 and C. The SIII complex, characterized by Elongin A as its transcriptionally active element and elongins B and C as regulatory components, is essential for facilitating RNA polymerase II (Pol II) elongation by mitigating transient pauses at various transcriptional sites (Bradsher et al., 1993).  The Elongin BC complex, first recognized as a significant enhancer of Pol II elongation, is also part of the von Hippel-Lindau (VHL) tumor suppressor complex (Duan et al., 1995). This complex exhibits multifunctionality and modulates a range of cellular activities by engaging with a short degenerate sequence motif found in several proteins, including Elongin A, VHL, and SOCS-box family proteins (Aso et al., 1996). The evolutionary preservation of Elongins B and C, along with numerous BC-box proteins, indicates that the regulatory mechanisms involving the Elongin BC complex are likely conserved across different species (Kamura et al., 1998). |
| NAA38 | 0.005791 | 0.564875904 | 0.369 | 0.371 | 1 | 2 | N-alpha-acetyltransferase 38 (NAT38) is an auxiliary subunit of the NatC complex, which is involved in protein N-terminal acetylation. NAT38 plays a role in the negative regulation of apoptosis and is localized in the cytoplasm and nucleoplasm. It colocalizes with polysomes, indicating its involvement in translation. As part of the NatC complex, NAT38 contributes to the regulation of various cellular processes through its acetylation functions (Deng et al., 2023). |
| GTF2F2 | 7.05E-08 | 0.563169339 | 0.643 | 0.678 | 0.00080301 | 2 | Transcription Initiation Factor IIF Subunit Beta 3 (TFII-I) is anticipated to facilitate the activity of RNA polymerase II as a general transcription initiation factor and plays a significant role in transcription mediated by RNA polymerase II. This factor is situated within the microtubule cytoskeleton and the nucleoplasm, contributing to the formation of the transcription preinitiation complex. TFII-I is classified within a diverse family of transcription factors that assimilate signals from various pathways to orchestrate cellular responses to environmental stimuli. Research suggests that TFII-I is essential for the early stages of mouse development, and its structural characteristics allow it to convey multiple regulatory signals, underscoring its function as a versatile transcription factor (Fijalkowska et al., 2010). |
| PSMB5 | 3.05E-07 | 0.561922197 | 0.711 | 0.795 | 0.00347803 | 2 | The beta 5 subunit of the 20S proteasome (PSMB5) is integral to the chymotrypsin-like activity of the proteasome and serves as a recognized marker for autophagy, frequently linked with microtubule-associated protein 1 light chain 3 (LC3) (Eskelinen, 2005). The proteasome is a complex proteinase with a well-defined ring-shaped 20S core, composed of four rings containing 28 distinct subunits: two rings of seven alpha subunits and two rings of seven beta subunits. These proteasomes are abundantly present in eukaryotic cells and operate through an ATP/ubiquitin-dependent mechanism to degrade peptides in a manner that does not involve lysosomes. The immunoproteasome, a variant of the standard proteasome, is essential for the processing of class I MHC peptides. PSMB5, belonging to the B-type family of proteasome subunits, is a beta subunit of the 20S core that is not found in the immunoproteasome, where it is substituted by the catalytic subunit 3i (proteasome beta 8 subunit) (Atkinson et al., 2012). |
| RAN1 | 1.10E-05 | 0.561542742 | 0.582 | 0.616 | 0.12499536 | 2 | RAN (ras-related nuclear protein) is a small GTP-binding protein belonging to the RAS superfamily, essential for the translocation of RNA and proteins across the nuclear pore complex. In addition to its role in macromolecular transport, RAN is involved in the formation and organization of the microtubule network. Importantly, RAN serves as a critical signaling molecule that modulates microtubule polymerization during mitosis and oversees nucleocytoplasmic transport in interphase cells, as well as spindle checkpoint signaling in dividing cells (Patil et al., 2014). |
| NME1 | 0.000274 | 0.558245549 | 0.47 | 0.481 | 1 | 2 | NME1 (nucleoside diphosphate kinase 1) plays a crucial role in various cellular processes, including normal cell proliferation, differentiation, and development. It is also integral to signal transduction, the endocytosis of G protein-coupled receptors, and the regulation of gene expression (Mehta and Orchard, 2009). This protein is vital for neural development, encompassing aspects such as neural patterning and the determination of cell fate (Owlani et al., 2012). Furthermore, the NDPK activity of NME1 is significant for dynamin function and the contractility of endothelial cells (Adam et al., 2020). The interaction between NME1/2 and dynamin is essential for optimizing the efficiency of endocytosis (Schlattner, 2021). |
| ETFB | 5.28E-09 | 0.557335023 | 0.723 | 0.755 | 6.02E-05 | 2 | The beta subunit of electron transfer flavoprotein facilitates the transfer of electrons between primary flavoprotein dehydrogenases that play a role in the catabolism of fatty acids and amino acids within the mitochondria, and the membrane-associated electron transfer flavoprotein ubiquinone oxidoreductase. Mutations or deficiencies in this gene have been linked to type II glutaric aciduria (Merritt and Gallagher, 2018). |
| ATP5MD | 2.75E-05 | 0.547135661 | 0.566 | 0.613 | 0.31300825 | 2 | Mitochondrial ribosomal proteins (MRPs) are produced by genes located in the nucleus, synthesized in the cytoplasm, and then moved to the mitochondria, where they are incorporated into mitochondrial ribosomes. These proteins play a vital role in the process of mitochondrial oxidative phosphorylation (OXPHOS) (Huang et al., 2020). |
| SYNGR1 | 9.68E-14 | 0.54260712 | 0.912 | 0.873 | 1.10E-09 | 2 | Synaptogyrins 1, 2, 3, and 5 are integral membrane proteins that are linked to presynaptic vesicles in neuronal cells. While their precise functions remain uncertain, research on analogous murine proteins indicates that they may contribute to synaptic plasticity, although they are not critical for the process of synaptic transmission. These proteins are implicated in the modulation of both long-term and short-term synaptic plasticity in neurons and are believed to function upstream of or within the cellular mechanisms responding to leukemia inhibitory factor. They are anticipated to reside within the synaptic vesicle and are active at neuromuscular junctions as well as within the synaptic vesicle membrane. Synaptogyrin is found in various anatomical structures, including the adrenal gland, central nervous system, genitourinary system, hemolymphoid system gland, and retina. It is considered orthologous to the human gene SYNGR1 (synaptogyrin 1) (Janz et al., 1990), |
| COPS9 | 3.17E-05 | 0.542166192 | 0.614 | 0.682 | 0.36153994 | 2 | The COP9 signalosome complex (CSN), comprising COPS components, plays a crucial role in various cellular and developmental functions. It is instrumental in regulating processes such as embryonic development, cell cycle progression, DNA damage repair, checkpoint control, signal transduction, circadian rhythms, T-cell maturation, and autophagy (Qin et al., 2020). COPS facilitates these activities by modulating cullin-RING ligases (CRLs), which represent a significant category of E3 ubiquitin ligases that enhance the ubiquitination of regulatory proteins, leading to their degradation via the proteasome (Rao et al., 2020). |
| ATP6V1F | 4.07E-09 | 0.541478239 | 0.739 | 0.789 | 4.63E-05 | 2 | The ATPase H+ Transporting V1 Subunit F, commonly referred to as ATP6V1F, is responsible for encoding a vital component of the vacuolar ATPase (V-ATPase) complex, which plays a key role in the acidification of intracellular organelles in eukaryotic cells. This acidification is essential for a range of intracellular functions, such as protein sorting, activation of zymogens, receptor-mediated endocytosis, and the preservation of the proton gradient within synaptic vesicles. Additionally, the ATP6V1A gene encodes a peripheral subunit of the V1 domain of V-ATPase, which participates in the ATP-binding interface and facilitates the hydrolysis of ATP (Vasanthakumar et al., 2020). |
| CAPG1 | 0.004478 | 0.539402523 | 0.277 | 0.237 | 1 | 2 | CapG, a structural protein associated with chromatin and a component of the condensin I complex, is essential for the regulation of gene expression in post-mitotic neurons. It contributes to the maintenance of the neuronal transcriptome and may play a role in preserving genomic stability without relying on the DNA damage response. CapG seems to exert a direct regulatory influence on the transcription of genes at its binding sites. The reduction of CapG in younger neurons implies that condensin I is vital for the establishment of a mature chromosomal architecture in differentiated neurons prior to synaptogenesis. Furthermore, CapG may be critical for the terminal differentiation of newly generated neurons and for sustaining their cellular identity once they have matured. The elevated levels of CapG in newly formed neurons, in contrast to those in more differentiated neurons, underscore its significant function during the early phases of neuronal development (Hassan et al., 2020). |
| OAZ1 | 1.48E-14 | 0.537933681 | 0.904 | 0.923 | 1.69E-10 | 2 | Ornithine Decarboxylase Antizyme 1 (Oaz1) is a member of the antizyme family and plays a significant role in the regulation of cellular growth and proliferation through the modulation of intracellular polyamine concentrations. It is essential for the progression of the cell cycle, primarily by inhibiting ornithine decarboxylase (ODC), the principal enzyme responsible for polyamine synthesis. The expression of Oaz1 is elevated in early postnatal retinal progenitor cells (RPCs), which is associated with the extended cell cycle duration observed in late-stage RPCs (Clark et al., 2019). |
| OST4 | 9.28E-09 | 0.533146747 | 0.791 | 0.854 | 0.00010579 | 2 | Oligosaccharyltransferase (OST) is a protein complex integrated within the membrane that facilitates the N-glycosylation of newly synthesized polypeptides within the endoplasmic reticulum (ER) lumen. This highly conserved biosynthetic mechanism plays a vital role in enhancing the structure and functionality of proteins. OST4, as a part of this complex, is believed to play a direct role in the catalysis of protein N-glycosylation (Dumax-Vorzet et al., 2013). |
| TBCB | 9.30E-06 | 0.529981974 | 0.574 | 0.609 | 0.10603933 | 2 | TBCB is localized at the centrosome, and its interactions with cofactors influence the behavior of centrosomal γ-tubulin (Vadlamudi et al., 2005). The nitration of TBCB inhibits the polymerization of microtubules (MTs) by disrupting its phosphorylation process (Rayala et al., 2007). Furthermore, TBCB is modulated by the non-coding microRNA miR-1825 (Helferich et al., 2018), while TBCA is regulated by a non-coding antisense RNA during the maturation of the testis (Nolasco et al., 2012). The dynamics of MTs, which are essential for numerous cellular processes, rely on a reservoir of tubulin heterodimers that can polymerize from newly synthesized tubulins as well as from recycled tubulins derived from existing MTs. TBCB participates in the formation of a ternary complex with TBCE and γ-tubulin, and the stabilization of γ-tubulin is facilitated by TBCD or TBCA (Nolasco et al., 2021). |
| DNAH12 | 1.02E-05 | 0.529806023 | 0.614 | 0.666 | 0.1164277 | 2 | Dynein axonemal heavy chain 12 (DNAH12) or DHC3, represents a distinctive component of the dynein arm. It is noteworthy that DNAH12 is the shortest member of the DNAH family and is characterised by the absence of a conventional microtubule-binding domain (MTBD). Mutations in DNAH12 have been suggested to correlate with infertility in male patients; however, the evidence supporting its pathogenicity is limited, and the precise mechanisms of its pathogenesis remain ambiguous. Furthermore, while DNAH12 has been recognized as a potential axonemal dynein, with its mRNA predominantly found in two tissues that contain axonemal structures—namely, the trachea and testis. Its physiological roles are still largely unknown (Yang et al., 2025). |
| MICOS10 | 9.24E-06 | 0.52944912 | 0.562 | 0.603 | 0.10526455 | 2 | This protein plays a role in the structuring of the inner mitochondrial membrane. It resides within the mitochondrion and is part of multiple complexes, such as the MIB complex, MICOS complex, and SAM complex (Alkhaja et al., 2012). |
| UQCRB | 6.76E-11 | 0.529012584 | 0.783 | 0.844 | 7.71E-07 | 2 | Ubiquinol-cytochrome c oxidoreductase complex (UQCRB) consists of one subunit encoded by mitochondrial DNA and ten subunits encoded by nuclear DNA. The protein interacts with ubiquinone and participates in electron transfer when ubiquinone is present. It is crucial for hypoxia-induced angiogenesis, functioning through signaling pathways that involve mitochondrial reactive oxygen species. Alterations in this gene are linked to mitochondrial complex III deficiency (Chang et al., 2014). |
| SMC2 | 2.66E-157 | 2.072349098 | 0.739 | 0.144 | 3.03E-153 | 3 | The SMC2 subunit of the condensin complex is vital for the organization and stability of chromosomes. It regulates multiple DNA damage response (DDR) genes concurrently (Murakami-Tonami et al., 2014) and is necessary for the densification of chromosomes and the development of supercoils (Bereimipour et al., 2021). Together with SMC4, SMC2 constitutes the fundamental structure of both condensin I and condensin II complexes in higher eukaryotic organisms. These heterodimeric complexes are essential for chromosome condensation and stability during mitosis, operating as active ATPases at the center of these protein assemblies (Seipold et al., 2009) |
| TOP2A | 7.56E-220 | 2.03728058 | 0.5 | 0.03 | 8.61E-216 | 3 | Top2a is exclusively found in proliferating cells, while Top2b is broadly expressed in terminally differentiated cells, including neurons and cardiomyocytes (Tsutsui et al., 1993; Zhang et al., 2012). Top2a plays a crucial role in proliferating cells, being associated with DNA replication and the processes of chromosome condensation and segregation. In contrast, Top2b has been shown to regulate gene expression during brain development, influencing genes such as Reln, Dab1, Catna2, Cdh13, Sst, Pbx3, and Epha7 (Lyu and Wang, 2003; Nur-E-Kamal et al., 2007). Additionally, Top2b is involved in the orchestration of sister chromatid segregation and the condensation and decondensation of chromatin, working in conjunction with other chromatin-modulating enzymes like condensin (Lupo et al., 2001). Uusküla-Reimand, L. and Wilson, M.D., 2022. Untangling the roles of TOP2A and TOP2B in transcription and cancer. Science advances, 8(44), p.eadd4920. |
| HMGB2 | 1.08E-77 | 1.659648086 | 0.968 | 0.652 | 1.23E-73 | 3 | HMGB2 is predominantly localized in the outer nuclear layer (ONL) and the inner and outer segments of photoreceptors (IS/OS) within the retina. It induces photoreceptor pyroptosis through its pro-inflammatory effects and oxidative stress in light-induced retinal degeneration (LIRD) by down-regulating the Nrf2/HO-1 pathway and activating the NF-κB/NLRP3 signaling pathways. Silencing HMGB2 can reverse these effects. This research highlights the harmful role of HMGB2 in photoreceptor pyroptosis and elucidates potential mechanisms involved in the development of degenerative retinal diseases. Consequently, targeting HMGB2 may represent a promising therapeutic approach for treating such disorders (Zhang et al., 2022). Initially, HMGB2 proteins were recognized for their DNA-binding capabilities and their role in chromatin structural organization (Bustin and Reeves, 1996). Subsequent studies revealed that HMGB proteins are also involved in chromatin remodeling (Reeves, 2015). Furthermore, HMGB2 is expressed in all immortalized human and mouse cell lines, potentially aiding in the circumvention of replication constraints (Müller et al., 2004). Notably, HMGB2 (as opposed to HMGB1) has been found to inhibit the growth of malignant cells in vivo by regulating the gene expression pathways associated with hypertrophic cell growth (Franklin et al., 2012). |
| EZH2 | 1.15E-146 | 1.56730177 | 0.764 | 0.15 | 1.31E-142 | 3 | Enhancer of zeste homolog 2 (EZH2) is the enzymatic catalytic subunit of the polycomb repressive complex 2 (PRC2), responsible for trimethylating lysine 27 in histone 3 (H3K27me3), thereby influencing downstream gene expression. While EZH2 is well-known for its role in modifying histones, it can also regulate gene expression through mechanisms beyond H3K27me3. EZH2 is a critical regulator of cell proliferation, apoptosis, and senescence, functioning as a master regulator of cell cycle progression. Additionally, it promotes DNA damage repair, inhibits cellular senescence, and is involved in cell lineage determination and various signaling pathways (Duan et al., 2020). |
| STMN1 | 3.61E-75 | 1.478108901 | 0.904 | 0.498 | 4.12E-71 | 3 | Stathmin 1 (STMN1) is a cytoplasmic phosphoprotein that plays a crucial role in regulating microtubule dynamics, which subsequently affects processes such as cell differentiation, proliferation, and migration. In addition to these functions, STMN1 is implicated in DNA methylation, m6A modifications, and various facets of the immune response, including the infiltration of immune cells, the activity of immunological molecules, and the regulation of immune checkpoints. Analyses of Gene Ontology (GO) and Kyoto Encyclopedia of Genes and Genomes (KEGG) have underscored STMN1's participation in the regulation of the mitotic cell cycle, microtubule dynamics, gap junctions, FoxO signaling pathways, and apoptosis (Zhang et al., 2022). |
| CKS2 | 5.28E-57 | 1.468237999 | 0.768 | 0.386 | 6.01E-53 | 3 | CDC28 protein kinase regulatory subunit 2 (CKS2) is essential for the function of cyclin-dependent kinases (CDKs), which are critical for cell cycle control. CKS2 binds to the catalytic subunit of CDKs, facilitating their activity and playing a crucial role in the cell cycle, particularly in the transition from metaphase to anaphase during mammalian meiosis (Daniszewski et al., 2022). Mice lacking CKS2 are viable but sterile, as their germ cells cannot progress from the first meiotic metaphase to anaphase. In cancer cells, CKS2 may help protect against apoptosis. Additionally, CKS2 influences cell cycle progression and can arrest the cell cycle in the G2 phase when silenced. It is also involved in neuronal differentiation and cell division processes (Feng et al., 2022). |
| HIST1H3F | 7.11E-134 | 1.446537883 | 0.368 | 0.03 | 8.10E-130 | 3 |  |
| MCM3 | 3.82E-119 | 1.444242743 | 0.668 | 0.138 | 4.35E-115 | 3 | Minichromosome maintenance complex component 3 (MCM3) is part of the MCM2–7 complex, which forms the core of the CMG helicase complex essential for DNA unwinding. MCM3 is a substrat for ATR checkpoint kinases and was found to be phosphorylated at Ser-112 by Cdk1, and this modification regulates the assembly and activity of the MCM2–7 complex (Lin et al., 2008). |
| H2AFZ | 4.37E-72 | 1.287849879 | 1 | 0.969 | 4.98E-68 | 3 | associated with chromatin organization and promotes higher-order chromatin structures (Hao et al., 2012). |
| MCM7 | 8.60E-177 | 1.221903885 | 0.525 | 0.047 | 9.81E-173 | 3 | Minichromosome maintenance complex component 7 (MCM7) plays a key role in the G1/S transition of the mitotic cell cycle and is involved in histone exchange and DNA replication-dependent chromatin assembly.The phosphorylation of MCM7 at Ser-121 additionally aids in the assembly of the MCM complex, which is essential for a correct mitotic exit. In summary, the phosphorylation of MCM7 by Cdk is crucial for the activation of the S phase checkpoint as well as for the regulation of appropriate M phase progression (Fei et al., 2018). |
| NASP | 7.30E-63 | 1.129476132 | 0.714 | 0.263 | 8.32E-59 | 3 | NASP (nuclear autoantigenic sperm protein) NASP encodes subunits of the large histone-chaperone complex. NASP functions as a histone H1 binding protein influencing the availability of H1 histones to DNA and further affects cell cycle G1/S phase transition. NASP is highly expressed in embryonic cells, these discoveries suggest that enough level of NASP is important in the early development of the neural and immune systems. NASP is highly expressed in human brain at early developmental stage (Zhang et al., 2024). |
| DEK1 | 3.35E-58 | 1.075005336 | 0.882 | 0.517 | 3.82E-54 | 3 | DEK is a seemingly distinctive molecule whose functional role in the normal regulation of stem cells (SC) and progenitor cells (PC), as well as in leukemogenesis, is only beginning to be clarified. It is probable that DEK will be discovered to have regulatory functions in various other types of stem and progenitor cells beyond those in muscle, including embryonic stem cells, induced pluripotent stem cells, and mesenchymal stem/stromal cells (Broxmeyer et al., 2013). |
| CDCA8 | 2.48E-126 | 1.065021239 | 0.364 | 0.03 | 2.83E-122 | 3 | Cell division cycle associated 8 (CDCA8) is a crucial mitotic regulator, essential for locating the central spindle component (CPC) to the centromere, correcting kinetochore binding errors, and stabilizing bipolar spindles. It has been reported to exhibit increased transcriptional activity in embryos and embryonic stem cells (Shuai et al., 2021). |
| TYMS | 8.91E-107 | 1.064375817 | 0.439 | 0.06 | 1.02E-102 | 3 | Thymidylate synthetase (TYMS) is an enzyme essential for preserving the dTMP (thymidine-5-prime monophosphate) pool, which is vital for DNA synthesis and repair (Kotoula et al., 2012). |
| CDK1 | 6.79E-105 | 1.030468156 | 0.421 | 0.055 | 7.74E-101 | 3 | Cluster 3 is indicative of RPCs. RPCs can be marked in cluster analysis by high expression of proliferation genes, such as Top2a which is highly expressed in proliferating cells (Dorgau et al., 2024). Top2a plays a crucial role in proliferating cells, being associated with DNA replication and the processes of chromosome condensation and segregation (Capranico et al., 1992). Deletion of Top2a in murine models leads to significant disruptions in nuclear division, ultimately resulting in embryonic mortality at the four-cell stage. Furthermore, Top2a plays a crucial role in preserving chromosomal integrity, as its swift degradation throughout the cell cycle interferes with the correct formation of mitotic chromosomes and initiates premature mitotic exit (Uusküla-Reimand et al., 2022).  CDK1, a cyclin-dependent kinase that forms a regulatory complex with cyclin B1, plays a pivotal role in orchestrating events during the late G2 and M phases of the cell cycle, thereby driving progenitor cells into mitosis. Its critical function in cell cycle progression positions CDK1 as a reliable marker of actively dividing progenitors. Studies have highlighted the importance of upstream signalling pathways in modulating CDK1 activity, particularly the PI3K/Akt pathway, which has been shown to regulate the G2/M transition across various cell types (Ornelas et al., 2023). CDK1 is involved in several cellular processes, including mitosis, transcriptional regulation, cell polarisation, and DNA damage repair. Recent research has also highlighted its role in cell death (Liaoet al., 2017). CDK1 triggers mitosis and is expressed in degenerating photoreceptors, especially in the later stages of cell death. This suggests that CDK1 may either contribute to the degeneration process or be involved in a counteractive response. Functional studies of CDK1 indicate that it upregulates mitochondrial respiration to support the energy demands of the cell cycle (Wang et al., 2014). Its role in retinal degeneration, particularly during the late stages of photoreceptor death, has been noted (Zhou et al., 2022). While the spatial closeness of microglia in cluster 0, RPCs in cluster 3 and dividing cells in cluster 2 indicates a greater level of transcriptional resemblance and potential developmental relationships, implying that these cell populations may function as proliferative precursors within a shared retinal lineage. However, the microglial cells in cluster 0 exhibit a lesser overlap with these groups and maintain a separate cluster, likely indicative of shared pathways related to the cell cycle, yet they also display a distinct gene expression profile typical of immune cells. |
| RANBP1 | 9.42E-52 | 1.008599832 | 0.864 | 0.512 | 1.07E-47 | 3 | Nuclear export of LKB1 is regulated by Ran GTPase and its effector Ran-binding protein 1 (RanBP1), which is crucial for the polarization of epithelial cells and rat cortical neurons (Baas et al., 2003). This process is required for proper axon formation and neuron migration during cortical development (Mencarelli et al., 2018). RanBP1 is essential for cell viability in both cultured cortical neurons and neural crest (NC) cells, and its absence affects cell survival and neuron migration (Barriga et al., 2022). Ran GTPase acts as a master regulatory switch of nucleocytoplasmic trafficking, with its active form, Ran-GTP, bind (Patil et al., 2013). |
| TUBB | 2.40E-65 | 0.997707578 | 0.996 | 0.912 | 2.74E-61 | 3 | Tubulin β (TUBB) is a gene that encodes a protein, which plays a crucial role in forming a heterodimer with alpha-tubulin and serves as a fundamental structural element of microtubules.Microtubules are crucial in regulating various elements of cellular architecture and functionality. They are vital for motor-driven intracellular transport, engage with accessory proteins to form larger structures, and collaborate with other cell types to establish a mature network (Zhu et al., 2024). |
| SMC1A | 8.96E-48 | 0.970936384 | 0.889 | 0.524 | 1.02E-43 | 3 | SMC1A part of the structural maintenance of chromosomes (SMC) family, SMC1A is evolutionarily conserved and encodes a structural element of the cohesin complex, which is essential for accurate chromosome segregation. As a subunit of cohesin, it also contributes to the organization of the 3D genome and the regulation of gene expression. Furthermore, substantial experimental evidence suggests that SMC1A is involved in the DNA repair mechanisms and the maintenance of genomic stability (Musio, 2020). |
| SNRPD1 | 1.40E-51 | 0.967210868 | 0.875 | 0.499 | 1.60E-47 | 3 | The spliceosome is a ribonucleoprotein (RNP) characterized by a complex ring-like structure, primarily composed of small nuclear ribonucleoproteins (snRNPs), which play a crucial role in the splicing of pre-mRNA into mature mRNA. Seven Sm proteins (SNRPB, SNRPD1, SNRPD2, SNRPD3, SNRPE, SNRPF, and SNRPG) along with a small eponymous small nuclear RNA (snRNA) make up the snRNPs. Precise splicing is vital for normal cellular processes such as cell proliferation, apoptosis, migration, and invasion.(Liu et al., 2022). |
| HELLS | 1.77E-158 | 0.96620086 | 0.332 | 0.016 | 2.01E-154 | 3 | Lymphoid-specific helicase (HELLS) is an epigenetic modifier tightly regulated by the RB/E2F pathway. It functions to remodel chromatin, making DNA accessible to DNA methyltransferase enzymes, such as Dnmt3a and Dnmt3b, which are critical for de novo DNA methylation and the stable silencing of genes during cellular differentiation (Burrage et al., 2012). HELLS also binds to DNMT1, HDAC1, and HDAC2, linking it to other key regulators of chromatin modification and gene silencing (Myant et al., 2008). HELLS plays an essential role in normal development. HELLS knockout mice experience perinatal lethality, highlighting its importance in survival. Mutant mice exhibit growth retardation, premature aging, and impaired self-renewal and maintenance of neural stem/progenitor cells during development (Sun et a., 2004).  In terms of DNA methylation, HELLS is required for either de novo or maintenance methylation, potentially controlling the silencing of imprinted genes, such as CDKN1C, through this mechanism. Furthermore, HELLS is implicated in the formation and organization of heterochromatin, contributing to the regulation of both transcription and mitosis (Zocchi et al., 2020). |
| PCNA | 8.08E-48 | 0.919454557 | 0.589 | 0.215 | 9.20E-44 | 3 | Proliferating Cell Nuclear Antigen (PCNA) is expressed in retinal progenitor cells (RPCs) throughout the proliferative phase and is detectable in all phases of the cell cycle. PCNA is widely used as a marker for proliferating cells due to its ubiquitous expression in dividing cells. In the developing retina, PCNA is primarily detected in the neuroblast layer (NBL), but is excluded from cells in the developing ganglion cell layer and the inner boundary of the NBL, which is referred to as the differentiated cell layer. This distinct expression pattern makes PCNA the most reliable marker for identifying RPCs, as it serves as a comprehensive and definitive marker of these proliferative retinal cells (Barton et al., 2008). |
| CENPF | 7.94E-81 | 0.906223534 | 0.271 | 0.028 | 9.04E-77 | 3 | Centromere Protein F (CENPF) is a mitotic centromere protein that plays a key role in chromosome segregation during mitosis. Its expression follows a cell cycle-dependent pattern, with a peak occurring during the G2/M phase of the cell cycle. CENPF is crucial for cell proliferation and has been implicated in the migration, invasion, and inhibition of apoptosis in cancer cells. This protein is closely linked to the oncogenesis of various neoplasms, making it a critical factor in tumor development and progression (Huang et al., 2022). |
| PRC1 | 5.49E-133 | 0.905076929 | 0.264 | 0.011 | 6.26E-129 | 3 | The binding of Polycomb repressive complex 1 (PRC1( alone can modify the structure of chromatin; when PRC1 is pre-incubated, it inhibits the formation of RNA polymerase II pre-initiation complexes on DNA. The repression mediated by PRC1 is particularly essential during lineage commitment and differentiation, leading to an increase in the relative protein expression of canonical PRC1 from less than 10% of all PRC1 complexes in embryonic stem cells to more than 15% in neural progenitor cells. (Wang et al., 2018) |
| ATAD2 | 1.33E-86 | 0.890616754 | 0.421 | 0.067 | 1.52E-82 | 3 | ATPase family AAA domain-containing protein 2 (ATAD2) is a recently identified oncogene that belongs to the ATPase family. It contains two conserved domains: a bromodomain, which is responsible for histone binding, and an ATPase domain, which regulates ATPase activity and protein multimerization (Lui et al., 2019). As an epigenetic regulator, ATAD2 is primarily known for its role in histone modification. Mechanistically, ATAD2 binds directly to chromatin by identifying histone acetylation, which increases chromatin accessibility and histone dynamics, creating conditions for the appropriate activity of highly expressed genes. Furthermore, ATAD2 maintains specific gene expression programs by regulating chromatin dynamics through histone acetylation-directed chromatin opening (Fu et al., 2023). |
| TOPBP1 | 6.57E-79 | 0.89006579 | 0.439 | 0.08 | 7.49E-75 | 3 | DNA topoisomerase II-binding protein 1 (TOPBP1) is a crucial DNA-damage response mediator. It binds to specific motifs and interacts with MDC1, which is essential for its recruitment to double-strand breaks (DSBs) during mitosis. TOPBP1 is predicted to have identical protein binding activity and is involved in DNA replication initiation, the mitotic G2/M transition checkpoint, and the response to ionizing radiation (Leimbacher et al., 2019). |
| USP1 | 1.65E-73 | 0.888420376 | 0.507 | 0.112 | 1.88E-69 | 3 | Ubiquitin Specific Peptidase 1 (USP1) is a deubiquitinating enzyme (DUB) involved in the Fanconi anemia (FA) DNA repair pathway. It is highly expressed during retinal development but its expression decreases as retinal differentiation progresses. USP1, which contains His and Cys domains, is located in the cytoplasm and removes ubiquitin from ubiquitin-fused precursors and proteins (Coleman et al., 2022).  The protein plays a crucial role in maintaining genome stability by dynamically associating with the replication fork. Under normal conditions, USP1 deubiquitinates PCNA (proliferating cell nuclear antigen) and other substrates to regulate replication timing. During replication stress, USP1 undergoes autocleavage, which displaces it from stalled forks, allowing an increase in PCNA ubiquitination. This change facilitates translesion synthesis (TLS) and other stress responses necessary for efficient DNA repair (Coleman et al., 2022). |
| HIST1H3H | 8.03E-94 | 0.878472638 | 0.279 | 0.024 | 9.15E-90 | 3 |  |
| POLA1 | 1.29E-107 | 0.849098331 | 0.371 | 0.039 | 1.47E-103 | 3 | The catalytic subunit of the DNA polymerase alpha complex, known as POLA1, is crucial for the initiation of DNA synthesis. During the S phase of the cell cycle, POLA1, which encodes the catalytic unit of DNA polymerase α, works in conjunction with the Primase complex to start DNA replication (Starokadomskyy et al., 2021) |
| SRSF7 | 6.96E-39 | 0.841537093 | 0.907 | 0.582 | 7.93E-35 | 3 | Serine and arginine-rich splicing factor 7 (SRSF7) plays a significant role in regulating its own splicing. In wild-type mouse cells, SRSF7 induces its binding to its own transcripts, promoting the inclusion of its poison exon, which results in reduced levels of functional SRSF7 protein (Königs et al., 2020).  SRSF7 functions as a constitutive and alternative splicing activator and is involved in various processes including mRNA export, alternative polyadenylation, (viral) mRNA processing, translation, miRNA processing, nuclear body assembly, and nonsense-mediated decay (Müller-McNicoll et al., 2016; Änkö et al., 2014; Slišković et al., 2022). |
| HAT1 | 4.07E-64 | 0.83477128 | 0.464 | 0.106 | 4.64E-60 | 3 | Cytoplasmic histone acetyltransferase 1 (HAT1) is an enzyme initially identified in S. cerevisiae for its activity towards H4K12 residues. In mice, HAT1 is essential for proper development; its absence results in lung and cranial developmental defects and neonatal death (Varga et al., 2019).  HAT1, a type B histone acetyltransferase, acetylates histone targets including H2AK7, H4K5, H4K8, and H4K12. It acetylates H4K5 and H4K12 before nuclear deposition and chromatin assembly, and also coordinates histone production and acetylation via H4 promoter binding (Zhao et al., 2020.   Functional enrichment analysis reveals that HAT1 is crucial for the hypoxia response. HAT1 acetylates lysine residues 512 and 596 of HIF2A, stabilizing it and enhancing the hypoxia response, particularly in glioma. This stabilization of HIF2A by HAT1 facilitates gliomagenesis and the maintenance of glioma stem-like cells. Wnt/β-catenin signaling drives HAT1 expression, which interacts with and acetylates HIF2A, contributing to effective hypoxia responses in glioma (Kumar et al., 2023). |
| CDCA3 | 2.77E-80 | 0.798085862 | 0.286 | 0.031 | 3.16E-76 | 4 | Cell division cycle-associated protein 3 (CDCA3) is crucial for cell cycle regulation. The protein encoded by CDCA3 triggers cell entry into mitosis by activating cyclin-dependent kinase 1 (CDK1) and cyclin B. Additionally, CDCA3 influences cell cycle progression by affecting DNA methylation (Xu et al., 2022). |
| NEUROD1 | 2.50E-257 | 5.046704752 | 0.552 | 0.018 | 2.85E-253 | 4 | In the retina, NeuroD1 is crucial for the terminal differentiation and survival of rod photoreceptors, with its loss leading to progressive photoreceptor degeneration (Morrow et al., 1999). NeuroD1 also plays a role in cone photoreceptor patterning and regulates cone-specific expression by modulating thyroid hormone receptor β2 expression during development (Liu et al., 2008). It is essential for photoreceptor function and may act through a specific set of genes to promote retinal survival, particularly by maintaining the photoreceptor outer segments(Ochocinska et al., 2012). |
| RORB | 0 | 4.279707875 | 0.922 | 0.012 | 0 | 4 | Rorb is expressed in all neural retina layers from early developmental stages, peaking at neonatal stages, indicating its involvement in various differentiating retinal cell types, including both cones and rods (Schaeren‐Wierners et al., 1997). It is essential for rod development and acts upstream of Nrl and Nr2e3 to influence whether a photoreceptor precursor develops into a rod or cone. Rorb is broadly expressed in the immature retina and plays dual roles in promoting the differentiation of both rods and cones. Previous studies have shown that Rorb, in conjunction with Crx, synergistically activates the S opsin gene (Opn1sw) promoter during normal cone development (Jia et al., 2009). |
| OTX2 | 0 | 3.868155506 | 0.545 | 0.009 | 0 | 4 | During the process of retinal development, the transcription factor Otx2 is significantly upregulated in a substantial number of progenitor cells, which is essential for the development of photoreceptors and bipolar cells. The expression of Otx2 initiates around embryonic day (E) 12, aligning with the earliest emergence of photoreceptors. Its upregulation occurs as progenitor cells leave the cell cycle, leading to the generation of various neuronal types, such as horizontal, amacrine, and bipolar interneurons, in addition to rod and cone photoreceptors. Although Otx2 is quickly diminished in amacrine and horizontal cells, it remains present in photoreceptors and bipolar cells throughout adulthood (Kaufman et al., 2021) |
| MEIS2 | 0 | 3.623751695 | 0.506 | 0.007 | 0 | 4 | Meis2 is crucial for the regionalization of the optic cup, promoting retinal progenitor cell (RPC) fate while restricting the fates of the ciliary marginal zone (CMZ) and optic disc (OD) (Agoston et al., 2014). Meis2 interacts directly with Pax6 and Dlx2, working together during subventricular zone neurogenesis. Both Meis1 and Meis2 are expressed in the neuroepithelium, with complete depletion typically occurring by embryonic day (E) 14.5. However, patches of reduced Meis protein levels have been observed as early as E11.5. Although Meis1/2 directly bind to regulatory regions of CMZ-specific genes, it is still unclear if these enhancers are active in the early neuroepithelium, CMZ, or both (Dupacova et al., 2021). |
| PFN2 | 0 | 3.277686053 | 0.721 | 0.025 | 0 | 4 | Profilin 2 (PFN2) is an actin-binding protein that regulates actin polymerization dynamics and is crucial for cell motility. It accelerates the extension of uncapped actin filaments in the presence of formin and then promotes the depolymerization of capped actin filaments, leading to changes in the cytoskeleton (Cui et al., 2016). |
| PTN | 0 | 3.262045771 | 0.487 | 0.004 | 0 | 4 | Pleiotrophin (Ptn), also known as HB-GAM (heparin-binding growth-associated molecule), and Midkine are members of the heparin-binding growth factor family. These factors play diverse biological roles, including involvement in neurogenesis by promoting neurite outgrowth and nerve cell migration. Ptn has recently been highlighted for its role in regulating stem cell proliferation and differentiation. During retinal development, Ptn expression aligns with the differentiation of late retinal progenitor cells and the formation of the inner nuclear layer. Overexpression of Ptn in the newborn retina can block rod differentiation and promote bipolar cell genesis, influenced by exogenous cytokines such as CNTF or LIF. Ptn's expression in proliferating progenitor cells can direct them towards a bipolar cell fate and control photoreceptor differentiation by suppressing rhodopsin expression. Additionally, Ptn released by Muller and bipolar cells under the influence of CNTF may positively affect the differentiation of retinal progenitors into bipolar cells, while CNTF or LIF may control rod differentiation by suppressing rhodopsin expression independently of Ptn release (Roger et al., 2006). |
| SOX4 | 5.27E-73 | 3.255251924 | 0.896 | 0.458 | 6.00E-69 | 4 | Sox4 and Sox11 are crucial for the development of retinal ganglion cells (RGCs), playing significant roles in their differentiation and survival. These factors are involved in both promoting RGC differentiation and regulating cell survival during retinogenesis. Sox4 and Sox11 operate within the same pathway, influencing the proliferation and survival of post-mitotic RGCs. Their expression in the neuroblast layer (NBL) and the ganglion cell layer (GCL) during early retinal development suggests that they are key in driving RGC fate commitment (Jiang et al., 2013) |
| PSIP1 | 2.63E-172 | 3.239131333 | 0.857 | 0.126 | 2.99E-168 | 4 | PC4- and SF2-interacting protein 1 (PSIP1) is known for its role in gene regulation within epithelial cells of the lens and in cell fate determination. Additionally, PSIP1 has been implicated in neuroepithelial differentiation and neurogenesis (Girotto et al., 2015). |
| DMD | 1.98E-206 | 3.171139942 | 0.461 | 0.016 | 2.25E-202 | 4 | Visual processing in vertebrates initiates with retinal photoreceptors, which encompass both cones and rods that hyperpolarize in response to light via phototransduction. Despite their distinct anatomical and functional characteristics, both photoreceptor types play a role in the preliminary phases of visual information processing. They relay visual signals to On- and Off-bipolar cells, in addition to horizontal cells. Within the retina, dystrophins Dp427, Dp260, and Dp140 are primarily expressed at the ribbon synapses of photoreceptors, where cones and rods form connections with On-bipolar cells and horizontal cells. These dystrophins are also present at flat synapses where cones interact with Off-bipolar cells. Furthermore, dystrophin Dp71 is crucial for the proper functioning of retinal glial cells (Barboni et al., 2023). |
| SIX3 | 0 | 3.094251961 | 0.714 | 0.008 | 0 | 4 | Six3, along with Six6 at later stages, is crucial for determining cell fate in neural retina (NR) progenitors by suppressing Wnt/β-catenin signaling. Initially, Six3 represses Wnt1 to promote the development of rostral forebrain and eye field precursors, preventing the adoption of caudal forebrain cell fates (Lagutin et al., 2003). Together, Six3 and Six6 form an additional regulatory program necessary to maintain the multipotency of NR progenitors (Diacou et al., 2018). |

**Bibliography**

Adam, K., Lesperance, J., Hunter, T. and Zage, P.E., 2020. The potential functional roles of NME1 histidine kinase activity in neuroblastoma pathogenesis. International journal of molecular sciences, 21(9), p.3319.

Agoston, Z., Heine, P., Brill, M.S., Grebbin, B.M., Hau, A.C., Kallenborn-Gerhardt, W., Schramm, J., Götz, M. and Schulte, D., 2014. Meis2 is a Pax6 co-factor in neurogenesis and dopaminergic periglomerular fate specification in the adult olfactory bulb. Development, 141(1), pp.28-38.

Alkhaja, A.K., Jans, D.C., Nikolov, M., Vukotic, M., Lytovchenko, O., Ludewig, F., Schliebs, W., Riedel, D., Urlaub, H., Jakobs, S. and Deckers, M., 2012. MINOS1 is a conserved component of mitofilin complexes and required for mitochondrial function and cristae organization. Molecular biology of the cell, 23(2), pp.247-257.

Alrefaei, A.F. and Abu-Elmagd, M., 2022. LRP6 receptor plays essential functions in development and human diseases. Genes, 13(1), p.120.

Al-Shabrawey, M., Hussein, K., Wang, F., Wan, M., Elmasry, K., Elsherbiny, N., Saleh, H., Yu, P.B., Tawfik, A. and Ibrahim, A.S., 2021. Bone morphogenetic protein-2 induces non-canonical inflammatory and oxidative pathways in human retinal endothelial cells. Frontiers in immunology, 11, p.568795.

Alvarez, L., Gonzalez-Iglesias, H., Garcia, M., Ghosh, S., Sanz-Medel, A. and Coca-Prados, M., 2012. The stoichiometric transition from Zn6Cu1-metallothionein to Zn7-metallothionein underlies the upregulation of metallothionein (MT) expression: quantitative analysis of MT-metal load in eye cells. Journal of Biological Chemistry, 287(34), pp.28456-28469.

Álvarez-Barrios, A., Álvarez, L., García, M., Artime, E., Pereiro, R. and González-Iglesias, H., 2021. Antioxidant defenses in the human eye: A focus on metallothioneins. Antioxidants, 10(1), p.89.

Anctil, M. and Ali, M.A., 1974. Giant ganglion cells in the retina of the hammerhead shark (Sphyrna lewini). Vision Research, 14(9), pp.903-IN3.

Änkö, M.L., 2014, August. Regulation of gene expression programmes by serine–arginine rich splicing factors. In Seminars in cell & developmental biology(Vol. 32, pp. 11-21). Academic Press.

Arroba, A.I., Campos-Caro, A., Aguilar-Diosdado, M. and Valverde, Á.M., 2018. IGF-1, inflammation and retinal degeneration: a close network. Frontiers in aging neuroscience, 10, p.203.

Arteaga, M.F., Coric, T., Straub, C. and Canessa, C.M., 2008. A brain-specific SGK1 splice isoform regulates expression of ASIC1 in neurons. Proceedings of the National Academy of Sciences, 105(11), pp.4459-4464.

Aso, T., Haque, D., Barstead, R.J., Conaway, R.C. and Conaway, J.W., 1996. The inducible elongin A elongation activation domain: structure, function and interaction with the elongin BC complex. The EMBO journal, 15(20), pp.5557-5566.

Atkinson, S.P., Collin, J., Irina, N., Anyfantis, G., Kyung, B.K., Lako, M. and Armstrong, L., 2012. A putative role for the immunoproteasome in the maintenance of pluripotency in human embryonic stem cells. Stem cells, 30(7), pp.1373-1384.

Audouard, C. and Christians, E., 2011. Hsp90b1 knockout targeted to male germline: a mouse model for globozoospermia. Fertility and sterility, 95(4), pp.1475-1477.

Ayer, A., Zarjou, A., Agarwal, A. and Stocker, R., 2016. Heme oxygenases in cardiovascular health and disease. Physiological reviews, 96(4), pp.1449-1508.

Baas, A.F., Boudeau, J., Sapkota, G.P., Smit, L., Medema, R., Morrice, N.A., Alessi, D.R. and Clevers, H.C., 2003. Activation of the tumour suppressor kinase LKB1 by the STE20‐like pseudokinase STRAD. The EMBO journal.

Bachg, A.C., Horsthemke, M., Skryabin, B.V., Klasen, T., Nagelmann, N., Faber, C., Woodham, E., Machesky, L.M., Bachg, S., Stange, R. and Jeong, H.W., 2019. Phenotypic analysis of Myo10 knockout (Myo10 tm2/tm2) mice lacking full-length (motorized) but not brain-specific headless myosin X. Scientific Reports, 9(1), p.597.

Barboni, M.T.S., Joachimsthaler, A., Roux, M.J., Nagy, Z.Z., Ventura, D.F., Rendon, A., Kremers, J. and Vaillend, C., 2023. Retinal dystrophins and the retinopathy of Duchenne muscular dystrophy. Progress in Retinal and Eye Research, 95, p.101137.

Baretić D, Jenkyn-Bedford M, Aria V, Cannone G, Skehel M, Yeeles JTP. Cryo-EM Structure of the Fork Protection Complex Bound to CMG at a Replication Fork. Molecular Cell. 2020;78:926–940.

Barriga, E.H., Alasaadi, D.N., Mencarelli, C., Mayor, R. and Pichaud, F., 2022. RanBP1 plays an essential role in directed migration of neural crest cells during development. Developmental Biology, 492, pp.79-86.

Barton, K.M. and Levine, E.M., 2008. Expression patterns and cell cycle profiles of PCNA, MCM6, cyclin D1, cyclin A2, cyclin B1, and phosphorylated histone H3 in the developing mouse retina. Developmental dynamics: an official publication of the American Association of Anatomists, 237(3), pp.672-682.

Bates, A.S., Evans, A.J., Jones, P. and Clayton, R.N., 1995. Assessment of GH status in adults with GH deficiency using serum growth hormone, serum insulin‐like growth factor‐l and urinary growth hormone excretion. Clinical Endocrinology, 42(4), pp.425-430.

Baumann, B.H., Shu, W., Song, Y., Simpson, E.M., Lakhal-Littleton, S. and Dunaief, J.L., 2019. Ferroportin-mediated iron export from vascular endothelial cells in retina and brain. Experimental eye research, 187, p.107728.

Bazan, N. G., & Scott, B. L. 1990. Dietary omega-3 fatty acids and accumulation of docosahexaenoic acid in rod photoreceptor cells of the retina and at synapses. Upsala journal of medical sciences. Supplement, 48, 97–107.

Beli, P., Lukashchuk, N., Wagner, S.A., Weinert, B.T., Olsen, J.V., Baskcomb, L., Mann, M., Jackson, S.P. and Choudhary, C., 2012. Proteomic investigations reveal a role for RNA processing factor THRAP3 in the DNA damage response. Molecular cell, 46(2), pp.212-225.

Ben-Porath, I., Kozak, C.A. and Benvenisty, N., 1998. Chromosomal Mapping ofTmp (Emp1), Xmp (Emp2), andYmp (Emp3), Genes Encoding Membrane Proteins Related toPmp22. Genomics, 49(3), pp.443-447.

Bereimipour, A., Satarian, L. and Taleahmad, S., 2021. Investigation of Key Signaling Pathways Associating miR‐204 and Common Retinopathies. BioMed research international, 2021(1), p.5568113.

Berger M., Wraith K., Woodward C., Aburima A., Raslan Z., Hindle M. S., Moellmann J., Febbraio M., and Naseem K. M., Dyslipidemia-associated atherogenic oxidized lipids induce platelet hyperactivity through phospholipase Cγ2-dependent reactive oxygen species generation, Platelets. (2019) 30, no. 4, 467–472

Bharadwaj, A.S., Appukuttan, B., Wilmarth, P.A., Pan, Y., Stempel, A.J., Chipps, T.J., Benedetti, E.E., Zamora, D.O., Choi, D., David, L.L. and Smith, J.R., 2013. Role of the retinal vascular endothelial cell in ocular disease. Progress in retinal and eye research, 32, pp.102-180.

Bhattacharjee, J. and Sanyal, S., 1975. Developmental changes of esterases in the retina of the mouse: histochemical study. Histochemistry, 46, pp.53-60.

Bhattacharjee, J. and Sanyal, S., 1975. Developmental origin and early differentiation of retinal Müller cells in mice. Journal of anatomy, 120(Pt 2), p.367.

Bhattacharjee, J., 1976. Developmental changes of carbonic anhydrase in the retina of the mouse: a histochemical study. The Histochemical Journal, 8, pp.63-70.

Bhattacharya, S., Michels, C.L., Leung, M.K., Arany, Z.P., Kung, A.L. and Livingston, D.M., 1999. Functional role of p35srj, a novel p300/CBP binding protein, during transactivation by HIF-1. Genes & development, 13(1), pp.64-75.

Biname, F.; Bidaud-Meynard, A.; Magnan, L.; Piquet, L.; Montibus, B.; Chabadel, A.; Saltel, F.; Lagree, V.; Moreau, V. Cancer-associated mutations in the protrusion-targeting region of p190RhoGAP impact tumor cell migration. J. Cell Boil. 2016, 214, 859–873.

Björkblom, B., Padzik, A., Mohammad, H., Westerlund, N., Komulainen, E., Hollos, P., Parviainen, L., Papageorgiou, A.C., Iljin, K., Kallioniemi, O. and Kallajoki, M., 2012. c-Jun N-terminal kinase phosphorylation of MARCKSL1 determines actin stability and migration in neurons and in cancer cells. Molecular and cellular biology, 32(17), pp.3513-3526.

Blume, Z. I., Lambert, J. M., Lovel, A. G., & Mitchell, D. M. (2020). Microglia in the developing retina couple phagocytosis with the progression of apoptosis via P2RY12 signaling. Developmental dynamics : an official publication of the American Association of Anatomists, 249(6), 723–740.

Boissan, M., Montagnac, G., Shen, Q., Griparic, L., Guitton, J., Romao, M., Sauvonnet, N., Lagache, T., Lascu, I., Raposo, G. and Desbourdes, C., 2014. Nucleoside diphosphate kinases fuel dynamin superfamily proteins with GTP for membrane remodeling. Science, 344(6191), pp.1510-1515.

Bonelli, R., Jackson, V.E., Prasad, A., Munro, J.E., Farashi, S., Heeren, T.F., Pontikos, N., Scheppke, L., Friedlander, M., MacTel Consortium and Egan, C.A., 2021. Identification of genetic factors influencing metabolic dysregulation and retinal support for MacTel, a retinal disorder. Communications biology, 4(1), p.274.

Bouchard, C., Pagé, J., Bédard, A., Tremblay, P. and Vallières, L., 2007. G protein‐coupled receptor 84, a microglia‐associated protein expressed in neuroinflammatory conditions. Glia, 55(8), pp.790800.

Bradsher, J.N., Jackson, K.W., Conaway, R.C. and Conaway, J.W., 1993. RNA polymerase II transcription factor SIII. I. Identification, purification, and properties. Journal of Biological Chemistry, 268(34), pp.25587-25593.

Brekken, R.A. and Sage, E.H., 2000. SPARC, a matricellular protein: at the crossroads of cell– matrix. Matrix Biology, 19(7), pp.569-580.

Broxmeyer, H.E., Mor-Vaknin, N., Kappes, F., Legendre, M., Saha, A.K., Ou, X., O'leary, H., Capitano, M., Cooper, S. and Markovitz, D.M., 2013. Concise review: role of DEK in stem/progenitor cell biology. Stem Cells, 31(8), pp.1447-1453.

Burguillos, M.A., Svensson, M., Schulte, T., Boza-Serrano, A., Garcia-Quintanilla, A., Kavanagh, E., Santiago, M., Viceconte, N., Oliva-Martin, M.J., Osman, A.M. and Salomonsson, E., 2015. Microgliasecreted galectin-3 acts as a toll-like receptor 4 ligand and contributes to microglial activation. Cell reports, 10(9), pp.1626-1638.

Burrage, J., Termanis, A., Geissner, A., Myant, K., Gordon, K. and Stancheva, I., 2012. The SNF2 family ATPase LSH promotes phosphorylation of H2AX and efficient repair of DNA double-strand breaks in mammalian cells. Journal of cell science, 125(22), pp.5524-5534.

Bustin, M. and Reeves, R., 1996. High-mobility-group chromosomal proteins: architectural components that facilitate chromatin function. Progress in nucleic acid research and molecular biology, 54, pp.35-100b.

Butovsky, O., Jedrychowski, M. P., Moore, C. S., Cialic, R., Lanser, A. J., Gabriely, G., Koeglsperger, T., Dake, B., Wu, P. M., Doykan, C. E., Fanek, Z., Liu, L., Chen, Z., Rothstein, J. D., Ransohoff, R. M., Gygi, S. P., Antel, J. P., & Weiner, H. L. (2014). Identification of a unique TGF-β-dependent molecular and functional signature in microglia. Nature neuroscience, 17(1), 131–143.

Cai, H., Fields, M.A., Hoshino, R. and Priore, L.V.D., 2012. Effects of aging and anatomic location on gene expression in human retina. Frontiers in aging neuroscience, 4, p.8.

Cai, S., Yang, Q., Cao, Y., Li, Y., Liu, J., Wang, J., Zhang, X., Liu, L., Li, X. and Zhang, Y., 2020. PF4 antagonizes retinal neovascularization via inhibiting PRAS40 phosphorylation in a mouse model of oxygen-induced retinopathy. Biochimica et Biophysica Acta (BBA)-Molecular Basis of Disease, 1866(3), p.165604.

Calandra, T. and Roger, T., 2003. Macrophage migration inhibitory factor: a regulator of innate immunity. Nature reviews immunology, 3(10), pp.791-800.

Caridi, B., Doncheva, D., Sivaprasad, S. and Turowski, P., 2021. Galectins in the pathogenesis of common retinal disease. Frontiers in Pharmacology, 12, p.687495.

Casano, A.M., Albert, M. and Peri, F., 2016. Developmental apoptosis mediates entry and positioning of microglia in the zebrafish brain. Cell Reports, 16(4), pp.897-906.

Casaroli-Marano, R.P., Peinado-Onsurbe, J., Reina, M., Staels, B., Auwerx, J. and Vilaro, S., 1996. Lipoprotein lipase in highly vascularized structures of the eye. Journal of lipid research, 37(5), pp.1037-1044.

Chakravarty, G.; Hadsell, D.; Buitrago, W.; Settleman, J.; Rosen, J.M. p190-B RhoGAP regulates mammary ductal morphogenesis. Mol. Endocrinol. 2003, 17, 1054–1065.

Chang, J., Jung, H.J., Jeong, S.H., Kim, H.K., Han, J. and Kwon, H.J., 2014. A mutation in the mitochondrial protein UQCRB promotes angiogenesis through the generation of mitochondrial reactive oxygen species. Biochemical and biophysical research communications, 455(3-4), pp.290-297.

Chen, H. Y., Hu, J. Y., Chen, T. H., Lin, Y. C., Liu, X., Lin, M. Y., Lang, Y. D., Yen, Y., & Chen, R. H. (2015). KLHL39 suppresses colon cancer metastasis by blocking KLHL20-mediated PML and DAPK ubiquitination. Oncogene, 34(40), 5141–5151.

Chen, M., Chen, Y., Fu, R., Liu, S., Li, H. and Shen, T., 2024. Atox1 regulates macrophage polarization in intestinal inflammation via ROS-NLRP3 inflammasome pathway. Journal of Translational Medicine, 22(1), p.497.

Chen, X., Lin, R., Chang, L., Xu, S., Wei, X., Zhang, J., Wang, C., Anwyl, R. and Wang, Q., 2013. Enhancement of long-term depression by soluble amyloid β protein in rat hippocampus is mediated by metabotropic glutamate receptor and involves activation of p38MAPK, STEP and caspase-3. Neuroscience, 253, pp.435-443.

Chen, Xiaowei, Yu Wang, Fang Xu, Xiaofei Wei, Junfang Zhang, Chuang Wang, Hua Wei et al. "The rapid effect of bisphenol‐A on long‐term potentiation in hippocampus involves estrogen receptors and ERK activation." Neural plasticity 2017, no. 1 (2017): 5196958.

Chen, Y., Chen, Y., Yin, W., Han, H., Miller, H., Li, J., Herrada, A.A., Kubo, M., Sui, Z., Gong, Q. and Liu, C., 2021. The regulation of DOCK family proteins on T and B cells. Journal of Leucocyte Biology, 109(2), pp.383-394.

Chen, Y., Doughman, Y.Q., Gu, S., Jarrell, A., Aota, S.I., Cvekl, A., Watanabe, M., Dunwoodie, S.L., Johnson, R.S., van Heyningen, V. and Kleinjan, D.A., 2008. Cited2 is required for the proper formation of the hyaloid vasculature and for lens morphogenesis.

Chen, Y., Hu, Y., Lu, K., Flannery, J.G. and Ma, J.X., 2007. Very low density lipoprotein receptor, a negative regulator of the wnt signaling pathway and choroidal neovascularization. Journal of Biological Chemistry, 282(47), pp.34420-34428.

Chen, Y., Zhang, T., Zeng, S., Xu, R., Jin, K., Coorey, N.J., Wang, Y., Wang, K., Lee, S.R., Yam, M. and Zhu, M., 2022. Transketolase in human Müller cells is critical to resist light stress through the pentose phosphate and NRF2 pathways. Redox Biology, 54, p.102379.

Cheong, S.M., Choi, S.C. and Han, J.K., 2006. Xenopus Dab2 is required for embryonic angiogenesis. BMC developmental biology, 6, pp.1-14.

Chidlow, G., Wood, J.P., Knoops, B. and Casson, R.J., 2016. Expression and distribution of peroxiredoxins in the retina and optic nerve. Brain Structure and Function, 221, pp.3903-3925.

Chiha, W., LeVaillant, C.J., Bartlett, C.A., Hewitt, A.W., Melton, P.E., Fitzgerald, M. and Harvey, A.R., 2018. Retinal genes are differentially expressed in areas of primary versus secondary degeneration following partial optic nerve injury. PLoS One, 13(2), p.e0192348.

Chintalapudi, S.R., Wang, X., Li, H., Lau, Y.H.C., Williams, R.W. and Jablonski, M.M., 2016. Genetic and immunohistochemical analysis of HSPA5 in mouse and human retinas. Molecular Vision, 22, p.1318.

Cho, Y.E., Ko, J.H., Kim, Y.J., Yim, J.H., Kim, S.M. and Park, J.H., 2007. mHGTD-P mediates hypoxic neuronal cell death via the release of apoptosis-inducing factor. Neuroscience letters, 416(2), pp.144149.

Choi, J.H., Jo, H.S., Lim, S., Kim, H.T., Lee, K.W., Moon, K.H., Ha, T., Kwak, S.S., Kim, Y., Lee, E.J. and

Clark, B.S., Stein-O’Brien, G.L., Shiau, F., Cannon, G.H., Davis-Marcisak, E., Sherman, T., Santiago, C.P., Hoang, T.V., Rajaii, F., James-Esposito, R.E. and Gronostajski, R.M., 2019. Single-cell RNA-seq analysis of retinal development identifies NFI factors as regulating mitotic exit and late-born cell specification. Neuron, 102(6), pp.1111-1126.

Cleverley, K., Lee, W.C., Mumford, P., Collins, T., Rickman, M., Cunningham, T.J., Cleak, J., Mianne, J., Szoke-Kovacs, Z., Stewart, M. and Teboul, L., 2021. A novel knockout mouse for the small EDRK-rich factor 2 (Serf2) showing developmental and other deficits. Mammalian Genome, 32, pp.94-103.

Cloos, P.A., Christensen, J., Agger, K. and Helin, K., 2008. Erasing the methyl mark: histone demethylases at the center of cellular differentiation and disease. Genes & development, 22(9), pp.1115-1140.

Cockram, T.O., Puigdellívol, M. and Brown, G.C., 2019. Calreticulin and galectin-3 opsonise bacteria for phagocytosis by microglia. Frontiers in immunology, 10, p.2647.

Cohen, B., Altman, T., Golani-Armon, A., Savulescu, A.F., Ibraheem, A., Mhlanga, M.M., Perlson, E. and Arava, Y.S., 2022. Co-transport of the nuclear-encoded Cox7c mRNA with mitochondria along axons occurs through a coding-region-dependent mechanism. Journal of Cell Science, 135(16), p.jcs259436.

Coleman, K.E., Yin, Y., Lui, S.K.L., Keegan, S., Fenyo, D., Smith, D.J., Rothenberg, E. and Huang, T.T., 2022. USP1-trapping lesions as a source of DNA replication stress and genomic instability. Nature communications, 13(1), p.1740.

Collin, J., Hasoon, M.S., Zerti, D., Hammadi, S., Dorgau, B., Clarke, L., Steel, D., Hussain, R., Coxhead, J., Lisgo, S. and Queen, R., 2023. Single-cell RNA sequencing reveals transcriptional changes of human choroidal and retinal pigment epithelium cells during fetal development, in healthy adult and intermediate age-related macular degeneration. Human molecular genetics, 32(10), pp.1698-1710.

Cui, X.B., Zhang, S.M., Xu, Y.X., Dang, H.W., Liu, C.X., Wang, L.H., Yang, L., Hu, J.M., Liang, W.H., Jiang, J.F. and Li, N., 2016. PFN2, a novel marker of unfavorable prognosis, is a potential therapeutic target involved in esophageal squamous cell carcinoma. Journal of translational medicine, 14, pp.1-16.

Cunningham, O., Gore, M.G. and Mantle, T.J., 2000. Initial-rate kinetics of the flavin reductase reaction catalysed by human biliverdin-IXβ reductase (BVR-B). Biochemical Journal, 345(2), pp.393-399.

Dai, H., Wang, L., Li, L., Huang, Z. and Ye, L., 2021. Metallothionein 1: A new spotlight on inflammatory diseases. Frontiers in immunology, 12, p.739918.

Daldello, E.M., Luong, X.G., Yang, C.R., Kuhn, J. and Conti, M., 2019. Cyclin B2 is required for progression through meiosis in mouse oocytes. Development, 146(8), p.dev172734.

Daniszewski, M., Senabouth, A., Liang, H.H., Han, X., Lidgerwood, G.E., Hernández, D., Sivakumaran, P., Clarke, J.E., Lim, S.Y., Lees, J.G. and Rooney, L., 2022. Retinal ganglion cell-specific genetic regulation in primary open-angle glaucoma. Cell Genomics, 2(6).

Das, R., Koo, M.S., Kim, B.H., Jacob, S.T., Subbian, S., Yao, J., Leng, L., Levy, R., Murchison, C., Burman, W.J. and Moore, C.C., 2013. Macrophage migration inhibitory factor (MIF) is a critical mediator of the innate immune response to Mycobacterium tuberculosis. Proceedings of the National Academy of Sciences, 110(32), pp.E2997-E3006.

Deng, S., Gardner, S.M., Gottlieb, L., Pan, B., Petersson, E.J. and Marmorstein, R., 2023. Molecular role of NAA38 in thermostability and catalytic activity of the human NatC N-terminal acetyltransferase. Structure, 31(2), pp.166-173.

Devi, T.S., Lee, I., Hüttemann, M., Kumar, A., Nantwi, K.D. and Singh, L.P., 2012. TXNIP links innate host defense mechanisms to oxidative stress and inflammation in retinal Muller glia under chronic hyperglycemia: implications for diabetic retinopathy. Journal of Diabetes Research, 2012(1), p.438238.

Diacou, R., Zhao, Y., Zheng, D., Cvekl, A. and Liu, W., 2018. Six3 and Six6 are jointly required for the maintenance of multipotent retinal progenitors through both positive and negative regulation. Cell reports, 25(9), pp.2510-2523.

Diehn, J.J., Diehn, M., Marmor, M.F. and Brown, P.O., 2005. Differential gene expression in anatomical compartments of the human eye. Genome biology, 6, pp.1-13.

Dorgau, B., Collin, J., Rozanska, A., Zerti, D., Unsworth, A., Crosier, M., Hussain, R., Coxhead, J., Dhanaseelan, T., Patel, A. and Sowden, J.C., 2024. Single-cell analyses reveal transient retinal progenitor cells in the ciliary margin of developing human retina. Nature communications, 15(1), p.3567.

Duan, D.R., Pause, A., Burgess, W.H., Aso, T., Chen, D.Y., Garrett, K.P., Conaway, R.C., Conaway, J.W., Linehan, W.M. and Klausner, R.D., 1995. Inhibition of transcription elongation by the VHL tumor suppressor protein. Science, 269(5229), pp.1402-1406.

Duan, R., Du, W. and Guo, W., 2020. EZH2: a novel target for cancer treatment. Journal of hematology & oncology, 13(1), p.104.

Dumax-Vorzet, A., Roboti, P. and High, S., 2013. OST4 is a subunit of the mammalian oligosaccharyltransferase required for efficient N-glycosylation. Journal of cell science, 126(12), pp.2595-2606.

Dunham, E. E., Stevens, E. A., Glover, E., & Bradfield, C. A. (2006). The aryl hydrocarbon receptor signaling pathway is modified through interactions with a Kelch protein. Molecular pharmacology, 70(1), 8–15.

Dupacova, N., Antosova, B., Paces, J. and Kozmik, Z., 2021. Meis homeobox genes control progenitor competence in the retina. Proceedings of the National Academy of Sciences, 118(12), p.e2013136118.

El-Asrar, A.M.A., Mohammad, G., Nawaz, M.I., Abdelsaid, M., Siddiquei, M.M., Alam, K., Van den Eynde, K., De Hertogh, G., Opdenakker, G., Al-Shabrawey, M. and Van Damme, J., 2015. The chemokine platelet factor-4 variant (PF-4var)/CXCL4L1 inhibits diabetes-induced blood–retinal barrier breakdown. Investigative Ophthalmology & Visual Science, 56(3), pp.1956-1964.

Elpidorou, M., Best, S., Poulter, J.A., Hartill, V., Hobson, E., Sheridan, E. and Johnson, C.A., 2021. Novel loss-of-function mutation in HERC2 is associated with severe developmental delay and paediatric lethality. Journal of Medical Genetics, 58(5), pp.334-341.

Emerson, S.E., Stergas, H.R., Bupp‐Chickering, S.O. and Ebert, A.M., 2020. Shootin‐1 is required for nervous system development in zebrafish. Developmental Dynamics, 249(10), pp.1285-1295.

Eskelinen, E.L., 2005. Maturation of autophagic vacuoles in mammalian cells. Autophagy, 1(1), pp.1-10.

Fan, L., Yin, S., Zhang, E. and Hu, H., 2018. Role of p62 in the regulation of cell death induction. Apoptosis, 23, pp.187-193.

Farioli-Vecchioli, S., Micheli, L., Saraulli, D., Ceccarelli, M., Cannas, S., Scardigli, R., Leonardi, L., Cinà, I., Costanzi, M., Ciotti, M.T. and Moreira, P., 2012. Btg1 is required to maintain the pool of stem and progenitor cells of the dentate gyrus and subventricular zone. Frontiers in neuroscience, 6, p.124.

Fei, L. and Xu, H., 2018. Role of MCM2–7 protein phosphorylation in human cancer cells. Cell & bioscience, 8, pp.1-8.

Feng, J., Hu, M., Li, Z., Hu, G., Han, Y., Zhang, Y., Zhang, M. and Ren, J., 2022. Cyclin-Dependent Kinase Subunit 2 (CKS2) as a Prognostic Marker for Stages I–III Invasive Non-Mucinous Lung Adenocarcinoma and Its Role in Affecting Drug Sensitivity. Cells, 11(16), p.2611.

Fernández-Mendívil, C., Luengo, E., Trigo-Alonso, P., García-Magro, N., Negredo, P. and López, M.G., 2021. Protective role of microglial HO-1 blockade in aging: Implication of iron metabolism. Redox biology, 38, p.101789.

Fijalkowska, I., Sharma, D., Bult, C.J. and Danoff, S.K., 2010. Expression of the transcription factor, TFII-I, during post-implantation mouse embryonic development. BMC Research Notes, 3, pp.1-8.

Finkielstein, C.V. and Capelluto, D.G., 2016. Disabled‐2: A modular scaffold protein with multifaceted functions in signaling. Bioessays, 38, pp.S45-S55.

Foster, W.H., Langenbacher, A., Gao, C., Chen, J. and Wang, Y., 2013. Nuclear phosphatase PPM1G in cellular survival and neural development. Developmental Dynamics, 242(9), pp.1101-1109.

Franklin, S., Chen, H., Mitchell-Jordan, S., Ren, S., Wang, Y. and Vondriska, T.M., 2012. Quantitative analysis of the chromatin proteome in disease reveals remodeling principles and identifies high mobility group protein B2 as a regulator of hypertrophic growth. Molecular & cellular proteomics, 11(6).

Freedman, S.J., Sun, Z.Y.J., Kung, A.L., France, D.S., Wagner, G. and Eck, M.J., 2003. Structural basis for negative regulation of hypoxia-inducible factor-1α by CITED2. Nature Structural & Molecular Biology, 10(7), pp.504-512.

Fu, J., Zhang, J., Chen, X., Liu, Z., Yang, X., He, Z., Hao, Y., Liu, B. and Yao, D., 2023. ATPase family AAA domain-containing protein 2 (ATAD2): From an epigenetic modulator to cancer therapeutic target. Theranostics, 13(2), p.787.

Galbraith, K.K., Fujishima, K., Mizuno, H., Lee, S.J., Uemura, T., Sakimura, K., Mishina, M., Watanabe, N. and Kengaku, M., 2018. MTSS1 regulation of actin-nucleating formin DAAM1 in dendritic filopodia determines final dendritic configuration of Purkinje cells. Cell reports, 24(1), pp.95-106.

Gao, W. S., Qu, Y. J., Huai, J., Wei, H., Zhang, Y., & Yue, S. W. 2020. DOK3 is involved in microglial cell activation in neuropathic pain by interacting with GPR84. Aging, 13(1), 389–410.

Gardai, S.J., McPhillips, K.A., Frasch, S.C., Janssen, W.J., Starefeldt, A., Murphy-Ullrich, J.E., Bratton, D.L., Oldenborg, P.A., Michalak, M. and Henson, P.M., 2005. Cell-surface calreticulin initiates clearance of viable or apoptotic cells through trans-activation of LRP on the phagocyte. Cell, 123(2), pp.321-334.

Gemmati, D., Vigliano, M., Burini, F., Mari, R., Hossam Abd El Mohsein, H., Parmeggiani, F. and L Serino, M., 2016. Coagulation factor XIIIA (F13A1): novel perspectives in treatment and pharmacogenetics. Current Pharmaceutical Design, 22(11), pp.1449-1459.

George, M., Ying, G., Rainey, M.A., Solomon, A., Parikh, P.T., Gao, Q., Band, V. and Band, H., 2007. Shared as well as distinct roles of EHD proteins revealed by biochemical and functional comparisons in mammalian cells and C. elegans. BMC cell biology, 8, pp.1-22.

Girotto, G., Scheffer, D.I., Morgan, A., Vozzi, D., Rubinato, E., Di Stazio, M., Muzzi, E., Pensiero, S., Giersch, A.B., Corey, D.P. and Gasparini, P., 2015. PSIP1/LEDGF: a new gene likely involved in sensorineural progressive hearing loss. Scientific reports, 5(1), p.18568.

Glassmann, A., Molly, S., Surchev, L., Nazwar, T.A., Holst, M., Hartmann, W., Baader, S.L., Oberdick, J., Pietsch, T. and Schilling, K., 2007. Developmental expression and differentiation-related neuronspecific splicing of metastasis suppressor 1 (Mtss1) in normal and transformed cerebellar cells. BMC developmental biology, 7, pp.1-15.

Gnana‐Prakasam, J.P., Martin, P.M., Smith, S.B. and Ganapathy, V., 2010. Expression and function of iron‐regulatory proteins in retina. IUBMB life, 62(5), pp.363-370.

Goudreault, Marilyn, Lisa M. D'Ambrosio, Michelle J. Kean, Michael J. Mullin, Brett G. Larsen, Amy Sanchez, Sidharth Chaudhry et al. "A PP2A phosphatase high density interaction network identifies a novel striatin-interacting phosphatase and kinase complex linked to the cerebral cavernous malformation 3 (CCM3) protein." Molecular & Cellular Proteomics 8, no. 1 (2009): 157-171. Grant, B.D. and Caplan, S., 2008. Mechanisms of EHD/RME‐1 protein function in endocytic transport. Traffic, 9(12), pp.2043-2052.

Grasemann, C., Gratias, S., Stephan, H., Schüler, A., Schramm, A., Klein-Hitpass, L., Rieder, H., Schneider, S., Kappes, F., Eggert, A. and Lohmann, D.R., 2005. Gains and overexpression identify DEK and E2F3 as targets of chromosome 6p gains in retinoblastoma. Oncogene, 24(42), pp.6441-6449.

Green, T.D., Park, J., Yin, Q., Fang, S., Crews, A.L., Jones, S.L. and Adler, K.B., 2012. Directed migration of mouse macrophages in vitro involves myristoylated alanine-rich C-kinase substrate (MARCKS) protein. Journal of leukocyte biology, 92(3), pp.633-639.

Greferath U, Goh HC, Chua PY, Åstrand E, O'Brien EE, Fletcher EL, Murphy M. Mapping retinal degeneration and loss-of-function in Rd-FTL mice. Investigative ophthalmology & visual science. 2009 Dec 1;50(12):5955-64.

Greferath, U., Anderson, E.E., Jobling, A.I., Vessey, K.A., Martinez, G., de Iongh, R.U., Kalloniatis, M. and Fletcher, E.L., 2015. Inner retinal change in a novel rd1-FTL mouse model of retinal degeneration. Frontiers in cellular neuroscience, 9, p.293.

Gudipaty, S.A., McNamara, R.P., Morton, E.L. and D'Orso, I., 2015. PPM1G binds 7SK RNA and Hexim1 to block P-TEFb assembly into the 7SK snRNP and sustain transcription elongation. Molecular and cellular biology, 35(22), pp.3810-3828.

Guilherme, A., Soriano, N.A., Furcinitti, P.S. and Czech, M.P., 2004. Role of EHD1 and EHBP1 in perinuclear sorting and insulin-regulated GLUT4 recycling in 3T3-L1 adipocytes. Journal of Biological Chemistry, 279(38), pp.40062-40075.

Guryanova, O.A., Drazba, J.A., Frolova, E.I. and Chumakov, P.M., 2011. Actin cytoskeleton remodeling by the alternatively spliced isoform of PDLIM4/RIL protein. Journal of Biological Chemistry, 286(30), pp.26849-26859.

Hahn P, Lindsten T, Ying GS, Bennett J, Milam AH, Thompson CB, Dunaief JL: Proapoptotic bcl-2 family members, Bax and Bak, are essential for developmental photoreceptor apoptosis. Invest Ophthalmol Vis Sci. 2003, 44: 3598-3605. 10.1167/iovs.02-1113.

Hahn, P., Dentchev, T., Qian, Y., Rouault, T., Harris, Z.L. and Dunaief, J.L., 2004. Immunolocalization and regulation of iron handling proteins ferritin and ferroportin in the retina. Mol Vis, 10, pp.598-607.

Hanschmann, E.M., Godoy, J.R., Berndt, C., Hudemann, C. and Lillig, C.H., 2013. Thioredoxins, glutaredoxins, and peroxiredoxins—molecular mechanisms and health significance: from cofactors to antioxidants to redox signaling. Antioxidants & redox signaling, 19(13), pp.1539-1605.

Hao, H., Kim, D.S., Klocke, B., Johnson, K.R., Cui, K., Gotoh, N., Zang, C., Gregorski, J., Gieser, L., Peng, W. and Fann, Y., 2012. Transcriptional regulation of rod photoreceptor homeostasis revealed by in vivo NRL targetome analysis. PLoS genetics, 8(4), p.e1002649.

Harada, H., Moriya, K., Kobuchi, H., Ishihara, N. and Utsumi, T., 2023. Protein N-myristoylation plays a critical role in the mitochondrial localization of human mitochondrial complex I accessory subunit NDUFB7. Scientific reports, 13(1), p.22991.

Harder, J.M. and Libby, R.T., 2011. BBC3 (PUMA) regulates developmental apoptosis but not axonal injury induced death in the retina. Molecular neurodegeneration, 6, pp.1-10.

Hassan, A., Araguas Rodriguez, P., Heidmann, S.K., Walmsley, E.L., Aughey, G.N. and Southall, T.D., 2020. Condensin I subunit Cap-G is essential for proper gene expression during the maturation of post-mitotic neurons. Elife, 9, p.e55159.

Haynes, S.E., Hollopeter, G., Yang, G., Kurpius, D., Dailey, M.E., Gan, W.B. and Julius, D., 2006. The P2Y12 receptor regulates microglial activation by extracellular nucleotides. Nature neuroscience, 9(12), pp.1512-1519.

He, G.L., Luo, Z., Shen, T.T., Yang, J., Li, P., Luo, X. and Yang, X.S., 2019. Inhibition of HSP90β by ganetespib blocks the microglial signalling of evoked pro-inflammatory responses to heat shock. The International Journal of Biochemistry & Cell Biology, 106, pp.35-45.

He, X., Semenov, M., Tamai, K. and Zeng, X., 2004. LDL receptor-related proteins 5 and 6 in Wnt/βcatenin signaling: arrows point the way.

Heemskerk, V.H., Daemen, M.A. and Buurman, W.A., 1999. Insulin-like growth factor-1 (IGF-1) and growth hormone (GH) in immunity and inflammation. Cytokine & growth factor reviews, 10(1), pp.5-14.

Heinrich, R., Hertz, R., Zemel, E., Mann, I., Brenner, L., Massarweh, A., Berlin, S. and Perlman, I., 2018. ATF3 regulates the expression of AChE during stress. Frontiers in Molecular Neuroscience, 11, p.88.

Helbing, T., Rothweiler, R., Ketterer, E., Goetz, L., Heinke, J., Grundmann, S., Duerschmied, D., Patterson, C., Bode, C. and Moser, M., 2011. BMP activity controlled by BMPER regulates the proinflammatory phenotype of endothelium. Blood, The Journal of the American Society of Hematology, 118(18), pp.5040-5049.

Helferich, A.M., Brockmann, S.J., Reinders, J., Deshpande, D., Holzmann, K., Brenner, D., Andersen, P.M., Petri, S., Thal, D.R., Michaelis, J. and Otto, M., 2018. Dysregulation of a novel miR1825/TBCB/TUBA4A pathway in sporadic and familial ALS. Cellular and Molecular Life Sciences, 75, pp.4301-4319.

Héraud, C., Pinault, M., Lagrée, V., & Moreau, V. (2019). p190RhoGAPs, the ARHGAP35- and ARHGAP5-Encoded Proteins, in Health and Disease. Cells, 8(4), 351.

Hess, J., Angel, P. and Schorpp-Kistner, M., 2004. AP-1 subunits: quarrel and harmony among siblings. Journal of cell science, 117(25), pp.5965-5973.

Hoi, A.Y., Hickey, M.J., Hall, P., Yamana, J., O’Sullivan, K.M., Santos, L.L., James, W.G., Kitching, A.R. and Morand, E.F., 2006. Macrophage migration inhibitory factor deficiency attenuates macrophage recruitment, glomerulonephritis, and lethality in MRL/lpr mice. The Journal of Immunology, 177(8), pp.5687-5696.

Hoozemans, J.J., Van Haastert, E.S., Nijholt, D.A., Rozemuller, A.J. and Scheper, W., 2012. Activation of the unfolded protein response is an early event in Alzheimer’s and Parkinson’s disease. Neurodegenerative Diseases, 10(1-4), pp.212-215.

Horazdovsky, B.F., Davies, B.A., Seaman, M.N., McLaughlin, S.A., Yoon, S.H. and Emr, S.D., 1997. A sorting nexin-1 homologue, Vps5p, forms a complex with Vps17p and is required for recycling the vacuolar protein-sorting receptor. Molecular biology of the cell, 8(8), pp.1529-1541.

Hsing, L.C. and Rudensky, A.Y., 2005. The lysosomal cysteine proteases in MHC class II antigen presentation. Immunological reviews, 207(1), pp.229-241.

Huang, C. Y., Chen, J. J., Wu, J. S., Tsai, H. D., Lin, H., Yan, Y. T., et al. (2015). Novel link of antiapoptotic ATF3 with pro-apoptotic CTMP in the ischemic brain. Mol. Neurobiol. 51, 543–557. doi: 10.1007/s12035-014-8710-0

Huang, G., Li, H. and Zhang, H., 2020. Abnormal expression of mitochondrial ribosomal proteins and their encoding genes with cell apoptosis and diseases. International journal of molecular sciences, 21(22), p.8879.

Huang, Y.G., Li, D., Wang, L., Su, X.M. and Tang, X.B., 2022. CENPF/CDK1 signaling pathway enhances the progression of adrenocortical carcinoma by regulating the G2/M-phase cell cycle. Journal of translational medicine, 20(1), p.78.

Hughes S, Edwards JK, Wilcox AG, Pothecary CA, Barnard AR, Joynson R, Joynson G, Hankins MW, Peirson SN, Banks G, Nolan PM. Zfhx3 modulates retinal sensitivity and circadian responses to light. FASEB J. 2021 Sep;35(9):e21802.

Hunt, D., Hossain-Ibrahim, K., Mason, M. R. J., Coffin, R. S., Lieberman, A. R., Winterbottom, J. A., et al. (2004). ATF3 upregulation in glia during Wallerian degeneration: differential expression in peripheral nerves and CNS white matter. BMC Neurosci. 5:9. doi: 10.1186/1471-2202-5-9.

Inoue, Y., Shimazawa, M., Noda, Y., Nagano, R., Otsuka, T., Kuse, Y., Nakano, Y., Tsuruma, K., Nakagami, Y. and Hara, H., 2017. RS 9, a novel Nrf2 activator, attenuates light‐induced death of cells of photoreceptor cells and Müller glia cells. Journal of neurochemistry, 141(5), pp.750-765.

Ishibashi, Y., Maita, H., Yano, M., Koike, N., Tamai, K., Ariga, H. and Iguchi-Ariga, S.M., 2001. Pim-1 translocates sorting nexin 6/TRAF4-associated factor 2 from cytoplasm to nucleus. FEBS letters, 506(1), pp.33-38.

Iwagawa, T., Aihara, Y., Umutoni, D., Baba, Y., Murakami, A., Miyado, K. and Watanabe, S., 2020. Cd9 protects photoreceptors from injury and potentiates Edn2 expression. Investigative Ophthalmology & Visual Science, 61(3), pp.7-7.

Iwamoto T., Mamiya N., Masushige S., and Kida S., PLCγ2 activates CREB-dependent transcription in PC12 cells through phosphorylation of CREB at serine 133, Cytotechnology. (2005) 47, no. 1–3, 107– 116

Janz, R., Südhof, T.C., Hammer, R.E., Unni, V., Siegelbaum, S.A. and Bolshakov, V.Y., 1999. Essential roles in synaptic plasticity for synaptogyrin I and synaptophysin I. Neuron, 24(3), pp.687-700.

Jaykumar, A.B., Plumber, S., Barry, D.M., Binns, D., Wichaidit, C., Grzemska, M., Earnest, S., Goldsmith, E.J., Cleaver, O. and Cobb, M.H., 2022. WNK1 collaborates with TGF-β in endothelial cell junction turnover and angiogenesis. Proceedings of the National Academy of Sciences, 119(30), p.e2203743119.

Jeon, C.J., Strettoi, E. and Masland, R.H., 1998. The major cell populations of the mouse retina. Journal of Neuroscience, 18(21), pp.8936-8946.

Jeon, S.B., Yoon, H.J., Chang, C.Y., Koh, H.S., Jeon, S.H. and Park, E.J., 2010. Galectin-3 exerts cytokine-like regulatory actions through the JAK–STAT pathway. The Journal of Immunology, 185(11), pp.7037-7046.

Jia, L., Oh, E.C., Ng, L., Srinivas, M., Brooks, M., Swaroop, A. and Forrest, D., 2009. Retinoid-related orphan nuclear receptor RORβ is an early-acting factor in rod photoreceptor development. Proceedings of the National Academy of Sciences, 106(41), pp.17534-17539.

Jia, Y., Song, T., Wei, C., Ni, C., Zheng, Z., Xu, Q., Ma, H., Li, L., Zhang, Y., He, X. and Xu, Y., 2009. Negative regulation of MAVS-mediated innate immune response by PSMA7. The Journal of Immunology, 183(7), pp.4241-4248.

Jiang, H.R., Al Rasebi, Z., Mensah-Brown, E., Shahin, A., Xu, D., Goodyear, C.S., Fukada, S.Y., Liu, F.T., Liew, F.Y. and Lukic, M.L., 2009. Galectin-3 deficiency reduces the severity of experimental autoimmune encephalomyelitis. The Journal of Immunology, 182(2), pp.1167-1173.

Jiang, J., Chan, Y.S., Loh, Y.H., Cai, J., Tong, G.Q., Lim, C.A., Robson, P., Zhong, S. and Ng, H.H., 2008. A core Klf circuitry regulates self-renewal of embryonic stem cells. Nature cell biology, 10(3), pp.353360.

Jiang, S.M., Zeng, L.P., Zeng, J.H., Tang, L., Chen, X.M. and Wei, X., 2015. β-III-Tubulin: a reliable marker for retinal ganglion cell labeling in experimental models of glaucoma. International journal of ophthalmology, 8(4), p.643.

Jiang, Y., Ding, Q., Xie, X., Libby, R.T., Lefebvre, V. and Gan, L., 2013. Transcription factors SOX4 and SOX11 function redundantly to regulate the development of mouse retinal ganglion cells. Journal of Biological Chemistry, 288(25), pp.18429-18438.

Jing, Z., Jia‐jun, W. and Wei‐jie, Y., 2020. Phosphorylation of Dab2 is involved in inhibited VEGF-VEGFR‐2 signaling induced by downregulation of syndecan‐1 in glomerular endothelial cell. Cell Biology International, 44(3), pp.894-904.

Joe, C.O., 2018. mTORC1 accelerates retinal development via the immunoproteasome. Nature Communications, 9(1), p.2502.

Johnson, L.A., Banerji, S., Lawrance, W., Gileadi, U., Prota, G., Holder, K.A., Roshorm, Y.M., Hanke, T., Cerundolo, V., Gale, N.W. and Jackson, D.G., 2017. Dendritic cells enter lymph vessels by hyaluronanmediated docking to the endothelial receptor LYVE-1. Nature immunology, 18(7), pp.762-770.

Jovic, M., Naslavsky, N., Rapaport, D., Horowitz, M. and Caplan, S., 2007. EHD1 regulates β1 integrin endosomal transport: effects on focal adhesions, cell spreading and migration. Journal of cell science, 120(5), pp.802-814.

Kamura, T., Sato, S., Haque, D., Liu, L., Kaelin, W.G., Conaway, R.C. and Conaway, J.W., 1998. The Elongin BC complex interacts with the conserved SOCS-box motif present in members of the SOCS, ras, WD-40 repeat, and ankyrin repeat families. Genes & development, 12(24), pp.3872-3881.

Kanda A, Hirose I, Noda K, Murata M, Ishida S., 2020. Glucocorticoid-transactivated TSC22D3 attenuates hypoxia- and diabetes-induced Müller glial galectin-1 expression via HIF-1α destabilization. J Cell Mol Med; 24(8): 4589–4599.

Kanda, A., Noda, K., Saito, W. and Ishida, S., 2015. Aflibercept traps galectin-1, an angiogenic factor associated with diabetic retinopathy. Scientific reports, 5(1), p.17946.

Kanwar, M. and Kowluru, R.A., 2009. Role of glyceraldehyde 3-phosphate dehydrogenase in the development and progression of diabetic retinopathy. Diabetes, 58(1), pp.227-234.

Kappes, F., Scholten, I., Richter, N., Gruss, C. and Waldmann, T., 2004. Functional domains of the ubiquitous chromatin protein DEK. Molecular and cellular biology, 24(13), pp.6000-6010.

Karlstetter, M., Nothdurfter, C., Aslanidis, A., Moeller, K., Horn, F., Scholz, R., Neumann, H., Weber, B.H., Rupprecht, R. and Langmann, T., 2014. Translocator protein (18 kDa)(TSPO) is expressed in reactive retinal microglia and modulates microglial inflammation and phagocytosis. Journal of neuroinflammation, 11, pp.1-13.

Karplus PA., 2014. A primer on peroxiredoxin biochemistry. Free Radic Biol Med 80:183–190

Kasahara, E., Lin, L.R., Ho, Y.S. and Reddy, V.N., 2005. SOD2 protects against oxidation-induced apoptosis in mouse retinal pigment epithelium: implications for age-related macular degeneration. Investigative ophthalmology & visual science, 46(9), pp.3426-3434.

Kaufman, M.L., Goodson, N.B., Park, K.U., Schwanke, M., Office, E., Schneider, S.R., Abraham, J., Hensley, A., Jones, K.L. and Brzezinski, J.A., 2021. Initiation of Otx2 expression in the developing mouse retina requires a unique enhancer and either Ascl1 or Neurog2 activity. Development, 148(12), p.dev199399.

Kim, J.H., Lee, J.Y., Lee, K.T., Lee, J.K., Lee, K.H., Jang, K.T., Heo, J.S., Choi, S.H. and Rhee, J.C., 2010. RGS16 and FosB underexpressed in pancreatic cancer with lymph node metastasis promote tumor progression. Tumor Biology, 31, pp.541-548.

Kim, S.G., Lee, S., Kim, Y., Park, J., Woo, D., Kim, D., Li, Y., Shin, W., Kang, H., Yook, C. and Lee, M., 2021. Tanc2-mediated mTOR inhibition balances mTORC1/2 signaling in the developing mouse brain and human neurons. Nature communications, 12(1), p.2695.

Kimura, H., Takizawa, N., Allemand, E., Hori, T., Iborra, F.J., Nozaki, N., Muraki, M., Hagiwara, M., Krainer, A.R., Fukagawa, T. and Okawa, K., 2006. A novel histone exchange factor, protein phosphatase 2Cγ, mediates the exchange and dephosphorylation of H2A–H2B. The Journal of cell biology, 175(3), pp.389-400.

Kirschke, H., Wiederanders, B., Brömme, D. and Rinne, A., 1989. Cathepsin S from bovine spleen. Purification, distribution, intracellular localization and action on proteins. Biochemical Journal, 264(2), pp.467-473.

Klein-Soyer, C., Azorsa, D.O., Cazenave, J.P. and Lanza, F., 2000. CD9 participates in endothelial cell migration during in vitro wound repair. Arteriosclerosis, thrombosis, and vascular biology, 20(2), pp.360-369.

Kole, C., Brommer, B., Nakaya, N., Sengupta, M., Bonet-Ponce, L., Zhao, T., Wang, C., Li, W., He, Z. and Tomarev, S., 2020. Activating transcription factor 3 (ATF3) protects retinal ganglion cells and promotes functional preservation after optic nerve crush. Investigative Ophthalmology & Visual Science, 61(2), pp.31-31.

Kolosova, N.G., Kozhevnikova, O.S., Telegina, D.V., Fursova, A.Z., Stefanova, N.A., Muraleva, N.A., Venanzi, F., Sherman, M.Y., Kolesnikov, S.I., Sufianov, A.A. and Gabai, V.L., 2018. p62/SQSTM1 coding plasmid prevents age related macular degeneration in a rat model. Aging (Albany NY), 10(8), p.2136.

Kondrashov, N., Pusic, A., Stumpf, C.R., Shimizu, K., Hsieh, A.C., Xue, S., Ishijima, J., Shiroishi, T. and Barna, M., 2011. Ribosome-mediated specificity in Hox mRNA translation and vertebrate tissue patterning. Cell, 145(3), pp.383-397.

Königs, V., de Oliveira Freitas Machado, C., Arnold, B., Blümel, N., Solovyeva, A., Löbbert, S.,

Koso, H., Tsuhako, A., Lai, C.Y., Baba, Y., Otsu, M., Ueno, K., Nagasaki, M., Suzuki, Y. and Watanabe, S., 2016. Conditional rod photoreceptor ablation reveals Sall1 as a microglial marker and regulator of microglial morphology in the retina. Glia, 64(11), pp.2005-2024.

Kotoula, V., Krikelis, D., Karavasilis, V., Koletsa, T., Eleftheraki, A.G., Televantou, D., Christodoulou, C., Dimoudis, S., Korantzis, I., Pectasides, D. and Syrigos, K.N., 2012. Expression of DNA repair and replication genes in non-small cell lung cancer (NSCLC): a role for thymidylate synthetase (TYMS). BMC cancer, 12, pp.1-15.

Kramer, L., Turk, D. and Turk, B., 2017. The future of cysteine cathepsins in disease management. Trends in pharmacological sciences, 38(10), pp.873-898.

Krysko, D.V., Ravichandran, K.S. and Vandenabeele, P., 2018. Macrophages regulate the clearance of living cells by calreticulin. Nature communications, 9(1), p.4644.

Kumar, N., Mondal, M., Arathi, B.P., Sundaresan, N.R. and Somasundaram, K., 2023. Histone acetyltransferase 1 (HAT1) acetylates hypoxia-inducible factor 2 alpha (HIF2A) to execute hypoxia response. Biochimica et Biophysica Acta (BBA)-Gene Regulatory Mechanisms, 1866(1), p.194900.

Kumar, R., Mani, A.M., Singh, N.K. and Rao, G.N., 2020. PKCθ-JunB axis via upregulation of VEGFR3 expression mediates hypoxia-induced pathological retinal neovascularization. Cell Death & Disease, 11(5), p.325.

Kwon, S.J., Lee, G.T., Lee, J.H., Iwakura, Y., Kim, W.J. and Kim, I.Y., 2014. Mechanism of protumorigenic effect of BMP‐6: Neovascularization involving tumor‐associated macrophages and IL1α. The Prostate, 74(2), pp.121-133.

Labandeira-Garcia, J.L., Costa-Besada, M.A., Labandeira, C.M., Villar-Cheda, B. and Rodríguez-Perez, A.I., 2017. Insulin-like growth factor-1 and neuroinflammation. Frontiers in aging neuroscience, 9, p.365.

Laux, T., Fukami, K., Thelen, M., Golub, T., Frey, D. and Caroni, P., 2000. GAP43, MARCKS, and CAP23 modulate PI (4, 5) P2 at plasmalemmal rafts, and regulate cell cortex actin dynamics through a common mechanism. The Journal of cell biology, 149(7), pp.1455-1472.

Lavin Y, Winter D, Blecher-Gonen R, David E, Keren-Shaul H, Merad M, Jung S, Amit I. 2014. Tissueresident macrophage enhancer landscapes are shaped by the local microenvironment. Cell 159: 1312–1326.

Lee, H.S., Park, Y.Y., Cho, M.Y., Chae, S., Yoo, Y.S., Kwon, M.H., Lee, C.W. and Cho, H., 2015. The chromatin remodeller RSF1 is essential for PLK1 deposition and function at mitotic kinetochores. Nature communications, 6(1), p.7904.

Lee, S.J., Wang, W., Jin, L., Lu, X., Gao, L., Chen, Y., Liu, T., Emery, D., Vukmanic, E., Liu, Y. and Kaplan, H.J., 2021. Rod photoreceptor clearance due to misfolded rhodopsin is linked to a DAMP-immune checkpoint switch. Journal of Biological Chemistry, 296.

Leimbacher, P.A., Jones, S.E., Shorrocks, A.M.K., de Marco Zompit, M., Day, M., Blaauwendraad, J., Bundschuh, D., Bonham, S., Fischer, R., Fink, D. and Kessler, B.M., 2019. MDC1 interacts with TOPBP1 to maintain chromosomal stability during mitosis. Molecular cell, 74(3), pp.571-583.

Léveillé, F., Papadia, S., Fricker, M., Bell, K.F., Soriano, F.X., Martel, M.A., Puddifoot, C., Habel, M., Wyllie, D.J., Ikonomidou, C. and Tolkovsky, A.M., 2010. Suppression of the intrinsic apoptosis pathway by synaptic activity. Journal of Neuroscience, 30(7), pp.2623-2635.

Li, H., Liu, B., Lian, L., Zhou, J., Xiang, S., Zhai, Y., Chen, Y., Ma, X., Wu, W. and Hou, L., 2021. High dose expression of heme oxigenase-1 induces retinal degeneration through ER stress-related DDIT3. Molecular neurodegeneration, 16, pp.1-17.

Li, J., Parker, B., Martyn, C., Natarajan, C. and Guo, J., 2013. The PMP22 gene and its related diseases. Molecular neurobiology, 47, pp.673-698.

Li, L.D., Sun, H.F., Liu, X.X., Gao, S.P., Jiang, H.L., Hu, X. and Jin, W., 2015. Down-regulation of NDUFB9 promotes breast cancer cell proliferation, metastasis by mediating mitochondrial metabolism. PloS one, 10(12), p.e0144441.

Liang, Q., Wu, N., Zaneveld, S., Liu, H., Fu, S., Wang, K., Bertrand, R., Wang, J., Li, Y. and Chen, R., 2021. Transcript isoforms of Reep6 have distinct functions in the retina. Human Molecular Genetics, 30(21), pp.1907-1918.

Liao, H., Ji, F. and Ying, S., 2017. CDK1: beyond cell cycle regulation. Aging (Albany NY), 9(12), p.2465.

Lidgerwood, G.E., Senabouth, A., Smith-Anttila, C.J., Gnanasambandapillai, V., Kaczorowski, D.C., Amann-Zalcenstein, D., Fletcher, E.L., Naik, S.H., Hewitt, A.W., Powell, J.E. and Pébay, A., 2021. Transcriptomic profiling of human pluripotent stem cell-derived retinal pigment epithelium over time. Genomics, Proteomics and Bioinformatics, 19(2), pp.223-242.

Lin, D.I., Aggarwal, P. and Diehl, J.A., 2008. Phosphorylation of MCM3 on Ser-112 regulates its incorporation into the MCM2–7 complex. Proceedings of the National Academy of Sciences, 105(23), pp.8079-8084.

Lin, S.X., Grant, B., Hirsh, D. and Maxfield, F.R., 2001. Rme-1 regulates the distribution and function of the endocytic recycling compartment in mammalian cells. Nature cell biology, 3(6), pp.567-572.

Lipski, D.A., Foucart, V., Dewispelaere, R., Caspers, L.E., Defrance, M., Bruyns, C. and Willermain, F., 2020. Retinal endothelial cell phenotypic modifications during experimental autoimmune uveitis: a transcriptomic approach. BMC ophthalmology, 20, pp.1-25.

Liu, C., Tian, G., Tu, Y., Fu, J., Lan, C. and Wu, N., 2009. Expression pattern and clinical prognostic relevance of bone morphogenetic protein-2 in human gliomas. Japanese journal of clinical oncology, 39(10), pp.625-631.

Liu, G., Li, F., Chen, M., Luo, Y., Dai, Y. and Hou, P., 2022. SNRPD1/E/F/G serve as potential prognostic biomarkers in lung adenocarcinoma. Frontiers in Genetics, 13, p.813285.

Liu, H., Etter, P., Hayes, S., Jones, I., Nelson, B., Hartman, B., Forrest, D. and Reh, T.A., 2008. NeuroD1 regulates expression of thyroid hormone receptor β2 and cone opsins in the developing mouse retina. Journal of Neuroscience, 28(3), pp.749-756.

Liu, J., Stevens, P.D., Eshleman, N.E. and Gao, T., 2013. Protein phosphatase PPM1G regulates protein translation and cell growth by dephosphorylating 4E binding protein 1 (4E-BP1). Journal of Biological Chemistry, 288(32), pp.23225-23233.

Liu, Q., Liu, H., Li, L., Dong, X., Ru, X., Fan, X., Wen, T. and Liu, J., 2019. ATAD2 predicts poor outcomes in patients with ovarian cancer and is a marker of proliferation. International Journal of Oncology, 56(1), pp.219-231.

Liu, X., Yuan, M., Xiang, Q., Li, Z., Xu, F., Chen, W., Chen, J., Huang, J., Yu, N., Zhou, Z. and Long, X., 2022. Single-cell RNA sequencing of subcutaneous adipose tissues identifies therapeutic targets for cancer-associated lymphedema. Cell Discovery, 8(1), p.58.

Liu, Y., Ji, W., Shergalis, A., Xu, J., Delaney, A.M., Calcaterra, A., Pal, A., Ljungman, M., Neamati, N. and Rehemtulla, A., 2019. Activation of the unfolded protein response via inhibition of protein disulfide isomerase decreases the capacity for DNA repair to sensitize glioblastoma to radiotherapy. Cancer research, 79(11), pp.2923-2932.

Lloyd-Burton SM, York EM, Anwar MA, Vincent AJ, Roskams AJ. SPARC regulates microgliosis and functional recovery following cortical ischemia. J Neurosci. 2013 Mar 6;33(10):4468-81.

Lok, C.N., Lai, Y.T. and Fung, Y.M., 2019. Biliverdin reductase b/flavin reductase (blvrb/flr) exhibits heme-regulated, nadph-dependent reductase activity and confers cytoprotection in developing erythroid cells. Experimental Hematology, 76, p.S76.

Lou H, Komata M, Katou Y, Guan Z, Reis CC, Budd M, Shirahige K, Campbell JL. Mrc1 and DNA polymerase epsilon function together in linking DNA replication and the S phase checkpoint. Molecular Cell. 2008.

Lu Y, Ye Y, Yang Q, Shi S. Single-cell RNA-sequence analysis of mouse glomerular mesangial cells uncovers mesangial cell essential genes. Kidney Int. 2017 Aug;92(2):504-513. doi: 10.1016/j.kint.2017.01.016. Epub 2017 Mar 18. PMID: 28320530.

Lupo, R., Breiling, A., Bianchi, M.E. and Orlando, V., 2001. Drosophila chromosome condensation proteins Topoisomerase II and Barren colocalize with Polycomb and maintain Fab-7 PRE silencing. Molecular cell, 7(1), pp.127-136.

Luscan, R., Mechaussier, S., Paul, A., Tian, G., Gérard, X., Defoort-Dellhemmes, S., Loundon, N., Audo, I., Bonnin, S., LeGargasson, J.F. and Dumont, J., 2017. Mutations in TUBB4B cause a distinctive sensorineural disease. The American Journal of Human Genetics, 101(6), pp.1006-1012.

Luz-Madrigal, A., Grajales-Esquivel, E., Tangeman, J., Kosse, S., Liu, L., Wang, K., Fausey, A., Liang, C., Tsonis, P.A. and Del Rio-Tsonis, K., 2020. DNA demethylation is a driver for chick retina regeneration. Epigenetics, 15(9), pp.998-1019.

Lyu, Y.L. and Wang, J.C., 2003. Aberrant lamination in the cerebral cortex of mouse embryos lacking DNA topoisomerase IIβ. Proceedings of the National Academy of Sciences, 100(12), pp.7123-7128.

MacDonald BT, He X. Frizzled and LRP5/6 receptors for Wnt/β-catenin signaling. Cold Spring Harbor perspectives in biology. 2012 Dec 1;4(12):a007880.

Maghzal, G.J., Thomas, S.R., Hunt, N.H. and Stocker, R., 2008. Cytochrome b5, not superoxide anion radical, is a major reductant of indoleamine 2, 3-dioxygenase in human cells. Journal of Biological Chemistry, 283(18), pp.12014-12025.

Makhina, T., Loers, G., Schulze, C., Ueberle, B., Schachner, M. and Kleene, R., 2009. Extracellular GAPDH binds to L1 and enhances neurite outgrowth. Molecular and Cellular Neuroscience, 41(2), pp.206-218.

Maret, W. and Krężel, A., 2007. Cellular zinc and redox buffering capacity of metallothionein/thionein in health and disease. Molecular medicine, 13, pp.371-375.

Martins, I., Kepp, O., Galluzzi, L., Senovilla, L., Schlemmer, F., Adjemian, S., Menger, L., Michaud, M., Zitvogel, L. and Kroemer, G., 2010. Surface‐exposed calreticulin in the interaction between dying cells and phagocytes. Annals of the New York Academy of Sciences, 1209(1), pp.77-82.

Matheson, S.F.; Hu, K.Q.; Brouns, M.R.; Sordella, R.; VanderHeide, J.D.; Settleman, J. Distinct but overlapping functions for the closely related p190 RhoGAPs in neural development. Dev. Neurosci. 2006, 28, 538–550.

Matic, L.P., Jesus Iglesias, M., Vesterlund, M., Lengquist, M., Hong, M.G., Saieed, S., SanchezRivera, L., Berg, M., Razuvaev, A., Kronqvist, M. and Lund, K., 2018. Novel multiomics profiling of human carotid atherosclerotic plaques and plasma reveals biliverdin reductase B as a marker of intraplaque hemorrhage. JACC: Basic to Translational Science, 3(4), pp.464-480.

Matsuda, A., Kotake, S., Tagawa, Y., Matsuda, H. and Nishihira, J., 1996. Detection and immunolocalization of macrophage migration inhibitory factor in rat iris and ciliary epithelium. Immunology letters, 53(1), pp.1-5.

Matsuda, A., Tagawa, Y., Yoshida, K., Matsuda, H. and Nishihira, J., 1997. Expression of macrophage migration inhibitory factor in rat retina and its immunohistochemical localization. Journal of neuroimmunology, 77(1), pp.85-90.

Mayer, C., Hafemeister, C., Bandler, R.C., Machold, R., Batista Brito, R., Jaglin, X., Allaway, K., Butler, A., Fishell, G. and Satija, R., 2018. Developmental diversification of cortical inhibitory interneurons. Nature, 555(7697), pp.457-462.

McClure, A. W., & Diffley, J. F. 2021. Rad53 checkpoint kinase regulation of DNA replication fork rate via Mrc1 phosphorylation. eLife, 10, e69726.

McConnachie, L.A., Mohar, I., Hudson, F.N., Ware, C.B., Ladiges, W.C., Fernandez, C., ChattertonKirchmeier, S., White, C.C., Pierce, R.H. and Kavanagh, T.J., 2007. Glutamate cysteine ligase modifier subunit deficiency and gender as determinants of acetaminophen-induced hepatotoxicity in mice. Toxicological Sciences, 99(2), pp.628-636.

Mehta, A. and Orchard, S., 2009. Nucleoside diphosphate kinase (NDPK, NM23, AWD): recent regulatory advances in endocytosis, metastasis, psoriasis, insulin release, fetal erythroid lineage and heart failure; translational medicine exemplified. Molecular and cellular biochemistry, 329, pp.3-15.

Mencarelli, C., Nitarska, J., Kroecher, T., Ferraro, F., Massey, K., Riccio, A. and Pichaud, F., 2018. RanBP1 couples nuclear export and Golgi regulation through LKB1 to promote cortical neuron polarity. Cell reports, 24(10), pp.2529-2539.

Menon, M., Mohammadi, S., Davila-Velderrain, J., Goods, B.A., Cadwell, T.D., Xing, Y., Stemmer-Rachamimov, A., Shalek, A.K., Love, J.C., Kellis, M. and Hafler, B.P., 2019. Single-cell transcriptomic atlas of the human retina identifies cell types associated with age-related macular degeneration. Nature communications, 10(1), p.4902.

Merritt II, J.L. and Gallagher, R.C., 2018. Inborn errors of carbohydrate, ammonia, amino acid, and organic acid metabolism. In Avery's Diseases of the Newborn (pp. 230-252). Elsevier.

Meyer, N., Zielke, S., Michaelis, J.B., Linder, B., Warnsmann, V., Rakel, S., Osiewacz, H.D., Fulda, S., Mittelbronn, M., Münch, C. and Behrends, C., 2018. AT 101 induces early mitochondrial dysfunction and HMOX1 (heme oxygenase 1) to trigger mitophagic cell death in glioma cells. Autophagy, 14(10), pp.1693-1709.

Michel, H., Behr, J., Harrenga, A. and Kannt, A., 1998. Cytochrome c oxidase: structure and spectroscopy. Annual review of biophysics and biomolecular structure, 27(1), pp.329-356.

Milenkovic, I. and Novoa, E.M., 2025. Ribosomal protein paralogues in ribosome specialization. Philosophical Transactions B, 380(1921), p.20230387.

Min, S., Choi, Y.W., Yun, H., Jo, S., Ji, J.H. and Cho, H., 2018. Post-translational regulation of the RSF1 chromatin remodeler under DNA damage. Molecules and cells, 41(2), pp.127-133.

Moore, D.L., Blackmore, M.G., Hu, Y., Kaestner, K.H., Bixby, J.L., Lemmon, V.P. and Goldberg, J.L., 2009. KLF family members regulate intrinsic axon regeneration ability. Science, 326(5950), pp.298301.

Morciano, G., Pedriali, G., Bonora, M., Pavasini, R., Mikus, E., Calvi, S., Bovolenta, M., LebiedzinskaArciszewska, M., Pinotti, M., Albertini, A. and Wieckowski, M.R., 2021. A naturally occurring mutation in ATP synthase subunit c is associated with increased damage following hypoxia/reoxygenation in STEMI patients. Cell Reports, 35(2).

Morrell, N.W., Bloch, D.B., Ten Dijke, P., Goumans, M.J.T., Hata, A., Smith, J., Yu, P.B. and Bloch, K.D., 2016. Targeting BMP signalling in cardiovascular disease and anaemia. Nature Reviews Cardiology, 13(2), pp.106-120.

Morrow, E.M., Furukawa, T., Lee, J.E. and Cepko, C.L., 1999. NeuroD regulates multiple functions in the developing neural retina in rodent. Development, 126(1), pp.23-36.

Müller, S., Ronfani, L. and Bianchi, M.E., 2004. Regulated expression and subcellular localization of HMGB1, a chromatin protein with a cytokine function. Journal of internal medicine, 255(3), pp.332343.

Müller-McNicoll, M., Botti, V., de Jesus Domingues, A.M., Brandl, H., Schwich, O.D., Steiner, M.C., Curk, T., Poser, I., Zarnack, K. and Neugebauer, K.M., 2016. SR proteins are NXF1 adaptors that link alternative RNA processing to mRNA export. Genes & development, 30(5), pp.553-566.

Murakami-Tonami, Y., Kishida, S., Takeuchi, I., Katou, Y., Maris, J.M., Ichikawa, H., Kondo, Y., Sekido, Y., Shirahige, K., Murakami, H. and Kadomatsu, K., 2014. Inactivation of SMC2 shows a synergistic lethal response in MYCN-amplified neuroblastoma cells. Cell cycle, 13(7), pp.11151131.

Murray, M.V., Kobayashi, R. and Krainer, A.R., 1999. The type 2C Ser/Thr phosphatase PP2Cγ is a pre-mRNA splicing factor. Genes & development, 13(1), pp.87-97.

Musio, A., 2020. The multiple facets of the SMC1A gene. Gene, 743, p.144612.

Myant, K. and Stancheva, I., 2008. LSH cooperates with DNA methyltransferases to repress transcription. Molecular and cellular biology.

Nakazawa, T., Hisatomi, T., Nakazawa, C., Noda, K., Maruyama, K., She, H., Matsubara, A., Miyahara, S., Nakao, S., Yin, Y. and Benowitz, L., 2007. Monocyte chemoattractant protein 1 mediates retinal detachment-induced photoreceptor apoptosis. Proceedings of the National Academy of Sciences, 104(7), pp.2425-2430.

Namekata, K., Guo, X., Kimura, A., Azuchi, Y., Kitamura, Y., Harada, C. and Harada, T., 2020. Roles of the DOCK-D family proteins in a mouse model of neuroinflammation. Journal of Biological Chemistry, 295(19), pp.6710-6720.

Nasaré, A.M., Tedesco, R.C., Cristovam, P.C., Cenedese, M.A., Galisteo Jr, A.J., Andrade Jr, H.F., Gomes, J.Á.P., Guimarães, É.V., Barbosa, H.S. and Alonso, L.G., 2015. Toxoplasma gondii infection of chicken embryos causes retinal changes and modulates HSP90B1 gene expression: a promising ocular toxoplasmosis model. European Journal of Microbiology and Immunology, 5(4), pp.316-320.

Nasiri, E., Sankowski, R., Dietrich, H., Oikonomidi, A., Huerta, P.T., Popp, J., Al-Abed, Y. and Bacher, M., 2020. Key role of MIF-related neuroinflammation in neurodegeneration and cognitive impairment in Alzheimer’s disease. Molecular Medicine, 26, pp.1-12.

Nasrabadi, D., Rezaeiani, S., Eslaminejad, M.B. and Shabani, A., 2018. Improved protocol for chondrogenic differentiation of bone marrow derived mesenchymal stem cells-effect of PTHrP and FGF-2 on TGFβ1/BMP2-induced chondrocytes hypertrophy. Stem Cell Reviews and Reports, 14, pp.755-766.

Navneet, S., Zhao, J., Wang, J., Mysona, B., Barwick, S., Kaidery, N.A., Saul, A., Kaddour-Djebbar, I., Bollag, W.B., Thomas, B. and Bollinger, K.E., 2019. Hyperhomocysteinemia-induced death of retinal ganglion cells: The role of Müller glial cells and NRF2. Redox biology, 24, p.101199.

Neves, J., Zhu, J., Sousa-Victor, P., Konjikusic, M., Riley, R., Chew, S., Qi, Y., Jasper, H. and Lamba, D.A., 2016. Immune modulation by MANF promotes tissue repair and regenerative success in the retina. Science, 353(6294), p.aaf3646.

Nicholls, T.J., Spåhr, H., Jiang, S., Siira, S.J., Koolmeister, C., Sharma, S., Kauppila, J.H., Jiang, M., Kaever, V., Rackham, O. and Chabes, A., 2019. Dinucleotide degradation by REXO2 maintains promoter specificity in mammalian mitochondria. Molecular cell, 76(5), pp.784-796.

Nishida, A., Furukawa, A., Koike, C., Tano, Y., Aizawa, S., Matsuo, I. and Furukawa, T., 2003. Otx2 homeobox gene controls retinal photoreceptor cell fate and pineal gland development. Nature neuroscience, 6(12), pp.1255-1263.

Nishihira, J., 2012. Molecular function of macrophage migration inhibitory factor and a novel therapy for inflammatory bowel disease. Annals of the New York Academy of Sciences, 1271(1), pp.53-57.

Nishikawa, H. and Suzuki, H., 2018. Possible role of inflammation and galectin-3 in brain injury after subarachnoid hemorrhage. Brain sciences, 8(2), p.30.

Nishinakamura R, Matsumoto Y, Nakao K, Nakamura K, Sato A, Copeland NG, Gilbert DJ, Jenkins NA, Scully S, Lacey DL, Katsuki M, Asashima M, Yokota T. 2001. Murine homolog of SALL1 is essential for ureteric bud invasion in kidney development. Development 128: 3105–3115.

Niu, Y., Dai, Z., Liu, W., Zhang, C., Yang, Y., Guo, Z., Li, X., Xu, C., Huang, X., Wang, Y. and Shi, Y.S., 2017. Ablation of SNX6 leads to defects in synaptic function of CA1 pyramidal neurons and spatial memory. Elife, 6, p.e20991.

Nolasco, S., Bellido, J., Gonçalves, J., Tavares, A., Zabala, J.C. and Soares, H., 2012. The expression of tubulin cofactor A (TBCA) is regulated by a noncoding antisense Tbca RNA during testis maturation.

Nolasco, S., Bellido, J., Serna, M., Carmona, B., Soares, H. and Zabala, J.C., 2021. Colchicine blocks tubulin heterodimer recycling by tubulin cofactors TBCA, TBCB, and TBCE. Frontiers in cell and developmental biology, 9, p.656273.

Nur-E-Kamal, A., Meiners, S., Ahmed, I., Azarova, A., Lin, C.P., Lyu, Y.L. and Liu, L.F., 2007. Role of DNA topoisomerase IIβ in neurite outgrowth. Brain research, 1154, pp.50-60.

Obba S., Hizir Z., Boyer L., Selimoglu-Buet D., Pfeifer A., Michel G., Hamouda M. A., Gonçalvès, D., Cerezo M., Marchetti S., Rocchi S., Droin N., Cluzeau T., Robert G., Luciano F., Robaye B., Foretz M., Viollet B., Legros L., Solary E., Auberger P., and Jacquel A., The PRKAA1/AMPKα1 pathway triggers autophagy during CSF1-induced human monocyte differentiation and is a potential target in CMML, Autophagy. (2015) 11, no. 7, 1114–1129

Ochocinska, M.J., Muñoz, E.M., Veleri, S., Weller, J.L., Coon, S.L., Pozdeyev, N., Michael Iuvone, P., Goebbels, S., Furukawa, T. and Klein, D.C., 2012. NeuroD1 is required for survival of photoreceptors but not pinealocytes: results from targeted gene deletion studies. Journal of neurochemistry, 123(1), pp.44-59.

O'Donnell SL, Frederick TJ, Krady JK, Vannucci SJ, Wood TL. IGF-I and microglia/macrophage proliferation in the ischemic mouse brain. Glia. 2002;39:85–97.

Ohta, S., Misawa, A., Fukaya, R., Inoue, S., Kanemura, Y., Okano, H., Kawakami, Y. and Toda, M., 2012. Macrophage migration inhibitory factor (MIF) promotes cell survival and proliferation of neural stem/progenitor cells. Journal of cell science, 125(13), pp.3210-3220.

Osborn AJ, Elledge SJ. Mrc1 is a replication fork component whose phosphorylation in response to DNA replication stress activates Rad53. Genes & Development. 2003;17:1755–1767.

Ott, L.E., Mcdowell, Z.T., Turner, P.M., Law, J.M., Adler, K.B., Yoder, J.A. and Jones, S.L., 2011. Two myristoylated alanine‐rich C‐kinase substrate (MARCKS) paralogs are required for normal development in zebrafish. The Anatomical Record: Advances in Integrative Anatomy and Evolutionary Biology, 294(9), pp.1511-1524.

Ow, Y.L.P., Green, D.R., Hao, Z. and Mak, T.W., 2008. Cytochrome c: functions beyond respiration. Nature reviews Molecular cell biology, 9(7), pp.532-542.

Owlanj, H., Yang, H.J. and Feng, Z.W., 2012. Nucleoside diphosphate kinase Nm23-M1 involves in oligodendroglial versus neuronal cell fate decision in vitro. Differentiation, 84(4), pp.281-293.

Parks, W.T., Frank, D.B., Huff, C., Haft, C.R., Martin, J., Meng, X., De Caestecker, M.P., McNally, J.G., Reddi, A., Taylor, S.I. and Roberts, A.B., 2001. Sorting nexin 6, a novel SNX, interacts with the transforming growth factor-β family of receptor serine-threonine kinases. Journal of Biological Chemistry, 276(22), pp.19332-19339.

Parsons, M.J., Brancaccio, M., Sethi, S., Maywood, E.S., Satija, R., Edwards, J.K., Jagannath, A., Couch, Y., Finelli, M.J., Smyllie, N.J. and Esapa, C., 2015. The regulatory factor ZFHX3 modifies circadian function in SCN via an AT motif-driven axis. Cell, 162(3), pp.607-621.

Patil, H., Cho, K.I., Lee, J., Yang, Y., Orry, A. and Ferreira, P.A., 2013. Kinesin-1 and mitochondrial motility control by discrimination of structurally equivalent but distinct subdomains in Ran-GTPbinding domains of Ran-binding protein 2. Open biology, 3(3), p.120183.

Paukovich, N., Xue, M., Elder, J.R., Redzic, J.S., Blue, A., Pike, H., Miller, B.G., Pitts, T.M., Pollock, D.D., Hansen, K. and D’Alessandro, A., 2018. Biliverdin reductase B dynamics are coupled to coenzyme binding. Journal of molecular biology, 430(18), pp.3234-3250.

Pedersen, M.Ø., Larsen, A., Stoltenberg, M. and Penkowa, M., 2009. The role of metallothionein in oncogenesis and cancer prognosis. Progress in histochemistry and cytochemistry, 44(1), pp.29-64.

Pellieux, C., Desgeorges, A., Pigeon, C.H., Chambaz, C., Yin, H., Hayoz, D. and Silacci, P., 2003. Cap G, a gelsolin family protein modulating protective effects of unidirectional shear stress. Journal of Biological Chemistry, 278(31), pp.29136-29144.

Perrone, L., Devi, T.S., Hosoya, K.I., Terasaki, T. and Singh, L.P., 2009. Thioredoxin interacting protein (TXNIP) induces inflammation through chromatin modification in retinal capillary endothelial cells under diabetic conditions. Journal of cellular physiology, 221(1), pp.262-272.

Pesaresi, M., Bonilla-Pons, S.A., Simonte, G., Sanges, D., Di Vicino, U. and Cosma, M.P., 2018. Endogenous mobilization of bone-marrow cells into the murine retina induces fusion-mediated reprogramming of Müller glia cells. EBioMedicine, 30, pp.38-51.

Petri, S., Grimmler, M., Over, S., Fischer, U. and Gruss, O.J., 2007. Dephosphorylation of survival motor neurons (SMN) by PPM1G/PP2Cγ governs Cajal body localization and stability of the SMN complex. The Journal of cell biology, 179(3), pp.451-465.

Polyak, S.L., 1941. The retina.

Poynton, R.A. and Hampton, M.B., 2014. Peroxiredoxins as biomarkers of oxidative stress. Biochimica et Biophysica Acta (BBA)-General Subjects, 1840(2), pp.906-912.

Pu, L., Meng, Q., Li, S., Wang, Y. and Liu, B., 2023. TXNRD1 knockdown inhibits the proliferation of endothelial cells subjected to oscillatory shear stress via activation of the endothelial nitric oxide synthase/apoptosis pathway. Biochimica et Biophysica Acta (BBA)-Molecular Cell Research, 1870(4), p.119436.

Puigdellívol, M., Allendorf, D.H. and Brown, G.C., 2020. Sialylation and galectin-3 in microgliamediated neuroinflammation and neurodegeneration. Frontiers in cellular neuroscience, 14, p.162.

Qin, N., Xu, D., Li, J. and Deng, X.W., 2020. COP9 signalosome: Discovery, conservation, activity, and function. Journal of Integrative Plant Biology, 62(1), pp.90-103.

Rahimian, R., Béland, L.C. and Kriz, J., 2018. Galectin-3: mediator of microglia responses in injured brain. Drug discovery today, 23(2), pp.375-381.

Rao, F., Lin, H. and Su, Y., 2020. Cullin-RING ligase regulation by the COP9 signalosome: structural mechanisms and new physiologic players. Cullin-RING Ligases and Protein Neddylation: Biology and Therapeutics, pp.47-60.

Rapaport, D., Auerbach, W., Naslavsky, N., Pasmanik‐Chor, M., Galperin, E., Fein, A., Caplan, S., Joyner, A.L. and Horowitz, M., 2006. Recycling to the plasma membrane is delayed in EHD1 knockout mice. Traffic, 7(1), pp.52-60.

Rayala, S.K., Martin, E., Sharina, I.G., Molli, P.R., Wang, X., Jacobson, R., Murad, F. and Kumar, R., 2007. Dynamic interplay between nitration and phosphorylation of tubulin cofactor B in the control of microtubule dynamics. Proceedings of the National Academy of Sciences, 104(49), pp.19470-19475.

Recio, C., Lucy, D., Purvis, G.S., Iveson, P., Zeboudj, L., Iqbal, A.J., Lin, D., O’Callaghan, C., Davison, L., Griesbach, E. and Russell, A.J., 2018. Activation of the immune-metabolic receptor GPR84 enhances inflammation and phagocytosis in macrophages. Frontiers in immunology, 9, p.1419.

Reeves, R., 2015. High mobility group (HMG) proteins: Modulators of chromatin structure and DNA repair in mammalian cells. DNA repair, 36, pp.122-136.

Rempel SA, Golembieski WA, Fisher JL, Maile M, Nakeff A. 2001. SPARC modulates cell growth, attachment and migration of U87 glioma cells on brain extracellular matrix proteins. J Neurooncol 53: 149–160.

Ren D, Tu HC, Kim H, Wang GX, Bean GR, Takeuchi O, Jeffers JR, Zambetti GP, Hsieh JJ, Cheng EH: BID, BIM, and PUMA are essential for activation of the BAX- and BAK-dependent cell death program. Science. 2010, 330: 1390-1393. 10.1126/science.1190217.

Ren, X., Zou, L., Zhang, X., Branco, V., Wang, J., Carvalho, C., Holmgren, A. and Lu, J., 2017. Redox signaling mediated by thioredoxin and glutathione systems in the central nervous system. Antioxidants & redox signaling, 27(13), pp.989-1010.

Rhee, S.G. and Woo, H.A., 2011. Multiple functions of peroxiredoxins: peroxidases, sensors and regulators of the intracellular messenger H2O2, and protein chaperones.

Rickman, C.B., Ebright, J.N., Zavodni, Z.J., Yu, L., Wang, T., Daiger, S.P., Wistow, G., Boon, K. and Hauser, M.A., 2006. Defining the human macula transcriptome and candidate retinal disease genes using EyeSAGE. Investigative ophthalmology & visual science, 47(6), pp.2305-2316.

Rios, H., Paganelli, A.R. and Fosser, N.S., 2020. The role of PDLIM1, a PDZ‐LIM domain protein, at the ribbon synapses in the chicken retina. Journal of Comparative Neurology, 528(11), pp.1820-1832.

Rocha-Martins, M., de Toledo, B.C., Santos-França, P.L., Oliveira-Valença, V.M., Vieira-Vieira, C.H., Matos-Rodrigues, G.E., Linden, R., Norden, C., Martins, R.A. and Silveira, M.S., 2019. De novo genesis of retinal ganglion cells by targeted expression of Klf4 in vivo. Development, 146(16), p.dev176586.

Rocha-Martins, M., Njaine, B. and Silveira, M.S., 2012. Avoiding pitfalls of internal controls: validation of reference genes for analysis by qRT-PCR and Western blot throughout rat retinal development.

Rochdi, M.D., Laroche, G., Dupré, E., Giguere, P., Lebel, A., Watier, V., Hamelin, E., Lépine, M.C., Dupuis, G. and Parent, J.L., 2004. Nm23-H2 interacts with a G protein-coupled receptor to regulate its endocytosis through an Rac1-dependent mechanism. Journal of Biological Chemistry, 279(18), pp.18981-18989.

Roger, J., Brajeul, V., Thomasseau, S., Hienola, A., Sahel, J.A., Guillonneau, X. and Goureau, O., 2006. Involvement of Pleiotrophin in CNTF-mediated differentiation of the late retinal progenitor cells. Developmental biology, 298(2), pp.527-539.

Roger, T., David, J., Glauser, M.P. and Calandra, T., 2001. MIF regulates innate immune responses through modulation of Toll-like receptor 4. Nature, 414(6866), pp.920-924.

Rotem, N., Magen, I., Ionescu, A., Gershoni-Emek, N., Altman, T., Costa, C.J., Gradus, T., PasmanikChor, M., Willis, D.E., Ben-Dov, I.Z. and Hornstein, E., 2017. ALS along the axons–expression of coding and noncoding RNA differs in axons of ALS models. Scientific Reports, 7(1), p.44500.

Ruiz-Perera, L.M., Arruti, C. and Zolessi, F.R., 2013. Early phosphorylation of MARCKS at Ser25 in migrating precursor cells and differentiating peripheral neurons. Neuroscience Letters, 544, pp.5-9.

Sáez, M.A., Fernández-Rodríguez, J., Moutinho, C., Sanchez-Mut, J.V., Gomez, A., Vidal, E., Petazzi, P., Szczesna, K., Lopez-Serra, P., Lucariello, M. and Lorden, P., 2016. Mutations in JMJD1C are involved in Rett syndrome and intellectual disability. Genetics in Medicine, 18(4), pp.378-385.

Salvatore, P., Contursi, C., Benvenuto, G., Bruni, C.B. and Chiariotti, L., 1995. Characterization and functional dissection of the galactin‐1 gene promoter. FEBS letters, 373(2), pp.159-163.

Sanchez-Vallejo, V., Benlloch-Navarro, S., Trachsel-Moncho, L., Lopez-Pedrajas, R., Almansa, I., Romero, F.J. and Miranda, M., 2016. Alterations in glutamate cysteine ligase content in the retina of two retinitis pigmentosa animal models. Free Radical Biology and Medicine, 96, pp.245-254.

Sangaletti S, Tripodo C, Cappetti B, Casalini P, Chiodoni C, Piconese S, Santangelo A, Parenza M, Arioli I, Miotti S, Colombo MP. SPARC oppositely regulates inflammation and fibrosis in bleomycin-induced lung damage. Am J Pathol. 2011;179:3000–3010.

Santos, J.M., Tewari, S., Goldberg, A.F. and Kowluru, R.A., 2011. Mitochondrial biogenesis and the development of diabetic retinopathy. Free Radical Biology and Medicine, 51(10), pp.1849-1860.

Saxena, G., Chen, J. and Shalev, A., 2010. Intracellular Shuttling and Mitochondrial Function of Thioredoxin-interacting Protein 2. Journal of Biological Chemistry, 285(6), pp.3997-4005.

Schaeren‐Wierners, N., André, E., Kapfhammer, J.P. and Becker‐André, M., 1997. The ExDression pattern of the orphan nuclear receptor RORβ in the developing and adult rat nervous system suggests a role in the processing of sensory information and in circadian rhythm. European Journal of Neuroscience, 9(12), pp.2687-2701.

Schafranek, M., Ruiz De Los Mozos, I., Wittig, I., McNicoll, F. and Schulz, M.H., 2020. SRSF7 maintains its homeostasis through the expression of Split-ORFs and nuclear body assembly. Nature structural & molecular biology, 27(3), pp.260-273.

Scheiblin, D.A., Gao, J., Caplan, J.L., Simirskii, V.N., Czymmek, K.J., Mathias, R.T. and Duncan, M.K., 2014. Beta-1 integrin is important for the structural maintenance and homeostasis of differentiating fiber cells. The international journal of biochemistry & cell biology, 50, pp.132-145.

Schlattner, U., 2021. The complex functions of the NME family—a matter of location and molecular activity. International journal of molecular sciences, 22(23), p.13083.

Schniepp, R., Kohler, K., Ladewig, T., Guenther, E., Henke, G., Palmada, M., Boehmer, C., Rothstein, J.D., Bröer, S. and Lang, F., 2004. Retinal colocalization and in vitro interaction of the glutamate receptor EAAT3 and the serum-and glucocorticoid-inducible kinase SGK1. Investigative ophthalmology & visual science, 45(5), pp.1442-1449.

Schöler, J., Ferralli, J., Thiry, S. and Chiquet-Ehrismann, R., 2015. The intracellular domain of teneurin1 induces the activity of microphthalmia-associated transcription factor (MITF) by binding to transcriptional repressor HINT1. Journal of Biological Chemistry, 290(13), pp.8154-8165.

Seipold, S., Priller, F.C., Goldsmith, P., Harris, W.A., Baier, H. and Abdelilah-Seyfried, S., 2009. NonSMC condensin I complex proteins control chromosome segregation and survival of proliferating cells in the zebrafish neural retina. BMC Developmental Biology, 9, pp.1-14.

Shalloe, F., Elliott, G., Ennis, O. and Mantle, T.J., 1996. Evidence that biliverdin-IX β reductase and flavin reductase are identical. Biochemical Journal, 316(2), pp.385-387.

Shi, Y., Sullivan, S.K., Pitterle, D.M., Kennington, E.A., Graff, J.M. and Blackshear, P.J., 1997. Mechanisms of MARCKS Gene Activation during XenopusDevelopment. Journal of Biological Chemistry, 272(46), pp.29290-29300.

Shkolnik-Yarros, E.G., 1971. Neurons of the cat's retina. Vision Research, 11(1), pp.7-IN9. Shuai, Y., Fan, E., Zhong, Q., Chen, Q., Feng, G., Gou, X. and Zhang, G., 2021. CDCA8 as an independent predictor for a poor prognosis in liver cancer. Cancer Cell International, 21, pp.1-10.

Shuai, Y., Fan, E., Zhong, Q., Chen, Q., Feng, G., Gou, X. and Zhang, G., 2021. CDCA8 as an independent predictor for a poor prognosis in liver cancer. Cancer Cell International, 21, pp.1-10.

Simonetti, B., Danson, C.M., Heesom, K.J. and Cullen, P.J., 2017. Sequence-dependent cargo recognition by SNX-BARs mediates retromer-independent transport of CI-MPR. Journal of Cell Biology, 216(11), pp.3695-3712.

Singh, H.D., Ma, J.X. and Takahashi, Y., 2021. Distinct roles of LRP5 and LRP6 in Wnt signaling regulation in the retina. Biochemical and biophysical research communications, 545, pp.8-13.

Singh, L.P., 2013. Thioredoxin interacting protein (TXNIP) and pathogenesis of diabetic retinopathy. Journal of clinical & experimental ophthalmology, 4.

Slišković, I., Eich, H. and Müller-McNicoll, M., 2022. Exploring the multifunctionality of SR proteins. Biochemical Society Transactions, 50(1), pp.187-198.

Smit, T., Ormel, P.R., Sluijs, J.A., Hulshof, L.A., Middeldorp, J., de Witte, L.D., Hol, E.M. and Donega, V., 2022. Transcriptomic and functional analysis of Aβ1-42 oligomer-stimulated human monocytederived microglia-like cells. Brain, behavior, and immunity, 100, pp.219-230.

Smyth, P., Sasiwachirangkul, J., Williams, R. and Scott, C.J., 2022. Cathepsin S (CTSS) activity in health and disease-A treasure trove of untapped clinical potential. Molecular aspects of medicine, 88, p.101106.

Sobeih D, Hussein KA, Said N, Motamed K, Al-Shabrawey M. Deletion of SPARC Enhances Retinal Vaso-Obliteration in Mouse Model of Oxygen-Induced Retinopathy. HSOA J Ophthalmol Clin Res. 2014 Dec;1(1):002. Epub 2014 Aug 22. PMID: 26636134; PMCID: PMC4665627.

Solberg, R., Arduini, A., Escobar, J., Sastre, J., Saugstad, O.D. and Vento, M., 2010. 34 Early Gene Regulation in Retina and Choroid After Ashpyxia and Resuscitation in Newborn Piglets. Pediatric Research, 68(1), pp.20-20.

Song, L., Mao, J., Zhang, J., Ibrahim, M.M., Li, L.H. and Tang, J.W., 2014. Annexin A7 and its binding protein galectin-3 influence mouse hepatocellular carcinoma cell line in vitro. Biomedicine & Pharmacotherapy, 68(3), pp.377-384.

Sordella, R.; Jiang, W.; Chen, G.C.; Curto, M.; Settleman, J. Modulation of Rho GTPase signaling regulates a switch between adipogenesis and myogenesis. Cell 2003, 113, 147–158.

Starkova, T., Polyanichko, A., Tomilin, A.N. and Chikhirzhina, E., 2023. Structure and functions of HMGB2 protein. International Journal of Molecular Sciences, 24(9), p.8334.

Starokadomskyy, P., Escala Perez-Reyes, A. and Burstein, E., 2021. Immune dysfunction in mendelian disorders of POLA1 deficiency. Journal of clinical immunology, 41(2), pp.285-293.

Stoffelns, B.M. and Richard, G., 2010. Is buckle surgery still the state of the art for retinal detachments due to retinal dialysis?. Journal of Pediatric Ophthalmology & Strabismus, 47(5), pp.281-287.

Stroo, E., Janssen, L., Sin, O., Hogewerf, W., Koster, M., Harkema, L., Youssef, S.A., Beschorner, N., Wolters, A.H., Bakker, B. and Becker, L., 2023. Deletion of SERF2 in mice delays embryonic development and alters amyloid deposit structure in the brain. Life science alliance, 6(7).

Suemori, S., Shimazawa, M., Kawase, K., Satoh, M., Nagase, H., Yamamoto, T. and Hara, H., 2006. Metallothionein, an endogenous antioxidant, protects against retinal neuron damage in mice. Investigative ophthalmology & visual science, 47(9), pp.3975-3982.

Suh, E.J., Kim, T.Y. and Kim, S.H., 2006. PP2Cγ-mediated S-phase accumulation induced by the proteasome-dependent degradation of p21WAF1/CIP1. FEBS letters, 580(26), pp.6100-6104.

Sun, C., Wang, G., Wrighton, K.H., Lin, H., Songyang, Z., Feng, X.H. and Lin, X., 2016. Regulation of p27Kip1 phosphorylation and G1 cell cycle progression by protein phosphatase PPM1G. American journal of cancer research, 6(10), p.2207.

Sun, L.Q., Lee, D.W., Zhang, Q., Xiao, W., Raabe, E.H., Meeker, A., Miao, D., Huso, D.L. and Arceci, R.J., 2004. Growth retardation and premature aging phenotypes in mice with disruption of the SNF2-like gene, PASG. Genes & development, 18(9), pp.1035-1046.

Sun, M., Ahmad, N., Zhang, R. and Graw, J., 2018. Crybb2 associates with Tmsb4X and is crucial for dendrite morphogenesis. Biochemical and biophysical research communications, 503(1), pp.123130.

Sun, Yin, Xuedong Liu, Elinor Ng-Eaton, Harvey F. Lodish, and Robert A. Weinberg. "SnoN and Ski protooncoproteins are rapidly degraded in response to transforming growth factor β signaling." Proceedings of the National Academy of Sciences 96, no. 22 (1999): 12442-12447.

Suzuki, M., Takaishi, S., Nagasaki, M., Onozawa, Y., Iino, I., Maeda, H., Komai, T. and Oda, T., 2013. Medium-chain fatty acid-sensing receptor, GPR84, is a proinflammatory receptor. Journal of Biological Chemistry, 288(15), pp.10684-10691.

Swaroop, S., Sengupta, N., Suryawanshi, A.R., Adlakha, Y.K. and Basu, A., 2016. HSP60 plays a regulatory role in IL-1β-induced microglial inflammation via TLR4-p38 MAPK axis. Journal of neuroinflammation, 13, pp.1-19.

Syc-Mazurek, S.B., Fernandes, K.A., Wilson, M.P., Shrager, P. and Libby, R.T., 2017. Together JUN and DDIT3 (CHOP) control retinal ganglion cell death after axonal injury. Molecular neurodegeneration, 12, pp.1-13.

Takenouchi, T., Tsukimoto, M., Iwamaru, Y., Sugama, S., Sekiyama, K., Sato, M., Kojima, S., Hashimoto, M. and Kitani, H., 2015. Extracellular ATP induces unconventional release of glyceraldehyde-3phosphate dehydrogenase from microglial cells. Immunology letters, 167(2), pp.116-124.

Tang, C., Jiang, Y., Shao, W., Shi, W., Gao, X., Qin, W., Jiang, T., Wang, F. and Feng, S., 2016. Abnormal expression of FOSB correlates with tumor progression and poor survival in patients with gastric cancer. International journal of oncology, 49(4), pp.1489-1496.

Taylor, R.A., Chang, C.F., Goods, B.A., Hammond, M.D., Mac Grory, B., Ai, Y., Steinschneider, A.F., Renfroe, S.C., Askenase, M.H., McCullough, L.D. and Kasner, S.E., 2017. TGF-β1 modulates microglial phenotype and promotes recovery after intracerebral hemorrhage. The Journal of clinical investigation, 127(1), pp.280-292.

Taylor, V. and Suter, U., 1996. Epithelial membrane protein-2 and epithelial membrane protein-3: two novel members of the peripheral myelin protein 22 gene family. Gene, 175(1-2), pp.115-120.

Tecalco-Cruz, A.C., Sosa-Garrocho, M., Vázquez-Victorio, G., Ortiz-García, L., Domínguez-Hüttinger, E. and Macías-Silva, M., 2012. Transforming growth factor-β/SMAD Target gene SKIL is negatively regulated by the transcriptional cofactor complex SNON-SMAD4. Journal of Biological Chemistry, 287(32), pp.26764-26776.

Tian, G., Huang, Y., Rommelaere, H., Vandekerckhove, J., Ampe, C. and Cowan, N.J., 1996. Pathway leading to correctly folded β-tubulin. Cell, 86(2), pp.287-296.

Tsutsui, K., Okada, S., Watanabe, M., Shohmori, T., Seki, S. and Inoue, Y., 1993. Molecular cloning of partial cDNAs for rat DNA topoisomerase II isoforms and their differential expression in brain development. Journal of Biological Chemistry, 268(25), pp.19076-19083.

Turk, D., Janjić, V., Štern, I., Podobnik, M., Lamba, D., Dahl, S.W., Lauritzen, C., Pedersen, J., Turk, V. and Turk, B., 2001. Structure of human dipeptidyl peptidase I (cathepsin C): exclusion domain added to an endopeptidase framework creates the machine for activation of granular serine proteases. The EMBO journal.

Turk, V., Stoka, V., Vasiljeva, O., Renko, M., Sun, T., Turk, B. and Turk, D., 2012. Cysteine cathepsins: from structure, function and regulation to new frontiers. Biochimica et Biophysica Acta (BBA)-Proteins and Proteomics, 1824(1), pp.68-88.

Vadlamudi, R.K., Barnes, C.J., Rayala, S., Li, F., Balasenthil, S., Marcus, S., Goodson, H.V., Sahin, A.A. and Kumar, R., 2005. p21-activated kinase 1 regulates microtubule dynamics by phosphorylating tubulin cofactor B. Molecular and cellular biology, 25(9), pp.3726-3736.

Van Weering, J.R., Sessions, R.B., Traer, C.J., Kloer, D.P., Bhatia, V.K., Stamou, D., Carlsson, S.R., Hurley, J.H. and Cullen, P.J., 2012. Molecular basis for SNX‐BAR‐mediated assembly of distinct endosomal sorting tubules. The EMBO journal, 31(23), pp.4466-4480.

Varga, J., Korbai, S., Neller, A., Zsindely, N. and Bodai, L., 2019. Hat1 acetylates histone H4 and modulates the transcriptional program in Drosophila embryogenesis. Scientific reports, 9(1), p.17973.

Vasanthakumar, T. and Rubinstein, J.L., 2020. Structure and roles of V-type ATPases. Trends in biochemical sciences, 45(4), pp.295-307.

Vincent, A.J.; Lau, P.W.; Roskams, A.J. SPARC is expressed by macroglia and microglia in the developing and mature nervous system. Dev. Dyn. 2008, 237, 1449–1462.

Voigt, Andrew P., S. Scott Whitmore, Kelly Mulfaul, Kathleen R. Chirco, Joseph C. Giacalone, Miles J. Flamme-Wiese, Adam Stockman et al. "Bulk and single-cell gene expression analyses reveal aging human choriocapillaris has pro-inflammatory phenotype." Microvascular research 131 (2020): 104031.

Von Lindern, M., Fornerod, M., Van Baal, S., Jaegle, M., De Wit, T., Buijs, A. and Grosveld, G., 1992. The translocation (6; 9), associated with a specific subtype of acute myeloid leukemia, results in the fusion of two genes, dek and can, and the expression of a chimeric, leukemia-specific dek-can mRNA. Molecular and cellular biology, 12(4), pp.1687-1697.

Waksmunski, A.R., Grunin, M., Kinzy, T.G., Igo, R.P., Haines, J.L., Bailey, J.N.C. and International Age-Related Macular Degeneration Genomics Consortium, 2019. Pathway analysis integrating genomewide and functional data identifies PLCG2 as a candidate gene for age-related macular degeneration. Investigative ophthalmology & visual science, 60(12), pp.4041-4051.

Walseng, E., Bakke, O. and Roche, P.A., 2008. Major histocompatibility complex class II-peptide complexes internalize using a clathrin-and dynamin-independent endocytosis pathway. Journal of Biological Chemistry, 283(21), pp.14717-14727.

Wang, B., Wang, L., Gu, S., Yu, Y., Huang, H., Mo, K., Xu, H., Zeng, F., Xiao, Y., Peng, L. and Liu, C., 2020. D609 protects retinal pigmented epithelium as a potential therapy for age-related macular degeneration. Signal transduction and targeted therapy, 5(1), p.20.

Wang, D., Tang, L., Shi, W., Mei, H., Yang, R., Yang, Y., Hu, Y., Fang, J. and Guo, T., 2011. A Novel Nonsense Mutation of F13A1 Gene Causing Inherited Factor XIII Deficiency in a Chinese Han Family. Blood, 118(21), p.4661.

Wang, J., Liu, X., Wang, H., Qin, L., Feng, A., Qi, D., Wang, H., Zhao, Y., Kong, L., Wang, H. and Wang, L., 2022. JMJD1C Regulates Megakaryopoiesis in In Vitro Models through the Actin Network. Cells, 11(22), p.3660.

Wang, L., Ebrahimi, K.B., Chyn, M., Cano, M. and Handa, J.T., 2016. Biology of p62/sequestosome-1 in age-related macular degeneration (AMD). Retinal Degenerative Diseases: Mechanisms and Experimental Therapy, pp.17-22.

Wang, Q. and Shen, W.H., 2018. Chromatin modulation and gene regulation in plants: insight about PRC1 function. Biochemical Society Transactions, 46(4), pp.957-966.

Wang, S.K., Xue, Y. and Cepko, C.L., 2020. Microglia modulation by TGF-β1 protects cones in mouse models of retinal degeneration. The Journal of clinical investigation, 130(8), pp.4360-4369.

Wang, Y.W., Cheng, H.L., Ding, Y.R., Chou, L.H. and Chow, N.H., 2017. EMP1, EMP 2, and EMP3 as novel therapeutic targets in human cancer. Biochimica et Biophysica Acta (BBA)-Reviews on Cancer, 1868(1), pp.199-211.

Wang, Z., Fan, M., Candas, D., Zhang, T.Q., Qin, L., Eldridge, A., Wachsmann-Hogiu, S., Ahmed, K.M., Chromy, B.A., Nantajit, D. and Duru, N., 2014. Cyclin B1/Cdk1 coordinates mitochondrial respiration for cell-cycle G2/M progression. Developmental cell, 29(2), pp.217-232.

Waters, A.M., Asfahani, R., Carroll, P., Bicknell, L., Lescai, F., Bright, A., Chanudet, E., Brooks, A., Christou-Savina, S., Osman, G. and Walsh, P., 2015. The kinetochore protein, CENPF, is mutated in human ciliopathy and microcephaly phenotypes. Journal of medical genetics, 52(3), pp.147-156.

Wei, Z., Gao, F., Kim, S., Yang, H., Lyu, J., An, W., Wang, K. and Lu, W., 2013. Klf4 organizes long-range chromosomal interactions with the oct4 locus in reprogramming and pluripotency. Cell stem cell, 13(1), pp.36-47.

Williams, C.C., Jan, C.H. and Weissman, J.S., 2014. Targeting and plasticity of mitochondrial proteins revealed by proximity-specific ribosome profiling. Science, 346(6210), pp.748-751.

Wrana, J.L., Attisano, L., Wieser, R., Ventura, F. and Massagué, J., 1994. Mechanism of activation of the TGF-β receptor. Nature, 370(6488), pp.341-347.

Wright MD, Tomlinson MG. The ins and outs of the transmembrane 4 superfamily. Immunol Today. 1994;15:588–594.

Wu, S., Li, Z., Gnatenko, D.V., Zhang, B., Zhao, L., Malone, L.E., Markova, N., Mantle, T.J., Nesbitt, N.M. and Bahou, W.F., 2016. BLVRB redox mutation defines heme degradation in a metabolic pathway of enhanced thrombopoiesis in humans. Blood, The Journal of the American Society of Hematology, 128(5), pp.699-709.

Wunderlich, K.A., Tanimoto, N., Grosche, A., Zrenner, E., Pekny, M., Reichenbach, A., Seeliger, M.W., Pannicke, T. and Perez, M.T., 2015. Retinal functional alterations in mice lacking intermediate filament proteins glial fibrillary acidic protein and vimentin. The FASEB Journal, 29(12), pp.4815-4828.

Xia, X., Yu, C.Y., Bian, M., Sun, C.B., Tanasa, B., Chang, K.C., Bruffett, D.M., Thakur, H., Shah, S.H., Knasel, C. and Cameron, E.G., 2020. MEF2 transcription factors differentially contribute to retinal ganglion cell loss after optic nerve injury. PloS one, 15(12), p.e0242884.

Xu, Z., Yu, Y. and Duh, E.J., 2006. Vascular endothelial growth factor upregulates expression of ADAMTS1 in endothelial cells through protein kinase C signaling. Investigative ophthalmology & visual science, 47(9), pp.4059-4066.

Yaffe, M.B., Farr, G.W., Miklos, D., Horwich, A.L., Sternlicht, M.L. and Sternlicht, H., 1992. TCP1 complex is a molecular chaperone in tubulin biogenesis. Nature, 358(6383), pp.245-248.

Yamamoto, T., Kanda, A., Kase, S. and Ishida, S., 2021. Hypoxia induces galectin-1 expression via autoinduction of placental growth factor in retinal pigment epithelium cells. Investigative ophthalmology & visual science, 62(2), pp.22-22.

Yamamoto, T., Kanda, A., Kase, S. and Ishida, S., 2021. Hypoxia induces galectin-1 expression via autoinduction of placental growth factor in retinal pigment epithelium cells. Investigative ophthalmology & visual science, 62(2), pp.22-22.

Yang, F., Li, W.B., Qu, Y.W., Gao, J.X., Tang, Y.S., Wang, D.J. and Pan, Y.J., 2020. Bone marrow mesenchymal stem cells induce M2 microglia polarization through PDGF-AA/MANF signaling. World journal of stem cells, 12(7), p.633.

Yang, H., S. Zheng, Y. Mao, Z. Chen, C. Zheng, H. Li, C. Sumners, Q. Li, P. Yang, and B. Lei. "Modulating of ocular inflammation with macrophage migration inhibitory factor is associated with notch signalling in experimental autoimmune uveitis." Clinical & Experimental Immunology 183, no. 2 (2016): 280-293.

Yang, L.P., Wu, L.M., Guo, X.J. and Tso, M.O., 2007. Activation of endoplasmic reticulum stress in degenerating photoreceptors of the rd1 mouse. Investigative ophthalmology & visual science, 48(11), pp.5191-5198.

Yang, M., Hussain, H.M.J., Khan, M., Muhammad, Z., Zhou, J., Ma, A., Huang, X., Ye, J., Chen, M., Zhi, A. and Liu, T., 2025. Deficiency in DNAH12 causes male infertility by impairing DNAH1 and DNALI1 recruitment in humans and mice. eLife, 13, p.RP100350.

Yang, Y.I., Dieter, M.Z., Chen, Y., Shertzer, H.G., Nebert, D.W. and Dalton, T.P., 2002. Initial characterization of the glutamate-cysteine ligase modifier subunit Gclm (−/−) knockout mouse: novel model system for a severely compromised oxidative stress response. Journal of Biological Chemistry, 277(51), pp.49446-49452.

Yeeles JT, Deegan TD, Janska A, Early A, Diffley JF. Regulated eukaryotic DNA replication origin firing with purified proteins. Nature. 2015;519:431–435.

Yi, W., Lu, Y., Zhong, S., Zhang, M., Sun, L., Dong, H., Wang, M., Wei, M., Xie, H., Qu, H. and Peng, R., 2021. A single-cell transcriptome atlas of the aging human and macaque retina. National Science Review, 8(4), p.nwaa179.

Yoon, T., Kim, M. and Lee, K., 2006. Inhibition of Na, K-ATPase-suppressive activity of translationally controlled tumor protein by sorting nexin 6. FEBS letters, 580(14), pp.3558-3564.

Yoshihara, E., Masaki, S., Matsuo, Y., Chen, Z., Tian, H. and Yodoi, J., 2014. Thioredoxin/Txnip: redoxisome, as a redox switch for the pathogenesis of diseases. Frontiers in immunology, 4, p.514.

Yu M., Chen Y., Zeng H., Zheng Y., Fu G., Zhu W., Broeckel U., Aggarwal P., Turner A., Neale G., Guy C., Zhu N., Chi H., Wen R., and Wang D., PLCγ-dependent mTOR signalling controls IL-7-mediated early B cell development, Nature Communications. (2017) 8, no. 1

Yu, D., Makkar, G., Dong, T., Strickland, D.K., Sarkar, R. and Monahan, T.S., 2015. MARCKS signaling differentially regulates vascular smooth muscle and endothelial cell proliferation through a KIS-, p27kip1-dependent mechanism. PLoS One, 10(11), p.e0141397.

Yu, J.T., Liu, Y., Dong, P., Cheng, R.E., Ke, S.X., Chen, K.Q., Wang, J.J., Shen, Z.S., Tang, Q.Y. and Zhang, Z., 2019. Up-regulation of antioxidative proteins TRX1, TXNL1 and TXNRD1 in the cortex of PTZ kindling seizure model mice. PloS one, 14(1), p.e0210670.

Yuksel, S., Aredo, B., Zegeye, Y., Zhao, C.X., Tang, M., Li, X., Hulleman, J.D., Gautron, L., Ludwig, S., Moresco, E.M. and Butovich, I.A., 2023. Forward genetic screening using fundus spot scale identifies an essential role for Lipe in murine retinal homeostasis. Communications biology, 6(1), p.533.

Zhang JL, Chen GW, Liu YC, Wang PY, Wang X, et al. Secreted Protein Acidic and Rich in Cysteine (SPARC) suppresses angiogenesis by down-regulating the expression of VEGF and MMP-7 in gastric cancer. PLoS One. 2012;7:44618.

Zhang, E.D., Li, C., Fang, Y., Li, N., Xiao, Z., Chen, C., Wei, B., Wang, H., Xie, J., Miao, Y. and Zeng, Z., 2022. STMN1 as a novel prognostic biomarker in HCC correlating with immune infiltrates and methylation. World Journal of Surgical Oncology, 20(1), p.301.

Zhang, J., Zhang, T., Zeng, S., Zhang, X., Zhou, F., Gillies, M.C. and Zhu, L., 2023. The Role of Nrf2/sMAF Signalling in Retina Ageing and Retinal Diseases. Biomedicines, 11(6), p.1512.

Zhang, N., Zhu, H.P., Huang, W., Wen, X., Xie, X., Jiang, X., Peng, C., Han, B. and He, G., 2022. Unraveling the structures, functions and mechanisms of epithelial membrane protein family in human cancers. Experimental hematology & oncology, 11(1), p.69.

Zhang, Q. and Guo, Z., 2022. SEC61G participates in endoplasmic reticulum stress by interacting with CREB3 to promote the malignant progression of lung adenocarcinoma. Oncology Letters, 24(1), pp.1-9.

Zhang, S., Liu, X., Bawa-Khalfe, T., Lu, L.S., Lyu, Y.L., Liu, L.F. and Yeh, E.T., 2012. Identification of the molecular basis of doxorubicin-induced cardiotoxicity. Nature medicine, 18(11), pp.1639-1642.

Zhang, S., Yang, J., Ji, D., Meng, X., Zhu, C., Zheng, G., Glessner, J., Qu, H.Q., Cui, Y., Liu, Y. and Wang, W., 2024. NASP gene contributes to autism by epigenetic dysregulation of neural and immune pathways. Journal of Medical Genetics, 61(7), pp.677-688.

Zhang, S.X., Unruh, B., Arthur, K., Wang, J., Kim, I.K., Owen, L., Farkas, M.H. and DeAngelis, M.M., 2023. Expression and Regulation of Humanin Nuclear Isoform Genes in the RPE and Age-related Macular Degeneration. Investigative Ophthalmology & Visual Science, 64(8), pp.2740-2740.

Zhang, Y., Zhao, Z., Zhao, X., Xie, H., Zhang, C., Sun, X. and Zhang, J., 2022. HMGB2 causes photoreceptor death via down-regulating Nrf2/HO-1 and up-regulating NF-κB/NLRP3 signaling pathways in light-induced retinal degeneration model. Free Radical Biology and Medicine, 181, pp.14-28.

Zhang, Zhen-Zhen, Xiu-Hong Qin, and Jing Zhang. "MicroRNA-183 inhibition exerts suppressive effects on diabetic retinopathy by inactivating BTG1-mediated PI3K/Akt/VEGF signaling pathway." American Journal of Physiology-Endocrinology and Metabolism 316, no. 6 (2019): E1050-E1060.

Zhao, M., Tao, Y. and Peng, G.H., 2020. The role of histone acetyltransferases and histone deacetylases in photoreceptor differentiation and degeneration. International Journal of Medical Sciences, 17(10), p.1307.

Zhong, Q., Mishra, M. and Kowluru, R.A., 2013. Transcription factor Nrf2-mediated antioxidant defense system in the development of diabetic retinopathy. Investigative ophthalmology & visual science, 54(6), pp.3941-3948.

Zhou, J. and Ekström, P., 2022. A potential role of cyclic dependent kinase 1 (CDK1) in late stage of retinal degeneration. Cells, 11(14), p.2143.

Zhou, X., Uddin, M., Djukanovic, R. and Walls, A.F., 2009. Dipeptidyl Peptidase I (DPPI) as a Modulator of Elastase Activity in Human Blood Neutrophils. Journal of Allergy and Clinical Immunology, 123(2), p.S59.

Zhu, Z., Zhang, W., Huo, S., Huang, T., Cao, X. and Zhang, Y., 2024. TUBB, a robust biomarker with satisfying abilities in diagnosis, prognosis, and immune regulation via a comprehensive pan-cancer analysis. Frontiers in Molecular Biosciences, 11, p.1365655.

Ziaka, K. and van der Spuy, J., 2022. The role of Hsp90 in retinal proteostasis and disease. Biomolecules, 12(7), p.978.

Zimoń, M., Baets, J., Almeida-Souza, L., De Vriendt, E., Nikodinovic, J., Parman, Y., Battaloǧlu, E., Matur, Z., Guergueltcheva, V., Tournev, I. and Auer-Grumbach, M., 2012. Loss-of-function mutations in HINT1 cause axonal neuropathy with neuromyotonia. Nature genetics, 44(10), pp.1080-1083.

Zocchi, L., Mehta, A., Wu, S.C., Wu, J., Gu, Y., Wang, J., Suh, S., Spitale, R.C. and Benavente, C.A., 2020. Chromatin remodeling protein HELLS is critical for retinoblastoma tumor initiation and progression. Oncogenesis, 9(2), p.25.

Zolessi, F.R. and Arruti, C., 2001. Sustained phosphorylation of MARCKS in differentiating neurogenic regions during chick embryo development. Developmental Brain Research, 130(2), pp.257-267.
