# Supplementary material for: Developmental synchrony of retinal waves, apoptosis, and angiogenesis in postnatal retina
Source: eLife. 2026 Sep 24;15:RP111419. doi: 10.7554/eLife.111419 (PMC13609470; doi:10.7554/eLife.111419)
Supplement: Peer review history

# Editor report: eLife Assessment

**DOI:** 10.7554/eLife.111419.3.sa0

**Authors:** Xiaorong Liu

**Affiliations:** University of Virginia United States

**Keywords:** Convincing, Important

This study identifies apoptotic retinal ganglion cells as a potential source of ATP-mediated activation of PANX1 channels that initiates developmental retinal Ca²⁺ waves and coordinates microglial activation and vascular outgrowth during postnatal maturation. The work is **important** because it proposes an integrative framework linking programmed cell death, spontaneous neural activity, immune responses, and angiogenesis into a self-regulating developmental loop. Although the mechanistic conclusions would benefit from complementary genetic validation, the study provides a **convincing** foundation for future investigations into the coordination of neural circuit development and tissue remodeling.

# Referee report: Reviewer #1 (Public review)

**DOI:** 10.7554/eLife.111419.3.sa1

Summary:

This study presents a potentially important integrative model linking spontaneous retinal waves, apoptosis, microglial activity, and vascular development during postnatal retinal maturation. Its significance lies in proposing a mechanistic framework that could reshape understanding of how neural activity and tissue remodeling are coordinated in the developing central nervous system. The evidence is strengthened by the use of multiple complementary techniques, including Ca++ imaging, high-throughput electrophysiology, transcriptomics, histology and pharmacology.

Strengths:

(1) Multimodal Validation: The authors correlate large-scale functional imaging (calcium imaging and MEA) with high-resolution structural and molecular data (scRNA-seq and IHC), providing strong topographical evidence for the "centrifugal expansion" pattern.

(2) The primary significance lies in identifying apoptotic Retinal Ganglion Cells (RGCs) as the physiological "pacemakers" for stage II retinal waves. By linking programmed cell death directly to neural activity and subsequent angiogenesis, the authors propose a self-regulating developmental loop.

Weaknesses:

(1) While the PANX1 pharmacological data provides compelling functional support, extending these conclusions to the broader CNS may be premature. Additional direct mechanistic validation would further strengthen the claim of causality.

(2) While the manuscript beautifully illustrates the co-occurrence of events during retinal development, strengthening the distinction between correlation and direct causation would enhance the impact of the findings.

Appraisal of Aims and Conclusions:

The authors successfully achieve their aim of presenting a cohesive, multi-layered framework for postnatal retinal maturation, aligning functional physiological data with structural and transcriptomic timelines. The data robustly supports the correlation between retinal waves, microglial activity, and vascular remodeling and also identifies apoptotic RGCs as the potential "pacemakers" of Stage II waves.

Impact, Utility, and Community Asset:

This work will significantly impact developmental neurobiology by reframing programmed cell death as an active, instructive driver of neural network patterning and angiogenesis, rather than a passive clearance process. Methodologically, the integration of large-scale MEA recordings and live calcium imaging with scRNA-seq sets an excellent benchmark for multimodal developmental studies. Furthermore, the transcriptomic datasets mapping microglial phenotypes and vascular remodeling will serve as a highly valuable reference repository for the broader visual neuroscience community.

Additional Context for Readers:

To fully appreciate this study, readers should view it through the lens of neurovascular unit assembly. While Stage II cholinergic waves are traditionally studied purely in the context of visual circuit refinement, this work adds vital context by showing they also regulate the surrounding metabolic ecosystem. It effectively demonstrates that early electrical activity, programmed cell death, and vascular scaffolding do not occur in isolation, but are deeply interdependent processes.

# Referee report: Reviewer #2 (Public review)

**DOI:** 10.7554/eLife.111419.3.sa2

Summary:

Savage et al. investigates the synchronization of retinal Ca2+ waves with developmental cell death, microglia activation, and vascular outgrowth. These developmental processes occur through a mechanism where apoptotic cells release ATP through Panx-1 channels to stimulate both Ca2+ retinal waves and microglia activation. Using scRNAseq, the authors classify autofluorescence cell clusters (ACCs) at the leading edge of vasculature outgrowth as Hmox-1+ microglia. From here they show microglia engulfment of apoptotic RGCs and the potential release of ATP may contribute to Ca2+ wave generation. The authors demonstrate these mechanisms through the use of two pharmacological to agents to either block the ATP release from Panx-1 or by blocking receptor binding to ATP. Furthermore, while previous studies have described the site of initiation of retinal Ca2+ waves as random, this study shows the initiation of Ca2+ waves are biased to the leading edge of vascular growth in the developing retina. To do this, the authors use a combination of wide-field Ca2+ imaging and multi-electrode arrays to pinpoint the sites of Ca2+ wave initiation in the developing retina.

Strengths:

Savage et al. uses a several techniques to interrogate these mechanisms, including single cell RNAseq, wide-field Ca2+ imaging, and multi-electrode arrays. With these experiments, this manuscript proposes several novel ideas, such as ATP as the Ca2+ wave initiating cue, and the localization the Ca2+ wave initiation to the leading edge of vascular growth.

Weaknesses:

The main limitation of this study is the reliance on only two pharmacological agents to test their central hypotheses. In future studies, these conclusions could be strengthened if they used genetic knockout models to perturb programmed cell death and/or ATP release (i.e. BAX-KO, Panx-1 KO).

# Author comment: Author response

**DOI:** 10.7554/eLife.111419.3.sa3

**Authors:** Michael Savage, Cori Bertram, Jean de Montigny, Courtney A Thorne, Rachel Queen, Majlinda Lako, Gerrit Hilgen, Evelyne Sernagor

**Affiliations:** Newcastle University Newcastle upon Tyne United Kingdom; Newcastle University Newcastle upon Tyne United Kingdom; Inserm Montpellier France; University of Auckland Auckland New Zealand; Newcastle University Newcastle upon Tyne United Kingdom; Newcastle University Newcastle upon Tyne United Kingdom; Newcastle University Newcastle upon Tyne United Kingdom; Newcastle University Newcastle upon Tyne United Kingdom

The following is the authors’ response to the original reviews.

**Public Reviews:**

**Reviewer #1 (Public review):**

Summary:

This study presents a potentially important integrative model linking spontaneous retinal waves, apoptosis, microglial activity, and vascular development during postnatal retinal maturation. Its significance lies in proposing a mechanistic framework that could reshape understanding of how neural activity and tissue remodeling are coordinated in the developing central nervous system. The evidence is strengthened by the use of multiple complementary techniques, including Ca++ imaging, high-throughput electrophysiology, transcriptomics, histology, and pharmacology.

Strengths:

(1) Multimodal Validation: The authors correlate large-scale functional imaging (calcium imaging and MEA) with high-resolution structural and molecular data (scRNA-seq and IHC), providing strong topographical evidence for the "centrifugal expansion" pattern.

(2) The primary significance lies in identifying apoptotic Retinal Ganglion Cells (RGCs) as the physiological "pacemakers" for stage II retinal waves. By linking programmed cell death directly to neural activity and subsequent angiogenesis, the authors propose a self-regulating developmental loop.

We thank the reviewer for their nice summary and for highlighting the strengths of this work.

Weaknesses:

(1) While the PANX1 pharmacological data provide compelling functional support, extending these conclusions to the broader CNS may be premature. Additional direct mechanistic validation would further strengthen the claim of causality.

We agree with the reviewer that the conclusions would be greatly solidified with more direct mechanistic validation. However, we are unable to conduct more experimentation as the grant is finished and the Sernagor lab is in the process of being shutdown, after the unexpected passing of the PI.

In order to make clearer that this mechanism was found in retinal tissue, not CNS, we have moved any mention of the implications of our work to a broader CNS mechanism to the discussion section. We have also added text into the discussion highlighting the need for more mechanistic investigation to uncover the full extent of the developmental processes described herein, see Line 413.

(2) While the manuscript beautifully illustrates the co-occurrence of events during retinal development, strengthening the distinction between correlation and direct causation would enhance the impact of the findings.

We have been clear to only present our findings as correlational as we were unable to fully explore the causational nature within the mechanisms presented. In the discussion, we have used published evidence and experimental papers to bolster our understanding of the causal aspects of this research. We have also included sections of text to address what experimentation is be required to examine the causal interactions more directly, see Line 413.

**Reviewer #2 (Public review):**

Summary:

Savage et al. investigate the synchronization of retinal Ca2+ waves with developmental cell death, microglia activation, and vascular outgrowth. These developmental processes occur through a mechanism where apoptotic cells release ATP through Panx-1 channels to stimulate both Ca2+ retinal waves and microglia activation. Using scRNAseq, the authors classify autofluorescence cell clusters (ACCs) at the leading edge of vasculature outgrowth as Hmox-1+ microglia. From here, they show microglia engulfment of apoptotic RGCs, and the potential release of ATP may contribute to Ca2+ wave generation. The authors demonstrate these mechanisms through the use of two pharmacological agents to either block the ATP release from Panx-1 or block receptor binding to ATP. Furthermore, while previous studies have described the site of initiation of retinal Ca2+ waves as random, this study shows that the initiation of Ca2+ waves is biased to the leading edge of vascular growth in the developing retina. To do this, the authors use a combination of wide-field Ca2+ imaging and multi-electrode arrays to pinpoint the sites of Ca2+ wave initiation in the developing retina.

Strengths:

The authors use several techniques to interrogate these mechanisms, including single-cell RNAseq, wide-field Ca2+ imaging, and multi-electrode arrays. With these experiments, this manuscript proposes several novel ideas, such as ATP as the Ca2+ wave-initiating cue, and the localization of the Ca2+ wave initiation to the leading edge of vascular growth.

We thank the reviewer for their nice summary and for highlighting the strengths of this work.

Weaknesses:

The main weakness of the manuscript is the overreliance on only two pharmacological agents to test the central hypotheses. These conclusions would be strengthened if, in addition to their pharmacological manipulations, they used genetic knockout models to perturb programmed cell death or ATP release (i.e., BAX-KO, Panx-1 KO).

We thank the reviewer for their insightful suggestions for further experimentation to bolster the research. Initially, we utilised pharmacological interventions as they provided acute and quick answering of the research question. At the outset of the research, we were not certain that purinergic release through PANX-1 channels was the mediator for the developmental mechanisms described. We tested a wide variety of specific agonists and blockers before seeing any profound effects on wave generation. These agonists and antagonists have been used before and are proven to deliver reliable results. In addition, since the ACCs had never been reported before we were unsure if a knockout animal would display the same anatomical phenotype. Furthermore, it is known that knockout mouse lines, especially connexin and hemichannel pores, do not lose function but rather have other isoforms or compensation mechanisms which can substitute the original function. For the retina, for example, it was shown that Cx36 can functionally replace Cx45 after Cx45 KO (Frank et al, 2010).

We agree that while direct mechanistic validation would significantly reinforce the arguments, we are limited in conducting further experiments since the grant has been completed and the Sernagor lab is in the process of shutting down following her passing.

In order to address the omission of mechanistic validation in the paper we have added text into the discussion highlighting the need deeper investigation in the causality of the developmental processes described herein, see Line 413.

M. Frank et al., Neuronal connexin-36 can functionally replace connexin-45 in mouse retina but not in the developing heart, J. Cell Sci. 123, 3605 (2010).

**Recommendations for the authors:**

**Reviewer #1 (Recommendations for the authors):**

General and major comments

(A) Introduction

(1) The introduction is currently quite extensive. I recommend streamlining the background information to more directly frame the study's core objectives.

We have reduced the background information contained in the introduction to better align with the direct outputs of the study. However, as this research paper examines the interactions of multiple complex developmental processes a relatively in-depth introduction is needed to inform the reader of the salient points.

(2) To improve clarity, it would be highly beneficial to conclude the introduction with a sequential summary of key observations in the order they are presented in the study. This would provide a clearer roadmap for the reader.

We have reworked the end of the introduction to better align with the key observations in the order they are presented through the figures.

(B) Results

(3) The characterization of ACCs (Apoptotic Cell Clusters) would be more effective if separated from the description of their spatiotemporal occurrence with SVPs. Consider moving the microscopic description to a dedicated section or merging it with the subsequent paragraph on molecular identification.

We thank the reviewer for their suggestion to reorganise the results section. However, we believe that highlighting the integrated nature of the ACC positioning and development of the vascular plexus is an important stepping off point for the reader. It allows us to highlight the original serendipitous discovery of the ACCs and their highly cohesive role in bridging multiple developmental processes.

(4) Please explicitly state the specific retinal developmental stages (e.g., P3-P6) within the section regarding RNA sequencing, as this timing is critical for interpreting the transcriptomic data.

We have amended the text to specify the postnatal days used for RNA sequencing, see Line 123

(5) Regarding the scRNA-Seq data: were ACC-negative samples (isolated via FACS) also processed? A direct comparison between ACC+ and ACC- sampled microglia would significantly strengthen the claim that microglia are specifically attracted to ACCs. If these data are available, they would make an elegant and compelling addition to the manuscript.

We thank the reviewer for this important suggestion and agree that direct comparison of ACC+ and ACC− microglia would further strengthen the study. Unfortunately, ACC− populations were not processed for scRNA-seq in the current study because of the prioritisation of the rare ACC+ population. Nevertheless, several independent observations support the conclusion that microglia are preferentially associated with ACCs, including: (i) the enrichment of microglia within ACC-containing regions observed histologically, (ii) the spatial proximity analyses shown in Figure 3, and (iii) the distinct transcriptional profile of ACC-associated microglia identified by scRNA-seq.

We have also added a section to the discussion to highlight the need for a direct comparison of the ACC+ and ACC- transcriptomics profiles in future work, see Line 344.

(6) For the Ca2+ -imaging experiments, please briefly describe the staining protocol and specify which cell types (e.g., RGCs) were labeled within the Results text to assist the reader's immediate understanding.

We have added a short description of the labelling technique in the results, see Line 228

(7) The manuscript notes that MEA waves are evident in the graphs of Figure 7, but the raw wave data or representative traces are not shown. Including these (similar to those of the imaging waves) would provide necessary visual verification of the physiological phenomena described.

We have added a supplementary figure 3 which details stage 2 retinal waves recorded using MEAs.

(C) Interpretations and Logic

(8) The finding ' ...Wholemount staining revealed a broad centro-peripheral gradient of apoptosis; however, this apoptotic annulus was positioned more peripherally than the ACCs, SVP, and Hmox1-positive microglia (Figure 4B)' seems to contradict the HMOX1/Yo-Pro-1 stained microglia. It is not clear whether the authors aim to prove that this particular set of microglia phagocytise RGCs, or another set that lines up better with dying cells and does not show up on the HMOX-1 label. I believe the authors intend to show the time difference of the two events - cells dying and HMOX-1 microglia appear at the site later. I believe the logic is good; it may need a sentence pointing this out at the end of this paragraph.

We have added a statement in Line 182 which clarifies our intent to show that the *Hmox1* microglia phagocytose the dying RGCs after they initiate apoptotic mechanisms.

(9) If the authors intend to demonstrate a temporal lag between cell death and the appearance of HMOX1+ microglia as evidence of causality, a concluding sentence to this effect would greatly clarify the logic of this paragraph.

We have added a concluding sentence to the paragraph in Line 190 which indicates a causal link between RGC cell death and appearance of hmox1 positive microglia.

(D) Figures and Presentation

(10) The blood vessel staining in the final panel of Figure 1B is currently quite faint. Increasing the brightness/contrast for this panel would allow the reader to better appreciate the underlying architecture.

We have updated the panel in Figure 1B to match the brightness of the others of that series.

(11) Given that the peripherality of events in Figure 7 suggests a specific sequence, the authors should consider adding a summary timeline (P3-P6). A plot using curves (mean or median values), color-coded to match the corresponding events, would provide a much-needed visual synthesis of the data.

We agree with reviewer that the D1/2 metrics would benefit from more clarification to show the timeline of development more clearly. We have added another panel to Figure 7, which shows mean/standard deviation plots for each developmental measure using D1/2 as timelines. This allows the reader to better compare the progression of centrifugal spread more clearly.

(12) Please ensure that graph labels and axis titles are uniform in size across all figures. e.g., the labels in Figure 6 and several other graphs are currently too small to be legible in the PDF; these should be enlarged for better accessibility.

We have fixed the labels and axis titles to maintain readability across the paper

Minor comments

(1) In line 125, there is a missing closing parenthesis after the reference to Figure 1C.

We have fixed this error

(2) The specific algorithm used for the unsupervised cluster analysis has not been identified in the text. Please specify whether k-means, Louvain, or another method was employed to ensure reproducibility.

We have reworked the section detailing the cluster analysis to make clear we used Louvain-based clustering, see line 475.

(3) While it is appropriate to leave comprehensive technical details for the Methods section, a brief conceptual explanation of the D1, D2, and D3 metrics should be included in the Results text to aid general comprehension.

We have added a brief description of the D1/2 and D1/3 metrics when they are first mentioned in the results section, see line 243.

(4) In the Figure 7 schematic, the representation of the starburst amacrine cell should be revised to more accurately reflect its well-characterized morphology (e.g., thin primary and gradually thickening higher-order dendrites).

We have changed the SAC representation to better match the characteristics of that cell type.

(5) Throughout the manuscript (e.g., in lines 293-294), it is claimed that RGC apoptosis promotes the expression of PANX-1 hemichannels. While the data effectively demonstrate the release of purinergic molecules (e.g., ATP) via PANX-1 from dying cells, the evidence for an actual upregulation or increase in PANX-1 protein/mRNA levels is not explicitly shown. Please clarify whether the findings suggest increased activity of existing channels or a true increase in expression. If the latter is not empirically supported, the phrasing should be adjusted to reflect functional activation rather than de novo expression.

We agree with the author that our research shows a functional increase in PANX-1 and we have adjusted the language to match. In the introduction and discussion, we provide published evidence and experimental papers which describe the upregulation of the PANX-1 molecule in dying RGCs.

**Reviewer #2 (Recommendations for the authors):**

Savage et al. investigate the synchronization of retinal Ca2+ waves with developmental cell death, microglia activation, and vascular outgrowth. These developmental processes occur through a mechanism where apoptotic cells release ATP through Panx-1 channels to stimulate both Ca2+ retinal waves and microglia activation. Furthermore, the authors demonstrate the initiation of Ca2+ waves occurs at the leading edge of vascular growth in the developing retina. This manuscript proposes several novel ideas, such as ATP as the Ca2+ wave initiating cue, and the localization of the Ca2+ wave initiation to the leading edge of vascular growth. The main weakness of the manuscript is the overreliance on only two pharmacological agents to test their central hypotheses. These conclusions would be strengthened if, in addition to their pharmacological manipulations, they used genetic knockout models to perturb programmed cell death or ATP releases (i.e., BAX-KO, Panx-1 KO). In addition, the following comments should also be addressed:

We thank the reviewer for their nice summary and for highlighting the strengths of this work.

Specific comments:

(1) Line 128: Why would ACCs be involved in SVP guidance if they are trailing the leading edge of the vasculature? Would it be the other way around, where the leading edge of the vasculature would be trailing the ACCs?

At this point in the paper we are suggesting that the highly stereotyped position of the ACCs under the leading edge of the SVP indicates that they have a mechanistic involvement in SVP growth. Not that they are the direct cause of the expansion. As the paper progresses, we make clear that contrary to our original hypotheses which state the ACCs may cause or control the integrated development of the retina, they are a hallmark of the apoptotic RGCs in the periphery which are the chemogenic beacons for vascular growth being ‘decommissioned’ by the microglia which fine-tune vascular growth and create the ACCs.

(2) Line 137: Please state in the text and figure legend, at what age ACCs were isolated from the retina.

We have added the relevant information to Line 123

(3) Line 154: Why didn't RGCs form their own cluster? Why do the RBPMS+ cells appear across the entire dataset (Figure 2G)? Have previous investigations also shown engulfed cell transcriptomes appearing in the microglia clusters using scRNAseq?

Previous transcriptomic studies have demonstrated that phagocytic microglia can encapsulate transcripts originating from neurons and other neural cell types, which are detectable by RNA‑seq despite not belonging to a common microglial genetic signature. For example, Solga et al. showed that CNS microglia contain neuronal and oligodendrocyte‑specific mRNAs that localise within microglia but are not translated. This research group interpreted that this RNA is acquired through phagocytosis or macropinocytosis of surrounding neural cells (Solga et al., 2015). Similarly, in zebrafish, synapse‑engulfing microglia identified in situ display neuronal and synaptic gene expression in single‑cell RNA‑seq profiles, consistent with engulfed neuronal material contributing to the detected transcriptome (Sliva et al., 2021). In line with these observations, and given our FACS strategy enriching autofluorescent ACCs rather than intact RGCs, we interpret the widespread Rbpms expression across ACC‑associated clusters as an expected consequence of microglial engulfment of apoptotic RGCs, rather than evidence for a distinct population of viable RGCs that failed to form a separate cluster.

Solga, A.C., Pong, W.W., Walker, J., Wylie, T., Magrini, V., Apicelli, A.J., Griffith, M., Griffith, O.L., Kohsaka, S., Wu, G.F. and Brody, D.L., 2015. RNA‐sequencing reveals oligodendrocyte and neuronal transcripts in microglia relevant to central nervous system disease. Glia, 63(4), pp.531-548.

Silva, N.J., Dorman, L.C., Vainchtein, I.D., Horneck, N.C. and Molofsky, A.V., 2021. In situ and transcriptomic identification of microglia in synapse-rich regions of the developing zebrafish brain. Nature communications, 12(1), p.5916.

We have incorporated this information into the discussion in Line 329

(4) Figure 4C-H: The data in the figure would be strengthened if the authors added quantification for their co-localization images.

We thank the reviewer for this important suggestion and agree that quantification of the co-localisation would further strengthen the study. Unfortunately, we are limited in conducting further experiments since the grant has been completed and the Sernagor lab is in the process of shutting down following her passing.

(5) Figure 5A-I: The error bars are quite large. The figure legend says they represent SEM, but are you sure they don't represent standard deviation (SD)?

The large SEM bars reflect substantial biological variability across retinas and across individual microglia, which is expected for morphometric measures such as circularity, perimeter, branch number and total skeleton length, as well as for counts of rare cell populations (Hmox1+ microglia, YO‑PRO‑1+ cells and double‑positive cells). Importantly, despite this variability, the effects of probenecid and PSB‑0739 on microglial morphology and on the frequencies of apoptotic and double‑positive cells remain statistically robust in our non‑parametric ANOVA and post‑hoc tests, as indicated by the reported P‑values.”

For panels where the distributions were clearly non‑Gaussian, we used non‑parametric statistics (Kruskal‑Wallis ANOVA), reporting medians and 95% confidence intervals, and we retained SEM in Figure 5 for consistency with the original plotting routine while clarifying this choice in the legend and Methods.

(6) Figure 5: What are these measurements made at? Please add the age to the results section and the figure legend.

We have added the postnatal day of the animals used.

(7) Line 236-239: There is no mention of the use of Probenecid in the text and in Figure 6B. This treatment of Ca2+ waves should be mentioned before line 245.

We have added a brief description of probenecid application in Line 227.

(8) Figure 6: The order of this figure and the results section may be clearer if panels C-F were switched with panels G-J.

We thank the reviewer for their suggests to improve the flow of the results section and figure 6. We have swapped the panels as suggested and amended the text to fit the new flow.

(9) For the discussion section: Why does probenecid only affect the wave initiation at the P3 timepoint, but not later timepoints, P4-P6.

We have added some text in the discussion to explain our findings of differential effects of PANX-1 blockade across the P3-6 timeline. See Line 404.

Editorial revisions:

(1) Line 92-96: Citation needed.

We have added appropriate citations for this section.

(2) Figure 1F: Please consider changing the color scheme from green/red to green/magenta for colorblind readers.

We have changed the image LUT

(3) Figure 3A: This figure may benefit from separating out each channel separately (Iba1 and ACC) and then having a "merge" panel.

We have separated out the panels in Figure 3A

(4) Figure 4E, H: There is no label for the immunomarkers in Figure 4H. There is no inset in Figure 4E as mentioned in the figure legend. However, it appears that Figure 4H is a magnified image of Figure 4G and not Figure 4E.

We have fixed the error in the figure legend and included an inset indicator in Figure 4G. We have added immunomarkers to Figure 4H

(5) Line 198: "SAC" should be "SACs".

We have fixed this error
